# SETDB1 and HUSH modulate Xist RNA levels during establishment of X chromosome inactivation

**DOI:** 10.1038/s41467-026-71569-8

**Published:** 2026-04-09

**Authors:** Mafalda Almeida, Guifeng Wei, Adam D. Cawte, Tatyana B. Nesterova, Neil Brockdorff

**Affiliations:** Developmental Epigenetics lab, Department of Biochemistry, University of Oxford, Oxford, UK

**Keywords:** Dosage compensation, Histone post-translational modifications

## Abstract

The non-coding RNA Xist mediates X chromosome inactivation (XCI) in mammals, functioning *in cis* to direct heterochromatin formation over a single X chromosome in cells of early female embryos. Prior studies have determined that Xist transcription and turnover are modulated to maintain the correct level of Xist RNA to inactivate a single X chromosome. The underlying molecular mechanisms are poorly understood. In this study we demonstrate that RNA-dependent recruitment of the H3K9me3 methyltransferase SETDB1 and the HUSH complex across the transcribed *Xist* locus functions to suppress Xist transcription during establishment of XCI. Thus, degron-mediated acute depletion of SETDB1 or core HUSH subunits abrogates allelic H3K9me3 deposition, increasing Xist transcription, accumulation of Xist RNA, and the rate of X-linked gene silencing. Our findings support a model in which the SETDB1/HUSH pathway coordinates Xist transcription rates to maintain optimal Xist RNA levels, ensuring the silencing of a single X chromosome.

## Abstract (web summary)

Regulation of X chromosome inactivation in mammals requires precise control of the levels of the non-coding RNA Xist. Almeida *et al*. describe an autoregulatory mechanism in which nascent Xist transcripts recruit the repressor SETDB1 to dampen Xist gene transcription and counter Xist overexpression.

## Introduction

X chromosome inactivation (XCI) in mammals evolved to equalise levels of X-linked gene expression in XX females relative to XY males^1^. The XCI process is orchestrated by a ~ 17 kb *cis*-acting non-coding RNA termed Xist^2^. Our current understanding of Xist function has come largely from studies in mouse embryos and from in vitro XX female mouse embryonic stem cell (mESC) models. Here Xist transcription initiates on one of the two X chromosomes in cells of early XX female embryos/mESCs. A complex circuitry comprising *cis*-regulatory elements, trans-acting factors and non-coding RNAs ensures monoallelic Xist transcription specifically in cells of female embryos^3^. Upon initiation of transcription, Xist RNA accumulates and spreads *in cis*, recruiting chromatin-modifying factors that establish a repressive heterochromatic state over the entire X chromosome. Xist RNA concentrates over ~ 60 sites, which correspond to gene-rich regions^4–6^. Recent evidence suggests that Xist overexpression results in spill-over onto neighbouring autosomes, which in some instances may be linked to physiological regulation of autosomal genes^7,8^. In this context, it is important to note that there is feedback control between Xist RNA turnover and transcription that serves to modulate Xist RNA abundance^9,10^, and that this is likely important in controlling Xist levels through the cell cycle and/or cell differentiation and development.

Chromosome silencing by Xist RNA is instigated by the RNA-binding proteins SPEN and hnRNPK, which recognise tandem repeat elements in Xist RNA designated A- and B/C-repeats, respectively. SPEN recruits the NCoR-HDAC3 complex, which catalyses inactive X (Xi) chromosome histone deacetylation^11–15^. hnRNPK, on the other hand, recruits the Polycomb system, comprising PRC1 and PRC2 complexes that catalyse the repressive histone post-translational modifications (PTMs) H2AK119ub1 and H3K27me3, respectively^12,16,17^. Other factors that function downstream of these pathways to reinforce and maintain Xi include the chromosomal protein SMCHD1^18^, the DNA methylation machinery that catalyses CpG island promoter DNA methylation on Xi^19^, and the variant histone macroH2A^20^.

An additional factor implicated in the maintenance of XCI is the histone methyltransferase SETDB1, which catalyses the repressive histone PTM H3K9me3^21,22^. The mechanistic basis for SETDB1 function in this context is not well understood, and there are some opposing findings, notably that immunofluorescence (IF) analysis indicates no obvious enrichment of H3K9me3 on Xi in mouse^23^. Interestingly, this finding contrasts with Xi in human and some other mammalian species, where H3K9me3 enrichment is apparent^23–26^. In this study, we set out to further investigate the role of SETDB1 in XCI using a degron-based acute depletion strategy. Unexpectedly, we find that in addition to its previously proposed role in XCI maintenance, SETDB1 modulates Xist RNA levels at the onset of XCI. This novel function involves nascent RNA-dependent recruitment of HUSH/SETDB1 and H3K9me3 deposition across the *Xist* locus to suppress Xist transcription. We discuss our findings in the context of evidence for the important role of feedback control of Xist RNA levels in XCI regulation.

## Results

### SETDB1 depletion accelerates X chromosome inactivation

**Fig. 1:**
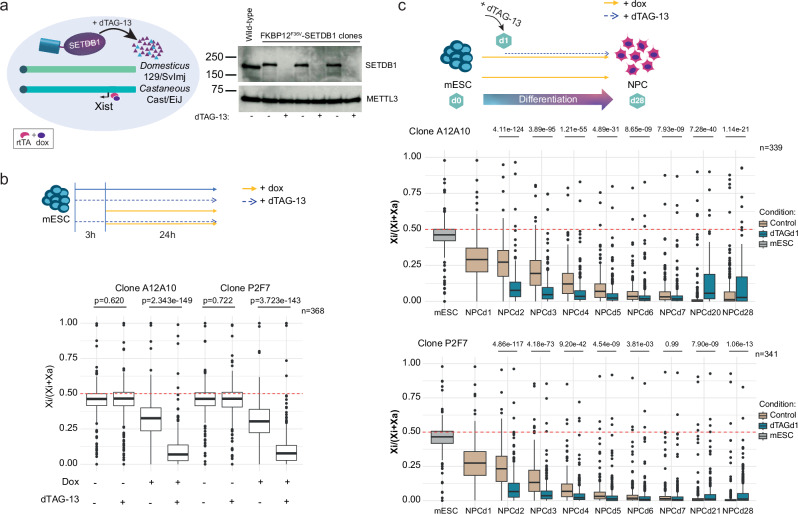
SETDB1 depletion leads to accelerated X chromosome silencing. **a** Schematic illustrating Cast x 129 F1 mESC model engineered to allow depletion of endogenous SETDB1 using dTAG-13. Western blot shows protein depletion after 2 h of dTAG-13 treatment. METTL3 is a loading control. Molecular markers indicated in kDa. Source data are provided as a Source Data file. **b** Top: Schematic illustrating the experimental setup for SETDB1 depletion prior to Xist expression in mouse ES cells. Bottom: Boxplots showing allelic ratio (ranging from 0 to 1 with values below 0.5 indicating Xi silencing) of X-linked genes (*n* = 368) from ChrRNA-seq analysis of two independent clones containing a FKBP12^F36V^-tagged SETDB1 (A12A10 and P2F7) using mESCs in different conditions indicated at the bottom, demonstrating accelerated silencing following SETDB1 depletion. The red dashed line indicates an allelic ratio of 0.5. *P*-values were calculated using a two-sided paired *t* test. In boxplots, centre lines indicate the median, box limits indicate the first and third quartiles, and whiskers indicate 1.5 × the interquartile range (IQR). Source data are provided as a Source Data file. **c** Top: Schematic illustrating the experimental setup for SETDB1 depletion during differentiation of mESCs into NPCs. dTAG-13 is added 24 h after Xist induction and differentiation. Bottom: Boxplots show Xi silencing (grey), which is accelerated following SETDB1 depletion (blue), across the differentiation time course. Allelic ratios (ranging from 0 to 1) of X-linked genes (*n* = 339 and *n* = 341) are shown from ChrRNA-seq analysis of two independent clones containing a FKBP12^F36V^-tagged SETDB1 (A12A10 and P2F7) upon differentiation of mESCs into NPCs. The red dashed line indicates an allelic ratio of 0.5. *P*-values were calculated using a two-sided paired *t* test. In boxplots, centre lines indicate the median, box limits indicate the first and third quartiles, and whiskers indicate 1.5 × the interquartile range (IQR). Source data are provided as a Source Data file.

To investigate the function of the H3K9me3 methyltransferase SETDB1 in XCI, we used the dTAG system to acutely deplete SETDB1 in a previously described interspecific X^Cast^X^129^ mouse embryonic stem cell (mESC) line, iXist-ChrX^Cast^, which has a doxycycline inducible promoter driving expression of the Xist allele on the *Mus castaneus* (Cast) derived X chromosome^27^. CRISPR/Cas9-facilitated homologous recombination was used to derive independent cell lines with SETDB1 tagged at the N-terminus with an FKBP12^F36V^ degron. Addition of dTAG-13 reagent to the engineered mESCs resulted in SETDB1 depletion within ~ 2 h (Fig. 1a).

To assay effects on X-linked gene silencing, we first performed ChrRNA-seq analysis of undifferentiated mESCs after SETDB1 depletion and 24 h of Xist induction. Single-nucleotide polymorphisms (SNPs) between the parental *Mus castaneus* (Cast) and *Mus musculus* (129) genomes enable quantification of reads assigned to the Xist-expressing X^Cast^ and the active X^129^ chromosomes. Unexpectedly, we observed a dramatic increase or acceleration of X-linked gene silencing following SETDB1 depletion using three independently derived mESC lines (Fig. 1b and Supplementary Fig. 1a). Analysis of X-linked genes categorised according to their rate of silencing, pre-XCI expression levels, and association with H3K27me3 modified chromatin indicates that accelerated silencing affects all X-linked genes equivalently (Supplementary Fig. 1b).

We next analysed the effect of SETDB1 depletion over a time-course following Xist induction and in vitro differentiation into neuronal precursor cells (NPC). Because SETDB1 is essential for long-term cell viability in undifferentiated mESCs^28^, Xist expression and differentiation were initiated 24 h prior to SETDB1 depletion. X-linked gene silencing was then assayed in three independent clones at day 2 of differentiation and timepoints thereafter (Fig. 1c and Supplementary Fig. 1c). Consistent with findings in undifferentiated mESCs, SETDB1 depletion resulted in highly accelerated silencing relative to untreated samples at all timepoints up to day 7.

In fully differentiated NPCs (timepoints later than NPC d20) we observed a modest reactivation of Xi genes in dTAG treated cultures relative to untreated controls for three independent cell lines (Fig. 1c and Supplementary Fig. 1c), in line with previous findings suggesting a role for SETDB1 in XCI maintenance^21,22^. Further analysis of Xi gene reactivation in relation to previously defined Xi gene categories indicates significant overlap with genes for which complete silencing is either dependent or partially-dependent on the chromosomal protein SMCHD1 (Supplementary Fig. 1d), suggesting cross-talk between SETDB1 and SMCHD1 pathways in XCI maintenance.

### Accelerated silencing correlates with increased Xist gene transcription

**Fig. 2:**
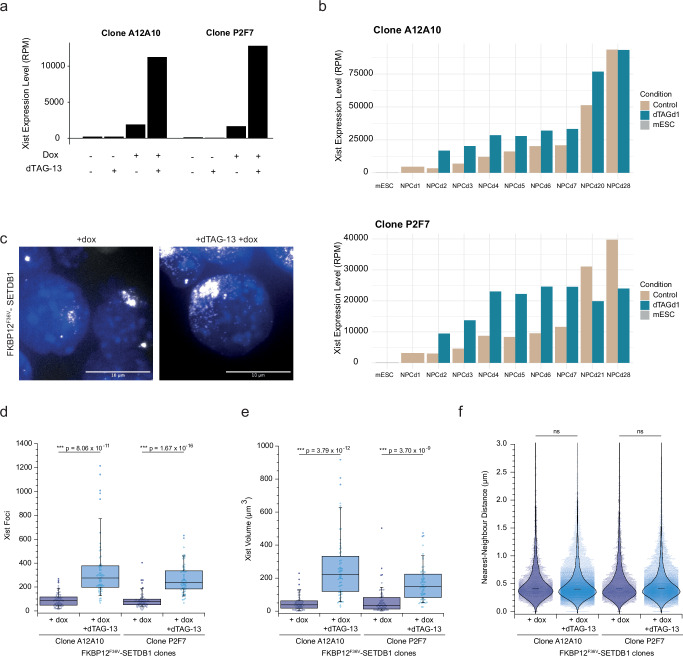
Depletion of SETDB1 results in elevated levels of Xist RNA. **a** Bar plot showing Xist RNA levels (RPM, reads per million mapped reads) from ChrRNA-seq analysis of two independent mESC clones with FKBP12^F36V^-tagged SETDB1 (A12A10 and P2F7) using mESCs in different conditions as indicated at the bottom and matching Fig. 1b. Source data are provided as a Source Data file. **b** Bar plots showing increased Xist RNA levels (RPM, reads per million mapped reads) following SETDB1 depletion. ChrRNA-seq analysis is from two independent mESC clones with FKBP12^F36V^-tagged SETDB1 (A12A10 and P2F7) with NPC differentiation timepoints matching Fig. 1c. Source data are provided as a Source Data file. **c** Representative iSIM images (maximum projections) of Xist RNA-FISH (in white) in control and SETDB1-depleted mESCs, matching conditions represented in Figs. 1b, 2a. DNA is counterstained with DAPI (in blue). Scale bar, 10 μm. **d** Boxplot showing increased number of Xist foci following SETDB1 depletion determined from quantification of Xist RNA-FISH in two independent clones containing a FKBP12^F36V^-tagged SETDB1 (A12A10 and P2F7) after 24 h doxycycline induction with or without addition of dTAG-13. Statistical analysis was conducted using an unpaired single-tailed student’s *T* test between individual clones with or without dTAG-13 to quantify the significance of SETDB1 removal across the population of single cells. The numbers of cells analysed for A12A10 were 60 and 61 and for P2F7 were 62 and 61 cells in the Control and + dTAG-13 conditions, respectively. In boxplots, centre lines indicate the median, box limits indicate the first and third quartiles, and whiskers indicate 1.5 × the interquartile range (IQR). Source data are provided as a Source Data file. **e** Boxplot showing Xist RNA cloud volume increases following SETDB1 depletion. Volume (in μm^3^) is calculated from Xist RNA-FISH analysis of two independent mESC clones with FKBP12^F36V^-tagged SETDB1 (A12A10 and P2F7) after 24 h doxycycline induction with or without addition of dTAG−13. Similarly, statistical analysis used a student’s T-test and took into account the difference between treatment conditions with or without dTAG-13 and quantified the significance across the population of single cells. As in Fig. 2d, the number of cells analysed were 60 and 61 for A12A10 and 62 and 61 for P2F7 in the Control and + dTAG-13 conditions, respectively. In boxplots, centre lines indicate the median, box limits indicate the first and third quartiles, and whiskers indicate 1.5 × the interquartile range (IQR). Source data are provided as a Source Data file. **f** Violin plot showing density of Xist molecules in clouds is unchanged following SETDB1 depletion, as inferred from nearest-neighbour distance analysis (in μm) of Xist molecules from RNA-FISH of two independent mESC clones containing a FKBP12^F36V^-tagged SETDB1 (A12A10 and P2F7) after 24 h doxycycline induction with or without addition of dTAG-13. No significant change was observed between nearest-neighbour distances upon the removal of SETDB1 as quantified using a student’s *T* test comparing Control and + dTAG-13 conditions for two independent clones. For clone A12A10, 4003 and 14758 foci were quantified from 60 and 61 cells in the Control and + dTAG-13 conditions, respectively. For clone P2F7, 3897 and 11606 foci were quantified from 62 and 61 cells in the Control and + dTAG-13 conditions, respectively. Source data are provided as a Source Data file.

We went on to investigate the novel finding that SETDB1 depletion results in accelerated XCI. In recent work, we observed accelerated XCI resulting from an approximately 2-fold increase in Xist RNA levels following depletion of the *N*^6^-methyladenosine (m^6^A) writer complex and the nuclear exosome targeting (NEXT) complex^29^. Elevated Xist RNA levels are attributable to the function of these factors in promoting Xist RNA turnover. To investigate if increased Xist RNA levels could also account for accelerated XCI following SETDB1 depletion, we estimated Xist RNA abundance from ChrRNA-seq datasets. This indicated a marked 4-5 fold increase in Xist RNA levels in independent mESC lines (Fig. 2a), and similarly, significantly elevated levels of Xist RNA during the first 7 days of NPC differentiation (Fig. 2b). The level of expression of the rtTA transgene, required for the function of the inducible Tet-ON system, is broadly unaffected in response to SETDB1 depletion, indicating that this is not the reason for increased Xist RNA levels in mESC and NPC (Supplementary Fig. 2a, b). These results together support the inference that accelerated XCI following SETDB1 depletion is linked to elevated levels of Xist RNA.

We went on to investigate Xist RNA abundance following SETDB1 depletion using an orthogonal approach, RNA-FISH coupled to super-resolution imaging and quantitative image analysis. Qualitative assessment of RNA-FISH data indicated that Xist RNA domains are significantly enlarged (Fig. 2c), and this was confirmed by quantitative image analysis. Thus, the number of Xist RNA foci and the volume of Xist domains increase around ~ 4-5 fold (Fig. 2d, e), a similar magnitude to that observed in the ChrRNA-seq data. The density of Xist molecules, estimated from nearest neighbour distances (Fig. 2f), is broadly unchanged. Together, these observations suggest that Xist localisation on the X chromosome is largely retained but that there are additional newly occupied sites either on the X chromosome and/or on neighbouring chromosomes as a consequence of Xist RNA spill-over. In relation to the latter possibility, we did not find evidence for repression of contiguous autosomal genes (Supplementary Data 1).

**Fig. 3:**
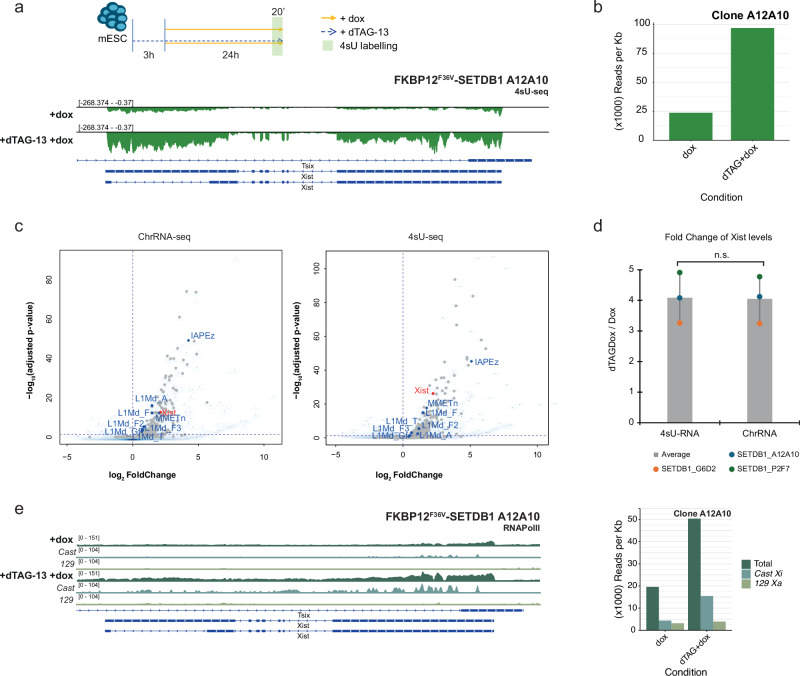
SETDB1 modulates transcription at the Xist locus. **a** Top: Schematic illustrating the experimental setup for SETDB1 depletion prior to Xist expression in mESCs, followed by 4sU labelling of newly transcribed RNA for 4sU-seq analysis. Bottom: A representative snapshot showing increased 4sU-seq reads for Xist RNA SETDB1 depleted ( + dTAG-13+dox) relative to control ( + dox) conditions in FKBP12^F36V^-tagged SETDB1 mESCs (clone A12A10). **b** Bar plot showing quantification of the number of Xist reads from 4sU-seq in control ( + dox) and SETDB1 depleted ( + dTAG-13 + dox) conditions in FKBP12^F36V^-tagged SETDB1 mESCs (clone A12A10), shown in Fig. 3a. Source data are provided as a Source Data file. **c** Scatter plot illustrating differential expression analysis of gene and repeat element derived RNAs. The background shows a smoothed density scatter plot representing gene expression changes, while individual grey dots indicate RNAs derived from repeat elements and Xist. Xist RNA is highlighted in red, and several selected known SETDB1 targets, i.e., LINE1- and ERV-derived RNAs, are shown in blue. Left, ChrRNA-seq; right, 4sU-seq. X-axis, log₂ fold change between SETDB1 degron and control conditions; Y-axis, –log₁₀ transformed adjusted p-value. Vertical dashed line, no expression change; Horizontal dashed line, significance threshold. Source data are provided as a Source Data file. **d** Bar plot showing the average fold increase in Xist RNA levels following SETDB1 depletion, calculated from ChrRNA-seq and 4sU-seq datasets analysis of three independent FKBP12^F36V^-tagged SETDB1 mESC lines. Bar charts indicate the average, and error bars show standard deviation. Individual datapoints are shown in colour and labelled according to FKBP12^F36V^-tagged SETDB1 clone name. *p*-value was calculated by two-sided t test (0.95). Source data are provided as a Source Data file. **e** Representative snapshot showing increased RNAPII cChIP-seq reads over the Xist locus in SETDB1 depleted ( + dTAG-13 + dox) relative to control ( + dox) conditions in FKBP12^F36V^-tagged SETDB1 mESCs (clone A12A10). Light shades represent reads with SNPs that can be assigned to either allele (*Cast* and *129*) using SNPsplit. *Cast* corresponds to the Xi in this cell line. Bar plot shows quantification of the number of reads from RNAPII cChIP-seq over the Xist locus in control ( + dox) and SETDB1 depleted ( + dTAG-13+dox) conditions in FKBP12^F36V^-tagged SETDB1 mESCs (clone A12A10). Source data are provided as a Source Data file.

To determine the basis for increased Xist RNA levels following SETDB1 depletion, we used 4sU-seq^30^ to assess the rate of transcription at the Xist locus. In undifferentiated mESCs after 24 h of Xist RNA induction, we observed an approximately 4-fold increase in the Xist transcription rate as a result of SETDB1 depletion (Fig. 3a,b and Supplementary Fig. 3a). This is similar to the increase in steady-state levels of Xist RNA (Fig. 3c, d), indicating that increased transcription rates are the sole basis for higher levels of Xist RNA. Both steady-state RNA and de novo transcription rates were also seen to increase for a small proportion of protein-coding RNAs and transcripts from LINE-1 (L1) and endogenous retroviral elements (ERV) (Fig. 3c). These latter observations are consistent with known functions of SETDB1 in mESCs^31–33^.

In a parallel set of experiments, we performed calibrated ChIP-seq (cChIP-seq) analysis for RNAPII following 24 h Xist RNA induction with/without SETDB1 depletion. A proportion of the cChIP-seq reads include SNPs, enabling allele specific analysis, as described previously^34^. Consistent with 4sU-seq analysis we observed a marked increase in RNAPII occupancy over the Xist locus (Fig. 3e and Supplementary Fig. 3b–d). In both untreated and dTAG-treated samples, RNAPII occupancy was highest immediately downstream of the TSS, indicative of promoter proximal pausing. The pausing index (the ratio of the read density of promoter-proximal RNAPII to the read density of gene body RNAPII) is reduced following SETDB1 depletion (Supplementary Fig. 3c), suggesting that an increased elongation rate contributes to higher levels of Xist RNA. However, cChIP-seq analysis of Ser5 and Ser2 CTD phosphorylated forms of RNAPII associated with the initiation and elongation phases of RNAPII transcription, respectively, indicates that both increase following SETDB1 depletion (Supplementary Fig. 3d). These results thus demonstrate that elevated levels of Xist RNA following SETDB1 depletion are the result of increased rates of transcription initiation and elongation at the Xist locus due to increased RNAPII engagement.

### Accumulation of SETDB1-dependent H3K9me3 over the expressed Xist allele

**Fig. 4:**
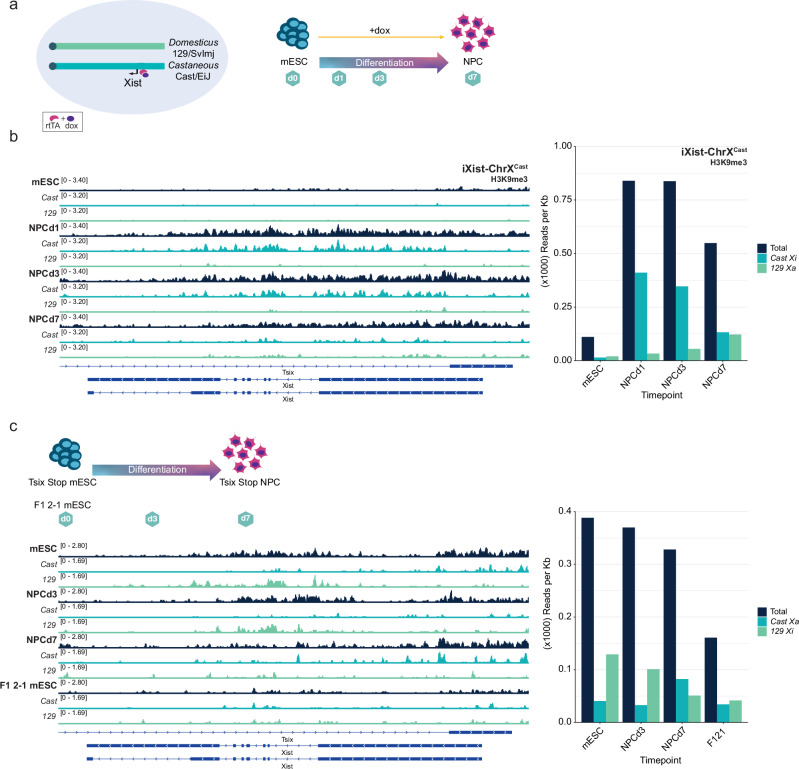
H3K9me3 accumulates on the transcribed Xist allele. **a** Schematic illustrating the experimental setup for Cast x 129 F1 mESC model, iXist-chrX^Cast^, in which the inducible Xist allele is on the Cast X chromosome. **b** Representative snapshot of allelic cChIP-seq analysis for H3K9me3 showing increased levels across the expressed Xist locus in NPC differentiation of iXist-chrX^Cast^ mESCs. Lighter shades represent reads with SNPs allowing assignation to each allele (Cast and 129) using SNPsplit. Cast corresponds to the Xi in this mESC line. Quantification of the total number of reads and reads assigned to Cast Xi and 129 Xa are shown in the barplot. Source data are provided as a Source Data file. **c** Top: Schematic illustrating the experimental setup for NPC differentiation and timepoint collection of TsixStop and F1 2-1 mESCs. Bottom: Representative snapshot of allelic cChIP-seq for H3K9me3 showing increased levels over the expressed Xist locus in TsixStop mESC and upon NPC differentiation. Data for the parental cell line F1 2-1 mESC where there is no Xist expression is shown as a control. Lighter shades represent reads assigned to each allele (Cast and 129) using SNPsplit. 129 corresponds to the inactive X chromosome in the TsixStop cell line. The bar plot shows quantification of the number of H3K9me3 cChIP-seq reads over the Xist locus in F1 2-1 mESCs as well as TsixStop mESCs and NPCs, including the quantification of reads assigned to each allele (Cast and 129). Source data are provided as a Source Data file.

Transcriptional repression by SETDB1 is mediated by catalysis of the histone PTM H3K9me3^35,36^. Accordingly, we carried out allelic cChIP-seq analysis to determine H3K9me3 levels over the Xist locus in parental iXist-ChrX^Cast^ XX mESCs following Xist induction and NPC differentiation. Interestingly, this analysis revealed H3K9me3 accumulation commensurate with ongoing Xist gene expression specifically over the expressed allele (Fig. 4a, b). Moreover, analysis of a second XX mESC line, iXist-ChrX^129^, in which the inducible Xist allele is present on the 129 strain-derived X chromosome, showed a reciprocal asymmetric pattern of H3K9me3 accumulation (Supplementary Fig. 4a, b). iXist-ChrX^129^ cChIP-seq analysis was further validated using a different H3K9me3-specific antibody (Supplementary Fig. 4c). In both XX mESC lines, allelic H3K9me3 accumulation was evident through the first 3 days of NPC differentiation and diminished thereafter (Fig. 4b and Supplementary Fig. 4b, c).

The XX mESCs used in the aforementioned experiments make use of the heterologous doxycycline-inducible TetOn promoter to drive monoallelic Xist expression. To determine if H3K9me3 across Xist also occurs when monoallelic Xist expression is driven by the native promoter, we analysed interspecific TsixStop XX mESCs in which Xist expression from the native promoter is strongly biased towards the 129 X chromosome as a result of a premature transcription termination site engineered into the 129 allele of the antisense RNA Tsix^37,38^. ChrRNA-seq analysis validated Xist upregulation and Xi gene silencing during NPC differentiation of TsixStop XX mESCs (Supplementary Fig. 4d). Of note, analysis of Xist RNA levels showed that there is a degree of leaky expression in undifferentiated TsixStop mESCs relative to parental F1 2-1 XX mESCs (Supplementary Fig. 4d), as reported previously^38^. We went on to perform cChIP-seq analysis for H3K9me3 during early stages of NPC differentiation in these models. In line with the results obtained using TetOn inducible Xist expression, H3K9me3 accumulation was clearly evident and occurred predominantly over the expressed (129) Xist allele (Fig. 4c and Supplementary Fig. 4e). We also observed allelic H3K9me3 in undifferentiated TsixStop mESCs, likely attributable to the leaky Xist expression. Consistent with this interpretation, H3K9me3 was at near background levels and indistinguishable between the two alleles in F1 2-1 XX mESCs, the parental line from which Tsix Stop mESCs were derived (Fig. 4c and Supplementary Fig. 4e). Together, these results demonstrate that H3K9me3 accumulates over the expressed Xist allele in response to transcription driven by either heterologous or native promoters.

**Fig. 5:**
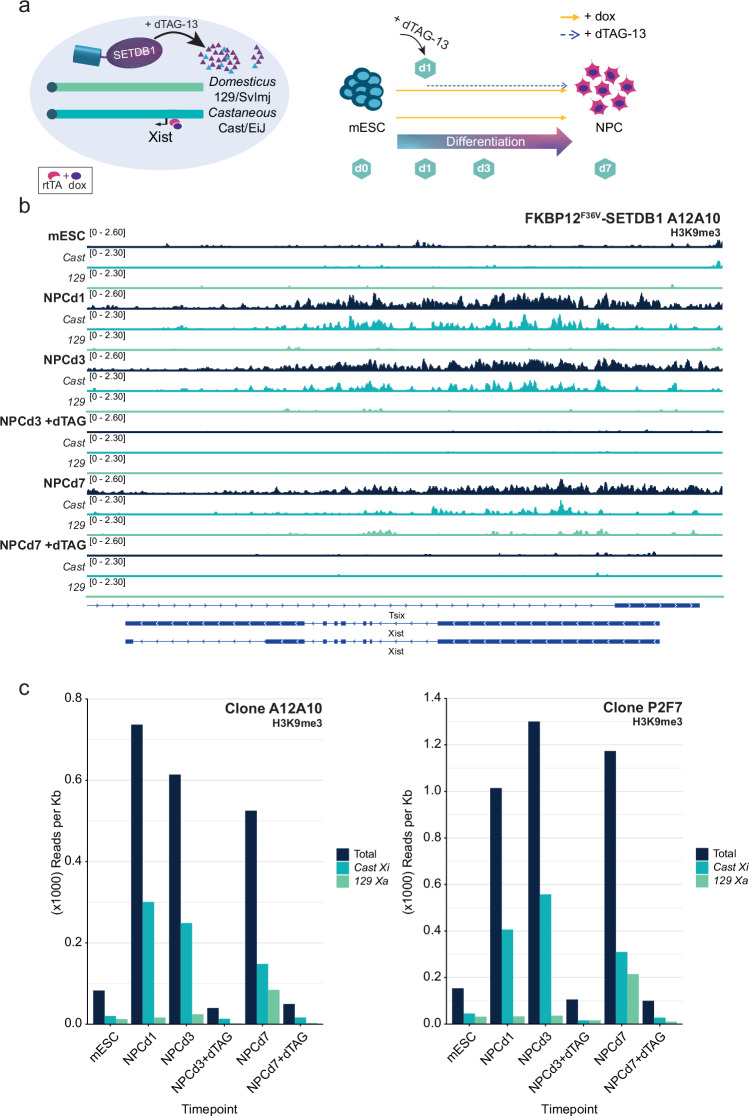
H3K9me3 deposition on the transcribed Xist allele is SETDB1 dependent. **a** Schematic illustrating the experimental setup for SETDB1 depletion during differentiation of mESCs into NPCs and timepoint collection for H3K9me3 cChIP-seq experiment in a FKBP12^F36V^-tagged SETDB1 mESCs. dTAG-13 is added 24 h after Xist induction and differentiation setup. **b** A representative snapshot showing H3K9me3 gain on the expressed Xist allele is SETDB1 dependent. cChIP-seq reads over the Xist locus are shown for different timepoints in a FKBP12^F36V^-tagged SETDB1 mESC line (clone A12A10) upon differentiation into NPCs in the presence or absence ( + dTAG) of SETDB1. Lighter shades represent reads assigned to each allele (Cast and 129) using SNPsplit. Cast corresponds to the Xi in this cell line. **c** Bar plots showing quantification of reads for H3K9me3 cChIP-seq in two independent FKBP12^F36V^-tagged SETDB1 mESC lines (clone A12A10 -matching tracks in Fig. 5b - and P2F7) upon differentiation into NPCs, including quantification of reads assigned to each allele (Cast Xi and 129 Xa). Source data are provided as a Source Data file.

We went on to determine if transcription-dependent H3K9me3 accumulation at the Xist locus is dependent solely on SETDB1, using FKBP12^F36V^-tagged SETDB1 XX mESC lines described above. As shown in Fig. 5a–c, SETDB1 depletion fully abrogated transcription-dependent allelic H3K9me3 over Xist in NPC differentiated cells. We conclude that SETDB1 is critically required for H3K9me3 deposition over the Xist locus and that other H3K9me3 methyltransferases are not able to compensate for its absence. Equivalent results were obtained for H3K9me3 cChIP-seq analysis of undifferentiated XX mESCs after 24 h Xist induction (Supplementary Fig. 5a–c). This latter result indicates that transcription-dependent H3K9me3 accumulation over Xist is independent of cellular differentiation.

### A role for the HUSH complex in targeting SETDB1 to the Xist locus

**Fig. 6:**
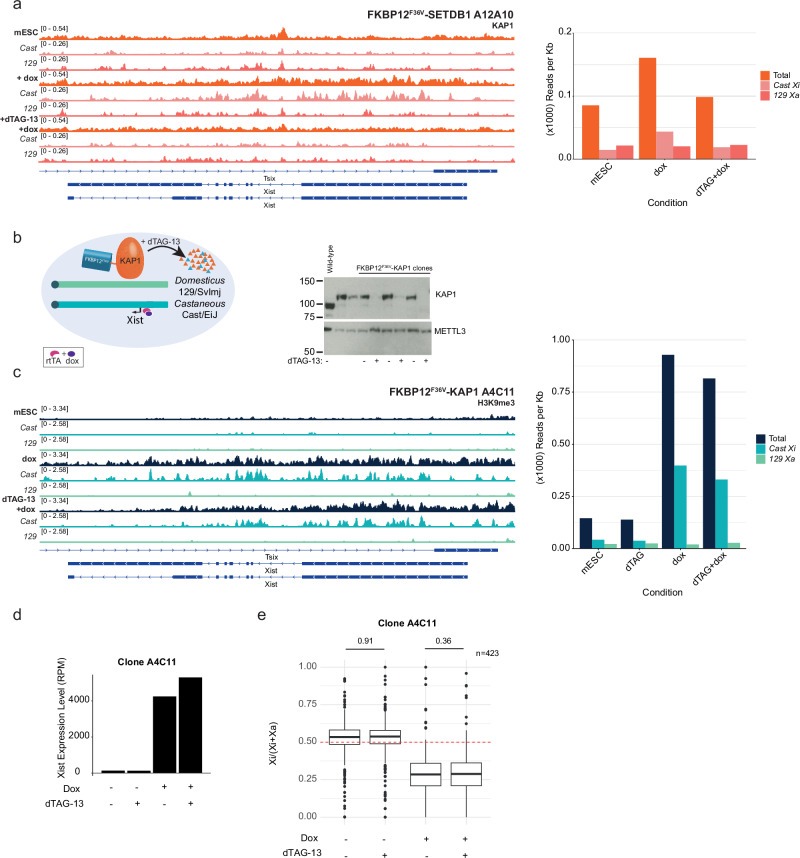
SETDB1-dependent accumulation of KAP1 on the transcribed Xist allele. **a** Representative snapshot of ChIP-seq analysis showing increased KAP1 levels over the expressed Xist locus are SETDB1 dependent, (compare control +dox with dTAG-13+dox) using FKBP12^F36V^-tagged SETDB1 mouse mESCs (clone A12A10). Lighter shades represent reads assigned to each allele (Cast and 129) using SNPsplit. Cast corresponds to the Xi in this cell line. Quantification of the total number of reads and reads assigned to Cast and 129 alleles is shown in the bar plot. Source data are provided as a Source Data file. **b** Schematic illustrating Cast x 129 F1 mESC model engineered to allow depletion of KAP1 using dTAG-13. Western blot shows protein depletion after 2 h of dTAG-13 treatment. The three clones to the right (labelled) were used herein. METTL3 is a loading control. Molecular markers indicated in kDa. Source data are provided as a Source Data file. **c** Representative snapshot of cChIP-seq analysis showing depletion of KAP1 (clone A4C11 cell line) has no effect on H3K9me3 levels over the expressed Xist locus (compare + dox with + dTAG-13 + dox conditions) in FKBP12^F36V^-tagged KAP1 mESCs. Lighter tracks represent reads assigned to Cast and 129 alleles using SNPsplit. Cast corresponds to the Xi in this cell line. Quantification of the total number of reads and reads assigned to Cast and 129 alleles is shown in the bar plot on the right. Source data are provided as a Source Data file. **d** Bar plot showing Xist RNA levels (RPM, reads per million mapped reads) from ChrRNA-seq analysis of FKBP12^F36V^-tagged KAP1 mESCs (clone A4C11) are unaffected by KAP1 depletion (conditions as indicated at the bottom). Source data are provided as a Source Data file. **e** Boxplot showing the allelic ratio (ranging from 0 to 1) of X-linked genes (*n* = 423) from ChrRNA-seq analysis of a FKBP12^F36V^-tagged KAP1 mESCs (clone A4C11), indicating KAP1 depletion has no effect on the rate of silencing. Different conditions indicated at the bottom. The red dashed line indicates an allelic ratio of 0.5. *P*-values were calculated using a two-sided paired t-test. In boxplots, centre lines indicate the median, box limits indicate the first and third quartiles, and whiskers indicate 1.5 × the interquartile range (IQR). Source data are provided as a Source Data file.

Two principal pathways for recruitment of SETDB1 are sequence-specific KRAB-ZFPs that direct SETDB1 to transposable elements via interaction with the adaptor protein TRIM28/KAP1 (KAP1 henceforth)^39–42^, and the human silencing hub (HUSH) complex, which directs SETDB1 to invasive DNA elements, including endogenous retroviruses, LINE-1 retrotransposons, pseudogenes, and large intronless genes^43,44^. Interestingly, a recent study revealed an RNA-dependent pathway for HUSH recruitment^43,45^. We set out to determine if these pathways contribute to H3K9me3 accumulation over the expressed Xist allele, initially focussing on the classical KAP1-mediated pathway. Allelic ChIP-seq analysis for the SETDB1 interacting protein KAP1 in XX mESCs with FKBP12^F36V^-tagged SETDB1 demonstrated broad association across the locus, preferentially on the expressed allele (Fig. 6a and Supplementary Fig. 6a). However, KAP1 association over Xist was lost following SETDB1 depletion, suggesting SETDB1 is required for KAP1 recruitment. This is the converse of what is observed at conventional KRAB-ZNF targets, where KAP1 is recruited independent of SETDB1.

To directly test the role of KAP1 accumulation over the expressed Xist allele we developed XX mESCs with FKBP12^F36V^-tagged KAP1 (Fig. 6b). According to the previously described KAP1 role^39^, we confirmed that its depletion results in strongly reduced H3K9me3 levels at unique KAP1 targets determined based on our KAP1 ChIP-seq in XX mESCs (Supplementary Fig. 6b). We then analysed allelic H3K9me3 accumulation (Fig. 6c and Supplementary Fig. 6c), Xist RNA expression levels (Fig. 6d and Supplementary Fig. 6d), and X chromosome allelic silencing (Fig. 6e and Supplementary Fig. 6e) 24 h after Xist induction, both in the presence and absence of KAP1, and in several independent clones. We observed no discernible reduction in H3K9me3 deposition and no increase in Xist RNA levels nor acceleration of X-linked gene silencing. Levels of expression of the rtTA transgene were unaffected by KAP1 depletion (Supplementary Fig. 6f). These findings indicate that KAP1 does not contribute to SETDB1-mediated H3K9me3 nor downstream effects on Xist transcription and silencing, in spite of its increased association with the expressed allele.

**Fig. 7:**
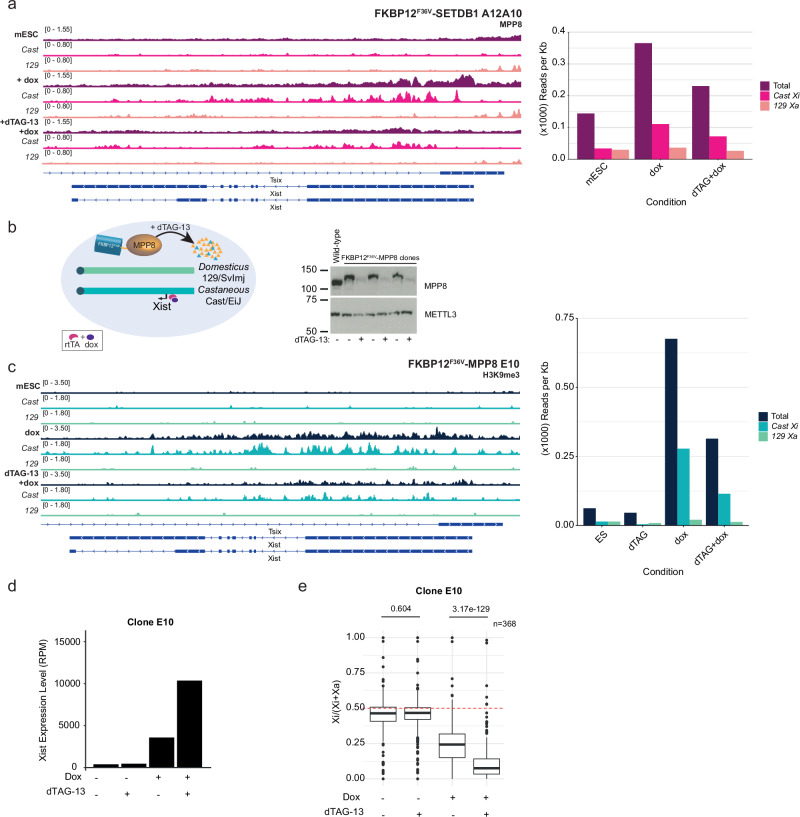
The HUSH complex is important for SETDB1-mediated effects on the transcribed Xist allele. **a** A representative snapshot of ChIP-seq analysis showing increased levels of MPP8 over the expressed Xist locus that are SETDB1 independent (compare + dox with + dTAG-13 + dox conditions), using FKBP12^F36V^-tagged SETDB1 mESCs (clone A12A10). Lighter shades represent reads assigned to Cast and 129 alleles using SNPsplit. Cast corresponds to the Xi in this cell line. Quantification of the total number of reads and reads assigned to Cast and 129 alleles is shown in the bar plot on the right. Source data are provided as a Source Data file. **b** Schematic illustrating Cast x 129 F1 mESC model engineered to allow depletion of endogenous MPP8 using dTAG-13. Western blot shows protein depletion after 2 h of dTAG-13 treatment. METTL3 is a loading control. Molecular markers indicated in kDa. Source data are provided as a Source Data file. **c** Representative cChIP-seq snapshot showing H3K9me3 over the Xist locus is reduced after MPP8 depletion in FKBP12^F36V^-tagged MPP8 mESCs (clone E10) (compare + dox with + dTAG-13 + dox conditions). Lighter shades represent reads assigned to each Cast and 129 alleles using SNPsplit. Cast corresponds to the Xi in this cell line. Quantification of the total number of reads and reads assigned to Cast and 129 alleles is shown in the bar plot (right). Source data are provided as a Source Data file. **d** Bar plot showing increased Xist RNA levels (RPM, reads per million mapped reads) following MPP8 depletion determined from ChrRNA-seq analysis of a FKBP12^F36V^-tagged MPP8 mESCs (clone E10) in conditions indicated at the bottom. Source data are provided as a Source Data file. **e** Boxplot of the allelic ratio (ranging from 0 to 1) of X-linked genes (*n* = 368) showing accelerated silencing following MPP8 depletion as determined by ChrRNA-seq analysis of a FKBP12^F36V^-tagged MPP8 mESCs (clone E10) in conditions indicated at the bottom. The red dashed line indicates an allelic ratio of 0.5. *P*-values were calculated using a two-sided paired *t* test. In box plots, centre lines indicate the median, box limits indicate the first and third quartiles, and whiskers indicate 1.5 × the interquartile range (IQR). Source data are provided as a Source Data file.

We next analysed the role of HUSH. Allelic ChIP-seq analysis of MPP8, a core subunit of HUSH, in XX mESCs with FKBP12^F36V^-tagged SETDB1 revealed association of the protein across the expressed Xist allele, although, unlike in the case of KAP1, allelic association was retained following SETDB1 depletion (Fig. 7a and Supplementary Fig. 7a). This observation suggests that MPP8 (HUSH) functions upstream of SETDB1 in the context of the Xist gene and may contribute to its recruitment. Transcription-linked MPP8 enrichment was also observed across L1 elements that are upregulated following SETDB1 depletion (Supplementary Fig. 6b). This observation is consistent with recent studies demonstrating nascent RNA-dependent HUSH recruitment at expressed TE loci^43,45^.

To directly test the contribution of HUSH to H3K9me3 deposition across the expressed Xist allele, we derived XX mESCs with FKBP12^F36V^-tagged MPP8 (Fig. 7b). cChIP-seq analysis of H3K9me3 following MPP8 depletion and Xist induction revealed a clear reduction, although not complete loss of allelic H3K9me3 over the expressed Xist allele (Fig. 7c and Supplementary Fig. 7c). Moreover, ChrRNA-seq analysis demonstrated markedly increased steady-state levels of Xist RNA (Fig. 7d and Supplementary Fig. 7d), and accelerated Xi gene silencing in mESCs (Fig. 7e and Supplementary Fig. 7e). MPP8 depletion had no discernible effect on expression levels of the rtTA transgene (Supplementary Fig. 7f). Similar results were obtained in an XX mESC lines with FKBP12^F36V^ tagging of a second HUSH subunit, TASOR (Supplementary Fig. 8a–f) suggesting a specific role for HUSH in the context of Xist regulation. We conclude that, unlike KAP1, the HUSH complex has a key role in transcription-dependent SETDB1 recruitment and H3K9me3 deposition over Xist.

## Discussion

Our results demonstrate that SETDB1 has a dual function in XCI, modulating Xist RNA abundance at the onset of XCI and contributing to long-term maintenance of silencing of X-linked genes. In relation to the XCI maintenance function, our results are in line with prior work^21,22^. The underlying mechanism for SETDB1 function in this context remains unclear but our analyses found that the modest de-repression of Xi genes in SETDB1 depleted long-term NPC cultures is biased towards genes for which completion of silencing is dependent on SMCHD1^46^. This subset of genes is silenced at a relatively slow rate during XCI in wild-type cells^46^. In support of this link, a recent study reported a role for SMCHD1 in H3K9me3 spread across Xi^47^. In this context, it is interesting to note that both SETDB1 and SMCHD1 have been implicated in regulating the protocadherin gene cluster^48–50^. In addition SMCHD1 contributes to higher-order chromatin architecture on Xi, and it will be interesting to determine if this feature is also affected by SETDB1 depletion.

Our finding that RNA-dependent recruitment of SETDB1 has a function in regulating Xist transcription was unanticipated. We note that SETDB1 was identified as a candidate Xist repressor in a recently described genetic screen using an independent cell model^51^, although whether or not this is linked to transcription-dependent SETDB1 recruitment or an alternative function in regulating Xist expression at the onset of XCI remains to be determined. Importantly, our analysis using TsixStop mutant mESCs indicates that Xist transcripts from the native promoter elicit the same pathway as the synthetic doxycycline-inducible promoter, albeit direct involvement of SETDB1 was not tested in this context. In both the inducible and native Xist promoter models the function of H3K9me3 over the expressed Xist allele appears to be limited to the first few days of (NPC) differentiation, as indicated by diminution of asymmetric H3K9me3 at a later timepoint (NPC d7). In light of recent evidence for a transcription-dependent mechanism for HUSH recruitment, our finding that HUSH contributes to SETDB1 recruitment over Xist helps to explain why this mechanism is triggered only on the expressed Xist allele. Specifically, the HUSH subunit Periphilin has been shown to mediate interaction with intronless adenine-rich RNAs arising from retroelements, pseudogenes, and transgene integrations, as well as genes with long exons^43^.

The pattern of MPP8 and H3K9me3 enrichment across the Xist locus, which includes intron sequences, suggests that HUSH recruitment is mediated by nascent Xist RNA molecules. It is interesting to note that Xist has an unusual gene structure with very large first and last exons ( ~ 9 and 6 kb, respectively), and as such may meet the requirements for RNA-dependent HUSH recruitment as occurs, for example, at long contiguous RNAs transcribed from transposons or at large intronless genes^43^. Whilst HUSH and SETDB1 depletion result in a similar increase in Xi silencing rates, HUSH depletion does not fully recapitulate the loss of allelic H3K9me3 observed in the absence of SETDB1, indicating the existence of a secondary SETDB1 recruitment pathway(s). Our findings effectively rule out a role for KAP1-mediated recruitment of SETDB1. Moreover, the fact that we observe similar effects depleting the HUSH subunits MPP8 and TASOR suggests that the HUSH2 complex, which is assembled around the orthologous TASOR2 protein^52,53^, is also not involved. Thus, further studies are required to identify the putative secondary pathway for SETDB1 recruitment to the expressed Xist allele.

Given that H3K9me3 deposition underlies SETDB1 function in gene repression^35,36^, we suggest that this histone PTM underpins suppression of Xist RNA transcription revealed through SETDB1/HUSH depletion. We cannot rule out a role for other factors, for example, the GHKL-ATPase MORC2, a subunit of the HUSH complex that is implicated in chromatin compaction. Our analyses suggest that Xist dampening occurs both at the level of transcription initiation and elongation. An explanation for the former effect may lie in the fact that Xist has an internal enhancer/promoter element located approximately 1 kb into the large first exon. For this to be relevant, it would be necessary to invoke that inducible Xist expression driven by the TetOn promoter is also dependent, at least to a degree, on the exon I enhancer. In this context, it is interesting to note that LINE1 elements, transcription of which is driven by an internal promoter^54,55^, are also targeted by HUSH^56–58^, and in this work were found to be prominently upregulated following SETDB1 depletion. These observations highlight how RNA-dependent recruitment of HUSH can be an important mechanism for repressing genes and invasive DNA elements which have regulatory sequences located within the transcription unit. This novel gene regulation pathway is apparently distinct from that occurring at immediate early genes, where H3K9me3-mediated regulation of transcriptional elongation has been linked to a KAP1-dependent pathway^59,60^.

The normal physiological function of the SETDB1/HUSH pathway in modulating Xist transcription is an open question. Prior work has demonstrated that there are feedback mechanisms that balance Xist transcription and turnover rates in different circumstances^9,10,29^, and we speculate that modulation via SETDB1 represents an important part of this mechanism. As noted above, Xist overexpression can result in overspill from the X chromosome onto neighbouring autosomes^7,8^, and it is likely this is occurring to a significant degree in our experiments, depleting SETDB1/HUSH. Circumstances in which feedback control of Xist expression is likely to be important include cell cycle regulation, where there is a rapid recoating of Xi by Xist RNA in early G1 and then maintenance of steady state levels for the remainder of the cell cycle up until mitosis^4^, and also during cellular differentiation where rapidly dividing progenitor cells give rise to slow dividing or quiescent differentiated derivatives. Here, it is worth noting that the modulation of Xist transcription by SETDB1 doesn’t appear to function after 7 days of NPC differentiation, indicating that alternative mechanisms may be required to maintain Xist levels in more fully differentiated cells.

In conclusion, this study highlights a previously unappreciated role for SETDB1/HUSH in controlling Xist RNA expression, providing a window into understanding autoregulation and feedback control in maintaining appropriate levels of Xist RNA to silence a single X chromosome.

## Methods

### Mouse embryonic stem cell lines and Drosophila S2 cells

Female mouse embryonic stem cells (mESC) used in this study were iXist-ChrX^Cast^ and iXist-ChrX^129^ (both derived from the parental F1 2-1 cell line)^27^, TsixStop and F1 2-1^37,38^. mESC were grown in ESC medium at 37 ^o^C in a 5% CO_2_ incubator and passaged onto 0.1% gelatin-coated plates in feeder-dependent conditions every 2-3 days using TrypLE Express (Life Technologies). Mitomycin C-inactivated SNLP (STO mouse fibroblasts expressing Neomycin and Puromycin resistance and *Lif* genes) were used as feeder cells. Cells were pre-plated and passaged at least twice to remove feeders prior to cell collection for genomic experiments.

ESC medium consisted of Dulbecco’s Modified Eagle Medium (DMEM; Life Technologies) supplemented with 10% Fetal Bovine Serum (FBS), 1x NEAA (Non-essential amino acids; Life Technologies), 2 mM L-glutamine (Life Technologies), 1x Penicillin-Streptomycin (Life Technologies), 50 µM b-mercaptoethanol (Life Technologies) and Leukemia Inhibitory Factor (LIF)-conditioned medium made in-house. To induce Xist expression in iXist-ChrX^Cast^ and iXist-ChrX^129^ cells lines, medium was supplemented with 1 µg/mL doxycycline. To induce protein degradation in lines expressing FKBP12^F36V^-tagged SETDB1, MPP8, TASOR or KAP1, medium was supplemented with 100 nM dTAG-13 (Tocris Bioscience). All cell lines were routinely tested for mycoplasma infection.

Drosophila S2 (Sg4) cells for calibrated ChIP-seq (cChIP-seq) experiments were grown semi-adherent at 25 ^o^C in Schneider’s Drosophila medium (Life Technologies) supplemented with 10% heat-inactivated Fetal Bovine Serum and 1x Penicillin-Streptomycin (Life Technologies).

### Molecular cloning of sgRNA sequences and donor vectors

Single guide RNAs (sgRNAs) to target Cas9 to defined genomic sequences (see Table 1) were designed using the CRISPOR online tool^61^ and ordered as custom oligos (Invitrogen) ready for hybridisation and cloning into pSpCas9(BB)−2A-Puro (PX459) V2.0. pSpCas9(BB)−2A-Puro (PX459) V2.0 was a gift from Feng Zhang (Addgene plasmid # 62988; http://n2t.net/addgene:62988; RRID:Addgene_62988). Cloning of sgRNA was performed using the single-step digestion-ligation Zhang lab protocol (Broad Institute). Donor vectors for CRISPR-assisted homologous recombination to target FKBP12^F36V^ to the end of each protein of interest (N-terminus in the case of SETDB1, MPP8 and TASOR and both N- and C- termini in the case of KAP1), were assembled using Gibson Assembly Master Mix (New England Biolabs). Homology arms ranging from 600 to 1000 bp (depending on the target) were PCR amplified from mESC genomic DNA using FastStart High Fidelity Polymerase (Merck Life Science). FKBP12^F36V^ fragment was originally amplified from pLEX_305-N-dTAG^62^. pLEX_305-N-dTAG was a gift from James Bradner & Behnam Nabet (Addgene plasmid # 91797; http://n2t.net/addgene:91797; RRID:Addgene_91797).

**Table 1:** Sequences of oligos for annealing and cloning of gRNAs into pSpCas9(BB)-2A-Puro (PX459) V2.0 plasmid, including plasmid overhangs necessary for cloning (in bold) and an extra G included when necessary, at the 5’ end to allow U6 promoter-driven expression of the gRNA (in italic)

Guide RNA ID	gRNA Sequence
[MA299]sgRNA_Setdb1_4(Score 46)_F	CACCGACATGCTTTGGCCCTGTGC
[MA300]sgRNA_Setdb1_4(Score 46)_R	**AAAC**GCACAGGGCCAAAGCATGTC
[MA303]Fam208a_sgRNA1(Score96)_F	**CACC***G*CCGCGCGATGGCGACTGCCG
[MA304]Fam208a_sgRNA1(Score96)_R	**AAAC**CGGCAGTCGCCATCGCGCGG*C*
[MA305]Trim28_sgRNA1 (Score 96)_F	**CACC***G*CCCCCCCGGCGGCGTGTGAA
[MA306]Trim28_sgRNA1 (Score 96)_R	**AAAC**TTCACACGCCGCCGGGGGGG*C*
[MA365]Trim28_sgRNA2 (Score 69)_F	**CACC***G*CAGTGCTGGCCTAAGTTCTC
[MA366]Trim28_sgRNA2 (Score 69)_R	**AAAC**GAGAACTTAGGCCAGCACTG*C*
[MA403]MPP8_sgRNA1 (Score99)_F	**CACC***G*TCGCCGGCGGTTTTTCGCGT
[MA404]MPP8_sgRNA1 (Score99)_R	**AAAC**ACGCGAAAAACCGCCGGCGA*C*

Donor vectors were designed containing a mutant PAM sequence to prevent Cas9 DNA cleavage. Cloned vectors were transformed into XL10-Gold ultracompetent bacteria (Agilent) and plated to grow overnight with ampicillin selection. DNA was isolated from bacterial colonies using the Plasmid Miniprep kit (Qiagen), screened for containing the desired plasmid, and their sequences were confirmed via Sanger sequencing (Source Biosciences).

### Genome editing for endogenous knock-in of FKBP12^F36V^

To generate mESC in which depletion of SETDB1, MPP8, TASOR and KAP1 can be rapidly induced by addition of dTAG-13, FKBP12^F36V^ was knocked-in into the N-terminus of the first 3 and N-terminus and C-terminus of KAP1 (one tag per allele). To do this, 1.5 × 10^6^ iXist-ChrX^Cast^ mESC were plated on feeders per well of a 6 well plate a day before transfection. Targeting vectors containing the FKBP12^F36V^ sequence in between ~600-1000 bp homology arms were co-transfected in a 6:1 molar ratio with plasmid pSpCas9(BB)−2A-Puro (PX459) V2.0 (Addgene #62988), containing the corresponding sgRNA, using Lipofectamine 2000 (Invitrogen) according to the manufacturer’s instructions. The day after transfection, transfected cells were split at 3 different densities into 10 cm Petri dishes containing feeder cells to allow them to attach before being exposed to 4.5 µg/mL puromycin selection for 48 h. After this period, cells were fed with regular mESC medium for 10-12 days until colonies were visible and ready to pick into 96-well plates with feeders. This plate was then split in triplicate (2 plates without feeder cells), from which one was used to lyse cells for protein extract for screening of targeted clones by Western blot and a second one was used to isolate DNA for PCR screening when Western blot was not a viable option. Targeted clones were further characterised by PCR of genomic DNA (see primers in Table 2) and Sanger sequencing to confirm in-frame knock-in of FKBP12^F36V^ and homozygosity. The efficiency of depletion using dTAG-13 was determined by Western blot.

**Table 2:** Primers used for screening and characterisation of knocked-in clones

Primer ID	Primer Sequence
[MA351] Setdb1_5’HA_F	CCCGGGAAATCTCCAGATCG
[MA352] Setdb1_Intron2_R	CCAGCATGGCCTACAGAGTA
[MA353] FKBP_F	gtggaaaccatctccccaggag
[MA356] FKBP_R	ttccagttttagaagctccacatcgaagac
[MA357] Fam208a_5’HA_F	TTAAGAGGCGTCTCTGTGGC
[MA358] Fam208a_Intron1_R	AGTAAGCAACCCAACTGTTCCT
[MA359] Trim28_5’HA_F	TTGGCCGCATCTCGACAG
[MA360] Trim28_Intron2_R	AGGCTCTGGAAAAGGTCACG
[MA410] Trim28_Intron 15_F	acaggagaggttgcaggtg
[MA411] TRIM28_3’HA_R	CAGAGCTGGACTGGGCTG
[MA412] TRIM28_3’HA_R	CAGAGCTGGACTGGGCTG
[MA413]Setdb1_specific_R	ACTTCAATTTTCCACCTGCTGTTACAAAAG
[MA414]Duplication_specific_R	AGTCAAGATCATGGAGCAAATTCAAGG
[MA424]MPP8_5’UTR_F	ctctcgctatgtatgcccttggaa
[MA425]MPP8_Intron1_R	aagggacactttgggttggg
[MA426]FKBP_F2	catgccactctcgtcttcgatgtg
[MA427]Fam208a_5’UTR_F	tgctcgtgatctactgggaaagc
[MA428]FKBP_R2	aaggtgcgcccgtctcctg

### Neural Progenitor Cell (NPC) differentiation

Mouse embryonic stem cells were differentiated into NPC as previously described^63,64^. To obtain feederless mESC cell suspensions, mESC were collected using TrypLE and pre-plated four consecutive times for 40 min each. Cells were then washed in PBS twice to remove any traces of FBS and counted using a Countess® II FL Automated Cell Counter (Life Technologies). Cells were then resuspended in N2B27 medium (50% Neurobasal; 50% DMEM/F12 with 0.5 x N2 and 0.25 x B27, 1mM L-glutamine, 0.5x Penicillin-Streptomycin, 100 μM b-mercaptoethanol, all from Life Technologies) supplemented with 1 μg/mL of doxycycline to induce Xist expression simultaneously with the onset of differentiation, and plated at a density of 20,000 cells per cm^2^ in pre-gelatinised plates. Cells were fed daily from day 2 to day 7 of differentiation and fed also on day 1 for experiments in which this corresponded to the timepoint of 100 nM dTAG-13 addition. dTAG-13 treated NPC cultures were fed with media containing dTAG-13 from NPCd1 onwards until collection. On day 7, cells were dissociated by incubation with accutase (diluted 1:3 in PBS) (Merck Life Sciences) and counted. Three million cells were resuspended in N2B27 supplemented with doxycycline and 10 ng/mL EGF (cat# 315-09, Peprotech) and 10 ng/mL FGF2 (cat# 100-18B, Peprotech) and plated to grow in suspension in non-adherent bacterial petri dishes to promote embryoid body formation. On day 10, cells were recovered by centrifugation and plated onto pre-gelatinised dishes to expand more homogeneous neural stem cell cultures. At this point, neural stem cells were fed every other day and passaged as required using accutase (diluted 1:3 in PBS) (Merck Life Sciences) until the required timepoint. Cells were collected for different experiments at the appropriate timepoints (generally, NPCd1, d3 and d7 for ChIP experiments and multiple timepoints from NPCd1 to NPCd28 for chromatin-RNA) by washing with PBS and then incubating the cells at room temperature with accutase (diluted 1:3 in PBS) (Merck Life Sciences) until they were dissociated for collection.

### Preparation of total protein lysates and nuclear protein extracts

For mESC colony screening, clones grown in 96 well plates without feeders were washed once in PBS and lysed immediately after PBS removal in Laemmli buffer (0.2 M Tris pH 6.8, 2% SDS, 20% glycerol, 10% β-mercaptoethanol, 0.2% bromophenol blue) with agitation at room temperature for 20 min before snap freezing and storage at − 20 ^o^C until further use. Total cell lysates were then thawed and transfer into a PCR plate and boiled at 95 ^o^C for 10 min immediately before loading on a SDS-PAGE for Western blot.

To confirm dTAG-13 treatment efficacy in depleting FKBP12^F36V^ tagged proteins, cells were grown in 10 cm Petri dishes and a plate was treated using 100 nM dTAG-13 for 2 h before cell collection by trypsinization and pelleting after a PBS wash. To prepare nuclear protein extracts, these pellets were then resuspended in hypotonic Buffer A (10 mM HEPES pH 7.9, 1.5 mM MgCl2, 10 mM KCl, 0.5 mM DTT, complete protease inhibitors) and incubated on ice for 10 min before recovery by centrifugation. Cells were then lysed using Buffer A containing 0.1% NP40 and incubated on ice for 10 min. Nuclei were briefly washed with PBS, pelleted and then resuspended in Buffer C (5 mM HEPES, pH 7.9, 26% glycerol, 1.5 mM MgCl_2_, 0.2 mM EDTA, 350 mM NaCl, 0.5 mM DTT, complete protease inhibitors) for nuclear protein salt extraction by incubating them on ice for 1 h. The nuclei were spun at 13000 rpm for 20 min at 4 ^o^C and the supertatant was collected as nuclear protein extract. These extracts were then quantified using the Bradford assay (Bio-Rad) according to the manufacturer’s instructions.

### Western blot

Protein extracts were resolved on an SDS-PAGE separating gel with a stacking gel alongside Precision Plus Protein Dual Colour Standards (Bio-Rad, Cat #1610374). After gel separation, proteins were transferred to a 0.2 µm nitrocellulose membrane using the High MW or Mixed MW programmes on a Trans-Blot Turbo Transfer System (Bio-Rad). Membranes were then blocked for at least 1 h in TBST (100 mM Tris pH7.5, 0.9% NaCl, 0.1% Tween) with 5% (w/v) non-fat milk. Membranes were incubated with antibodies diluted in TBST with 5% (w/v) non-fat milk overnight at 4 ^o^C with agitation. Antibodies used for Western blot are listed in Table 3. Membranes were then washed 3 times for at least 10 min each in TBST with 5% non-fat milk and then incubated with secondary antibodies for 1 h at room temperature with agitation. Secondary antibodies (horseradish peroxidase-conjugated antibodies -1:2000) were diluted in TBST with 5% non-fat milk. After incubation, membranes were washed twice with TBST (100 mM Tris pH 7.5, 0.9% NaCl, 0.1% Tween) and once with PBS, 15 min each with agitation. Membranes were then incubated for 5 min with Clarity Western ECL Substrate (Bio-Rad, Cat #1705061), and signal was developed onto Amersham Hyperfilm ECL (GE Healthcare). Uncropped scans are provided as Source Data.

**Table 3:** Antibodies used in this study

Reagent	Source	Identifier
Rabbit polyclonal anti-H3K9me3	Abcam	Cat # ab8898; RRID:AB_306848
Mouse monoclonal anti-H3K9me3	Active Motif	Cat #061013; RRID:AB_2687870
Rabbit polyclonal anti-SETDB1	Proteintech	Cat# 11231-1-AP; RRID:AB_2186069
Rabbit polyclonal anti-MPP8	Proteintech	Cat# 16796-1-AP, RRID:AB_2266644
Rabbit polyclonal anti-KAP1	Abcam	Cat# ab10484, RRID:AB_297223
Mouse monoclonal anti-KAP1	Abcam	Cat# ab22553, RRID:AB_447151
Rabbit polyclonal anti-FAM208A	Novus Biologicals	Cat# NBP1-90673, RRID:AB_11006471
Rabbit monoclonal anti-RBP1 NTD (D8L4Y)	Cell Signalling Technology	Cat# 14958, RRID:AB_2687876
Rabbit monoclonal anti-phospho-RBP1 CTD (Ser5) (D9N5I)	Cell Signalling Technology	Cat# 13523, RRID:AB_2798246
Rabbit monoclonal anti-phospho-Rpb1 CTD (Ser2) (E1Z3G)	Cell Signalling Technology	Cat# 13499, RRID:AB_2798238
Rabbit monoclonal anti-METTL3	Abcam	Cat # ab195352, RRID:AB_2721254
Donkey Polyclonal Anti-Rabbit IgG, Whole Ab ECL Antibody, HRP Conjugated	Cytiva	Cat# NA934, RRID:AB_772206

### RNA-FISH (including probe preparation)

FKBP12^F36V^-SETDB1 mESCs were grown on gelatin-coated 22 × 22 mm No. 1.5H precision coverslips (170 ± 5 µm; Marienfeld Superior) in 6-well plates. Cells were treated with 100 nM dTAG-13 for 3 h followed by 24 h of Xist induction with 1 µg/ml doxycycline. Following treatment, coverslips were washed with PBS, fixed with 3.7% formaldehyde in PBS for 10 min at room temperature. Cells were rinsed three times with PBST.5 (PBS + Tween20 0.05%), permeabilized with 0.2% Triton X-100 in PBS for 10 min, washed again three times with PBST.5, followed by a 30 min incubation in 70% ethanol at room temperature. Coverslips were then dehydrated with subsequent washes with 80, 90 and 100% ethanol for 5 min each and briefly dried.

Xist probes were generated from a plasmid containing the 18 kb cDNA of Xist using a nick translation kit (Abbott) and SEEBRIGHT^®^ Green dUTP (Enzo) following the manufacturers protocol with incubation for 16 h at 15 ^o^C. 3 µl of Green labelled Xist probes per hybridisation were ethanol precipitated with 1/3 volume of 10 mg/ml salmon sperm DNA, 1/10 volume of 3 M NaOAc and 3 volumes of 100% ethanol. After spinning and washing with 70% ethanol, the pellet was air dried and resuspended in deionized formamide (Sigma). Probes resuspended in formamide were added to the hybridisation mixture to a final concentration of 2 x SSC buffer, 50% Formamide (containing probes), 5% Dextran sulphate, 1 mg/ml BSA, 2 mM Vanadyl Ribonucleoside Complex.

Cells on coverslips were hybridised with 30 µl probe/hybridisation buffer mix that was denatured at 75 °C for 5 min before briefly being chilled on ice. Hybridisation was carried out in a humid chamber overnight at 37 °C. The next day, coverslips were washed three times with prewarmed 50% formamide/2x SSC at 42 °C for 5 min each, and subsequently three times with 2x SSC at 42 °C for 5 min each. Coverslips were rinsed with water to remove excess salts and mounted with Vectashield antifade mounting medium containing DAPI (Vector Labs) on Superfrost Plus microscopy slides (VWR). Slides were dried, sealed using clear nail polish, and cleaned for imaging.

### Image acquisition and analysis

Images were acquired using the VisiTech iSIM super-resolution array scanning module built around an Olympus IX83 inverted microscope. A 100X objective (UPLAPO100XOHR - NA = 1.5, Olympus) was used along with two solid-state lasers (405 nm and 488 nm – both 10 mW) with illumination at 80% power and an exposure time of 400 ms. Images were captured on a Hamamatsu Orca Quest camera.

Images were analysed by cropping individual nuclei as 3D stacks in ImageJ before being saved as separate tiff files. Xist foci xyz co-ordinates were then computed for each nucleus separately using the TrackMate Batcher plugin^65^ and exporting the ‘all-spots.csv’ files. Foci were detected using the DoG detector, a quality score ≥ 20, a diameter of 200 nm and a contrast ≥ 0.045 to reduce false positives from the overlap detected from foci in close proximity within the Xist cloud. The csv files containing foci coordinates for each nucleus were then imported as a ‘cell array’ in MATLAB so that volume and distance measurements could be computed independently. Using Matlab, the volumes of Xist clouds were calculated from Xist foci xyz coordinates using a 3D convex hull calculation. Unique nearest neighbour distances were calculated using the knnsearch function that calculated Euclidean distances between neighbouring foci, and the total number of foci was extracted from each cell array. Data was plotted with Igor Pro 9 (WaveMetrics) and either depicted as box plots or violin plots and an unpaired single tailed students *T* test was used for statistical analysis between individual datasets of two biological replicates to analyse significant differences within the populations on a single-cell level.

### Chromatin immunoprecipitation and sequencing (ChIP-seq)

Chromatin immunoprecipitation was performed as previously described^46,66^. Calibrated native ChIP-seq (cChIP-seq) was performed for H3K9me3 by collecting 5 × 10^7^ mESCs or NPCs and spiking in 1 × 10^7^ Drosophila Sg4 cells, counted using a Countess II FL Automated Cell Counter (Life Technologies). Cells were pelleted by centrifugation and resuspended in 1 mL of cold RSB (10 mM Tris pH 8.0, 10 mM NaCl, 3 mM MgCl2) and lysed by the addition of 14 mL of cold RSB supplemented with 0.1% NP40 and gentle inversion. Nuclei were recovered by centrifugation at 1500 x *g* for 5 min at 4 ^o^C and washed in 4 ml RSB with 0.25 M sucrose and 3 mM CaCl_2_ supplemented with protease inhibitors. Nuclei were recovered by centrifugation at 1500 x *g* for 5 min at 4 ^o^C and resuspended in 1 mL of RSB with 0.25 M sucrose and 3 mM CaCl_2_ supplemented with protease inhibitors, which was transferred to a 1.5 mL tube for MNase digestion (200U, Fermentas EN0181) of chromatin to mono- and di-nucleosomes at 37 ^o^C for 5 min with inversion every minute. The reaction was quenched by the addition of 8 µL 0.5 M EDTA and spun at 5000 rpm for 5 min at 4 ^o^C. The supernatant (S1) was collected into a new 1.5 mL tube and kept on ice. The pellet was thoroughly resuspended in 300 µL of Nucleosome Release Buffer (10 mM Tris pH 7.5, 10 mM NaCl, 0.2 mM EDTA) supplemented with protease inhibitors, and incubated for 1 hour at 4 ^o^C with rotation. This suspension was passed five times through a 27 G needle using a 1 mL syringe and then spun at 5000 rpm for 5 min at 4 ^o^C. The supernatant (S2) was collected and combined with S1. The chromatin was centrifuged at 13000 rpm for 5 min to remove any precipitant, and the supernatant was collected as chromatin, snap frozen and stored at − 80 ^o^C until further processing. For immunoprecipitation (IP), chromatin was thawed on ice, and 110 µL were diluted in 1 mL of native ChIP incubation buffer (70 mM NaCl, 10 mM Tris pH 7.5, 2 mM MgCl2, 2 mM EDTA, 0.1% Triton X-100) freshly supplemented with protease inhibitors. 100 µL were transferred to a new 1.5 mL tube and stored at 4 ^o^C to be used as input. The remaining diluted chromatin was incubated overnight with rotation with 5 µg of H3K9me3 antibody (Abcam, ab8898) or H3K9me3 (Active Motif; AB2532132 Clone 0319; Cat #061013). Samples were then incubated for 90 min with 45 µL rProtein A Sepharose™ Fast Flow beads (Amersham) pre-blocked with 1 mg/mL of yeast tRNA and 1 mg/mL BSA. The beads were then washed five times for 10 min with rotation at 4 ^o^C with Wash Buffer (20 mM Tris pH 7.5, 2 mM EDTA, 125 mM NaCl, 0.1% Triton X-100) followed by one final wash in 1x TE. The DNA was then eluted from the beads by incubation at 25 ^o^C for 30 min shaking at 1000 rpm with 1% SDS, 100 mM NaHCO_3_ elution buffer and separated from the beads by centrifugation. DNA from the IP elutions and input samples was further purified using the ChIP DNA Clean and Concentrator Kit (Zymo Research) according to the manufacturer’s instructions. ChIP and input DNA was quantified using the Qubit High Sensitivity DNA kit (Invitrogen) and submitted for library preparation using the NEBNext Ultra II DNA Library Prep Kit (New England Biolabs) according to the manufacturer’s instructions. Libraries were quantified using the Qubit High Sensitivity DNA kit (Invitrogen), and their length was estimated using the Bioanalyzer High Sensitivity DNA kit (Agilent) before pooling and paired-end sequencing on an Illumina NextSeq 500 machine using NextSeq 500/550 High-Output v2.5 Kit (150 cycles) (Illumina).

RNA Polymerase II calibrated ChIP-seq (cChIP-seq) was performed as previously described^66,67^. For RNA polymerase II calibrated ChIP-seq (cChIP-seq), 5 × 10^7^ mESCs were pooled with 1 × 10^7^ Drosophila cells after collection, washed in PBS wash and counted using a Countess II FL Automated Cell Counter (Life Technologies). Cells were crosslinked for 10 min with 1% formaldehyde. The reaction was quenched using glycine for an additional 10 min before recovering the cells by centrifugation at 1000 × *g* for 5 min at 4 ^o^C. After a PBS wash, supernatant was removed and cells were snap frozen and stored at − 80 ^o^C until use.

Cell pellets were thawed on ice, resuspended in 1 mL of FA-Lysis Buffer (50 mM HEPES pH7.9, 150 mM NaCl, 2 mM EDTA, 0.5 mM EGTA, 0.5% NP-40, 0.1% Sodium deoxycholate, 0.1% SDS) supplemented with protease inhibitors, 1 mM AEBSF, 10 mM NaF and incubated on ice for 10 min prior to sonication using a Bioruptor Pico Sonicator (Diagenode) for 30 cycles (30 sec on/off). Post-sonication chromatin was spun at maximum speed for 10 min at 4 ^o^C and the supernatant was transferred to a new 1.5 mL tube. For each IP, 300 µg of chromatin were diluted to a total volume of 1 mL in FA-Lysis Buffer and precleared by incubation with 40 µL of pre-blocked rProtein A Sepharose™ Fast Flow bead slurry (Amersham) for 1 h at 4 ^o^C with rotation. Pre-cleared chromatin was aliquoted in 1.5 mL Protein LoBind tubes and incubated overnight at 4 ^o^C with rotation with the corresponding antibody (15 µL Anti-Rbp1 NTD, D8L4Y, Cell Signalling Technologies; 12.5 µL Anti-Rbp1 Ser5P, D9N5I, Cell Signalling Technologies; 12.5 µL Anti-Rbp1 Ser2P, E1Z3G, Cell Signalling Technologies). 10% of pre-cleared chromatin was kept as input. After incubation, 40 µL of pre-blocked rProtein A Sepharose™ Fast Flow bead slurry (Amersham) were added to each sample and incubated for a minimum of 3 h at 4 ^o^C followed by single 5 min washes at 4 ^o^C with FA-Lysis, FA-Lysis 500 (same composition as FA-Lysis Buffer but with 500 mM NaCl) and DOC (10 mM Tris pH8.0, 250 mM LiCl, 2 mM EDTA, 0.5% NP-40, 0.5% Sodium deoxycholate) buffers and two washes with TE pH8.0. The DNA was then eluted from the beads by incubation at 30 ^o^C for 30 min shaking at 1000 rpm with 1% SDS, 100 mM NaHCO_3_ elution buffer and separated from the beads by centrifugation. IP and input samples were reverse crosslinked overnight and RNAse- and Proteinase K-treated, and the DNA was further purified using the ChIP DNA Clean and Concentrator Kit (Zymo Research) according to the manufacturer’s instructions. ChIP and input DNA was quantified using the Qubit High Sensitivity DNA kit (Invitrogen) and submitted to library preparation using the the NEBNext Ultra II DNA Library Prep Kit (New England Biolabs) according to the manufacturer’s instructions. Libraries were quantified using the Qubit High Sensitivity DNA kit (Invitrogen), and their length was estimated using the Bioanalyzer High Sensitivity DNA kit (Agilent) before pooling and subjected to paired-end sequencing on an Illumina NextSeq 500 machine using NextSeq 500/550 High-Output v2.5 Kit (150 cycles) (Illumina).

For KAP1 and MPP8 ChIP-seq, 5×10^7^ mESC cells were collected in PBS and double crosslinked by incubating with 2 mM DSG for 50 min at room temperature, followed by an additional 12 min with 1% formaldehyde. The reaction was quenched by the addition of glycine and further incubation with rotation for 10 min. Cells were pelleted by centrifugation, washed once with PBS, and the pellet was snap frozen and stored at − 80 ^o^C. To prepare chromatin, the cell pellets were thawed on ice and lysed in 10 mL of LB1 (50 mM HEPES-KOH pH 7.9, 140 mM NaCl, 1 mM EDTA, 10% Glycerol, 0.5% NP40, 0.25% Triton X-100) supplemented with protease inhibitors for 10 min, rotating at 4 ^o^C. Nuclei were recovered by centrifugation and resuspended in 10 mL LB2 (10 mM Tris pH 8.0, 200 mM NaCl, 1 mM EDTA, 0.5 mM EGTA) with protease inhibitors and incubated rotating for 10 min. After centrifugation, the pellet was resuspended in 1 mL of LB3 (10 mM Tris pH 8.0, 100 mM NaCl, 1 mM EDTA, 0.5 mM EGTA, 0.1% Sodium Deoxycholate, 0.5% N-lauroylsarcosine) with protease inhibitors per 5 × 107 cells and subjected to sonication using a Bioruptor Pico (Diagenode) for 30 cycles (30 sec on/off). After sonication, samples were spun for 2 min 2000rpm at 4 ^o^C and the supernatant was collected into a new tube to which 110 µL of LB3 with 10% Triton X-100 were added and mixed by inversion. Chromatin was spun at maximum speed for 10 min and the supernatant was collected into a new tube, snap frozen and stored. For immunoprecipitation, chromatin was thawed on ice, and 110 µL were taken per reaction and diluted in 990 µL ChIP dilution buffer (1% Triton X-100, 1 mM EDTA, 20 mM Tris pH 8.0, 150 mM NaCl). Chromatin was precleared by incubation with pre-blocked rProtein A Sepharose™ Fast Flow beads (Amersham) for 1 hour (40 μL bead slurry/mL of chromatin) at 4 ^o^C and 100 μL set aside as input. Antibodies (KAP1, Abcam, ab22553, 5 µg; MPP8, Proteintech, 16796-1-AP, 7 µg) were added to 1 mL of precleared chromatin each and incubated overnight at 4 ^o^C with rotation. Samples were then incubated for 4 h with 40 µL of pre-blocked rProtein A Sepharose™ Fast Flow beads (Amersham) followed by single 4 min washes with Low Salt (0.1% SDS, 1% Triton X-100, 2 mM EDTA, 20 mM Tris pH 8.0, 150 mM NaCl), High Salt (0.1% SDS, 1% Triton X-100, 2 mM EDTA, 20 mM Tris pH 8.0, 500 mM NaCl), LiCl (0.25 M LiCl, 1% NP-40, 1% Deoxycholate, 1 mM EDTA, 10 mM Tris pH 8.0) wash buffers and two washes with 1xTE. The DNA was then eluted from the beads by incubation at 25 ^o^C for 30 min shaking at 1000 rpm with 1% SDS, 100 mM NaHCO_3_ elution buffer and separated from the beads by centrifugation. IP and input samples were reverse crosslinked overnight and RNAse- and Proteinase K-treated, and the DNA was further purified using the ChIP DNA Clean and Concentrator Kit (Zymo Research) according to the manufacturer’s instructions. ChIP and input DNA was quantified using the Qubit High Sensitivity DNA kit (Invitrogen) and used for library preparation using the NEBNext Ultra II DNA Library Prep Kit with size selection post-PCR amplification (New England Biolabs) according to the manufacturer’s instructions. Libraries were quantified using the Qubit High Sensitivity DNA kit (Invitrogen). DNA length was estimated using the Bioanalyzer High Sensitivity DNA kit (Agilent) before pooling and using for paired-end sequencing on an Illumina NextSeq 500 machine using NextSeq 500/550 High-Output v2.5 Kit (150 cycles) (Illumina).

### ChIP-sequencing analysis

ChIP-sequencing analysis was performed using previously established pipelines^46^. For calibrated ChIP-seq experiments, raw fastq reads were mapped using Bowtie2 (v.2.3.5.1)^68^ to the 129S1xCast N-masked mm10 genome concatenated with the dm6 Drosophila genome using the following parameters: “--very-sensitive --no-discordant --no-mixed -X 2000”. Unmapped reads were then removed. PCR duplicates were identified and removed using picard-tools “MarkDuplicates” (Broad Institute) and the remaining reads were then assigned to each allele using SNPsplit^34^. To calculate calibration factors, mm10 and dm6 mapped reads for each sample and input were counted using SAMtools (v.1.16)^69^. Calibration factors for cChIP-seq were then calculated according to the derived formula for occupancy ratio (ORi)^70^. In order to generate calibrated bigwig files for mouse chrX, bam files were initially filtered using the “intersect” function from BEDtools (v.2.30.0)^71^ to generate a bam file containing mouse chrX reads only. During the generation of the iXist-ChrX^Cast^ cell line using CRISPR-Cas9–facilitated homology-directed recombination, two SNPs (mm10, chrX:103482682 A/T and mm10, chrX:103482895 A/T) between the Cast and 129 alleles became uninformative, as both X alleles were converted to the 129 allele (T at both positions). Consequently, any reads spanning these two sites are no longer allele-specific as intended and were therefore removed from the allelic BAM files. BAM files were then sorted and indexed using SAMtools^69^ and bigWig files were finally generated using deepTools (v.3.5.1)^72^ bamCoverage per 10 million reads considering the calibration factors. For KAP1 and MPP8 (non-calibrated), the ChIP signal was calculated from one million library size normalised values. BigWig files were then visualised using IGV^73^. To quantify the number of ChIP reads per kb over the Xist locus (chrX: 103460373-103483233), we used the function “bigWigAverageOverBed” from UCSCTools.

To determine RNA Polymerase II pausing index on the Xist locus from RNA Polymerase II cChIP-seq, we calculated the ratio of the read density within the promoter region (-50 to 300 bp relative to TSS) over the read density within the gene body (300 bp downstream of the TSS to TTS) using a custom Python script.

### Chromatin-RNA extraction and sequencing (ChrRNA-seq)

Chromatin-RNA isolation and sequencing was as previously described^16,74^. Briefly, cells were collected in a 15 mL tube, washed with PBS and snap frozen immediately after PBS removal. Cells pellets were stored at − 80 ^o^C until further processing. In order to purify chromatin-RNA, pellets were thawed on ice and resuspended in 800 mL of hypotonic buffer HLBN (10 mM NaCl, 2.5 mM MgCl_2_, 10 mM Tris pH 7.5, 0.1% NP40) and transferred to a 1.5 mL tube. This solution was underlaid with a 480 mL HLBN containing 24% sucrose to create a sucrose cushion for nuclear isolation by centrifugation at 1000 g for 5 min at 4 ^o^C. For fractionation, the nuclear pellet was then resuspended in 120 mL NUN1 (20 mM Tris pH8.0, 75 mM NaCl, 0.5 mM EDTA, 50% glycerol) freshly supplemented with 1 mM DTT and protease inhibitors. After resuspension, 1200 mL NUN2 (20 mM HEPES pH7.6, 7.5 mM MgCl_2_, 0.2 mM EDTA, 0.3 M NaCl, 1 M urea, 1% NP40) freshly supplemented with 1 mM DTT and protease inhibitors were added and mixed by gentle vortexing. The sample was incubated on ice for 15 min with occasional vortexing. After centrifugation for 10 min at 15000 rpm at 4 ^o^C, the supernatant containing the nucleoplasmic fraction was discarded, and the chromatin pellet was resuspended in 200 mL HSB (10 mM Tris pH7.5, 500 mM NaCl, 10 mM MgCl_2_) supplemented with 2 mL TURBO DNAse (Life Technologies) to digest the sample DNA by incubating at 37 ^o^C for at least 10 min, shaking at 1400 rpm. After this incubation, 2 μL of 20% SDS and 4 μL of proteinase K (20 mg/ml) were added, and the samples were incubated at 37 ^o^C for at least 30 min for protein digestion. Chromatin-bound RNA was then purified using Trizol reagent according to the manufacturer’s recommendations with addition of glycogen to aid precipitation of the RNA at − 20 ^o^C overnight. Purified chromatin-RNA was subjected to a second round of TURBO DNAse treatment and a second round of Trizol reagent purification as previously described. Chromatin-RNA concentrations were initially measured by Nanodrop to inform further dilution for quantification using Qubit HS RNA assay (Invitrogen) and quality control using RNA Pico 6000 Bioanalyser kit (Agilent). RNA Libraries were prepared for sequencing using the Illumina TruSeq stranded total RNA kit, including the rRNA depletion according to the manufacturer’s instructions. Libraries were quantified and verified using Qubit High Sensitivity DNA (Invitrogen) and the Bioanalyser High Sensitivity DNA (Agilent) kits before pooling and paired-end sequencing on an Illumina NextSeq 500 machine using NextSeq 500/550 High-Output v2.5 Kit (150 cycles) (Illumina).

### 4sU-labelled RNA isolation and sequencing (4sU-seq)

4sU labelling of RNA, purification and sequencing was as previously described with a few modifications^30,75^. For 4sU labelling of newly synthesised RNA, 4sU was added to the cell culture media at a final concentration of 500 mM, and cells on a nearly confluent 10 cm Petri dish were incubated for 20 minutes prior to quick removal of media and direct lysis using Trizol reagent (Life Technologies). Lysates were snap frozen and kept at − 80 ^o^C until further processing. RNA isolation was performed using Trizol-chloroform phase separation, followed by isopropanol precipitation of the RNA and a 75% ethanol wash prior to dissolving the RNA pellet. Purified RNA was treated using TURBO DNAse as per the manufacturer’s recommendation. RNA was then quantified using Nanodrop to prepare the Biotinylation reaction consisting of 7 mg MTSEA biotin-XX linker (Biotium, cat. no. BT90066) dissolved in DMF at a concentration of 0.1 mg/mL, and 3.5 μL of 10X Biotinylation buffer (100 mM Tris-HCl, pH 7.4, 10 mM EDTA) per μg of purified RNA. This reaction was incubated at room temperature in the dark for 30 min, prior to two rounds of RNA purification using phenol:chloroform:isoamylalcohol (25:24:1) and Phase Lock Gel Heavy tubes and subsequent isopropanol precipitation with NaCl. RNA was then denatured at 65 ^o^C for 10 min and immediately cooled on ice for 5 min. Biotinylated 4sU-labelled RNA was captured using 200 mL of μMACS streptavidin MicroBeads (from the μMACS Streptavidin Kit) and incubating for 15 minutes at room temperature prior to recovering using the μMacs Streptavidin Kit (Miltenyi). Samples were washed six times using Washing Buffer (100 mM Tris pH 7.5, 10 mM EDTA, 1 M NaCl, 0.1% Tween20), the first three at 65 ^o^C, before elution using fresh 100 mM DTT into RLT buffer from the RNeasy minElute kit, which was used for a final RNA cleanup. RNA was quantified, and its integrity was confirmed on Bioanalyser using RNA Pico 6000 Bioanalyser Kit (Agilent). Libraries were prepared for sequencing using the Illumina TruSeq stranded total RNA kit, including the rRNA depletion. Libraries were quantified and verified using Qubit High Sensitivity DNA (Invitrogen) and the Bioanalyser High Sensitivity DNA (Agilent) kits before pooling and paired-end sequencing on an Illumina NextSeq 500 machine using NextSeq 500/550 High-Output v2.5 Kit (150 cycles) (Illumina).

### Allelic analysis of ChrRNA-seq

The data processing pipeline for ChrRNA-seq followed procedures similar to those described by Nesterova et al^27^. Briefly, raw FASTQ files containing paired-end reads were first aligned to a custom rRNA reference using Bowtie2 (v2.3.5.1)^68^ to remove rRNA contaminants. Reads mapping to rRNA were discarded, and the remaining unmapped read pairs were subsequently aligned to the N-masked mouse genome (mm10) using STAR (v2.5.2b)^76^. STAR was run with the following parameters: “--twopassMode Basic --outSAMstrandField intronMotif --outFilterMismatchNoverReadLmax 0.06 --outFilterMultimapNmax 1 --alignEndsType EndToEnd”. Only uniquely mapped reads were retained for downstream analyses. To distinguish allelic origin, alignments were split into Cast and 129S alleles using SNPsplit (v0.4.0dev, Babraham Institute)^34^ with the “*--*paired” option, based on annotated SNPs present between the two strains. Allele-specific read counts were obtained using featureCounts (v1.5.2)^77^ with the parameters “-t transcript -g gene_id -s 2”. Alignments were sorted using SAMtools (v1.16)^69^. For data visualisation, BEDtools (v2.30)^71^ was used to generate bigWig coverage tracks, which were then visualised using IGV^73^. Metagene profiles and heatmaps were created using deepTools (v3.5.2)^72^, supplemented with custom Python scripts. For biallelic (non-split) analyses, read counts were normalised to counts per million (CPM) mapped read pairs using the edgeR R package (v4.2.1). Only X-linked genes with at least 10 SNP-overlapping reads across all samples were considered for allelic ratio calculations. The allelic ratio was defined as Xi/(Xi + Xa), where Xi and Xa represent the inactive and active X chromosome alleles, respectively. Gene categories based on initial expression levels and promoter chromatin state were obtained from Nesterova et al^27^, and gene silencing kinetics data were sourced from Bowness et al^46^.

The SMCHD1 dependency of X-linked genes was previously defined^78^. In this study, we defined the SETDB1 dependency of X-linked genes by comparing allelic ratios of X-linked genes in NPCs (NPC differentiation day 28) between control and SETDB1 acute depletion conditions. Escapee are excluded from this classification. Specifically, genes are classified as SETDB1-independent if allelic ratios in both control and dTAG13-treated conditions are below 0.03. Genes are classified as SETDB1-dependent if the allelic ratio in dTAG13-treated conditions exceeded that in control by more than 0.05. Genes not meeting either criterion are classified as SETDB1 partially-dependent. For chromosomal regions covered in all three clones (A12A10, P2F7, and G6D2), these classification criteria were applied to all clones. For regions informative only in A12A10 and P2F7, the criteria were applied to these two clones. SMCHD1 dependency and SETDB1 dependency classifications were then cross-compared, with results shown in Supplementary Fig. 1d.

### Differential gene expression analysis of 4sU-seq and ChrRNA-seq

In order to call differential gene expression between control and degron from 4sU-seq and ChrRNA-seq samples, raw FASTQ files containing paired-end reads were first aligned to a custom rRNA reference using Bowtie2 (v2.3.5.1)^68^ to remove rRNA contaminants. Reads mapping to rRNA were discarded, and the remaining unmapped read pairs were subsequently aligned to the mouse genome (mm10) using STAR (v2.7.9a)^76^. STAR was run with the following parameters: “--twopassMode Basic --outSAMstrandField intronMotif --outFilterMismatchNoverReadLmax 0.06 --outFilterMultimapNmax 100 --alignEndsType EndToEnd –winAnchorMultimapNmax 100”. Alignments were sorted, and strand split using SAMtools (v1.16)^69^. Data were normalised to 10 million mapped reads. For data visualisation, BEDtools (v2.30)^71^ was used to generate bigWig coverage tracks, which were then visualised using IGV. Metagene profiles and heatmaps were created using deepTools (v3.5.2)^72^, supplemented with custom Python scripts. Differential expression analysis of genes and transposable elements was performed using TEtranscripts (v2.2.1)^79^, applying an adjusted p-value threshold of 0.05 to define significance.

### Statistics and reproducibility

No statistical methods were used to predetermine sample sizes. The number of X-linked genes included in each analysis was determined by selecting genes with at least 10 allelic reads across the compared samples. All experiments, including western blotting, imaging and sequencing, were performed using at least two biological replicates, with the exception of tests using a reciprocal cell line or an alternative antibody shown in Supplementary Fig. 4, which were performed as a single replicate. The statistical analysis was conducted with R (v 4.2.3). *p*-values were calculated using a two-sided paired Student’s *t* test for all the X-linked gene silencing comparisons. The threshold for statistical significance was *p* < 0.05 using TEtranscripts software. The number of measurements and independent experiments, and the statistical test used for each analysis performed, were described in the corresponding figure legends. Imaging data was plotted with Igor Pro 9 (WaveMetrics) and either depicted as box plots or violin plots and an unpaired single tailed students *T* test was used for statistical analysis between individual datasets of two biological replicates to analyse significant differences within the populations on a single-cell level.

### Reporting summary

Further information on research design is available in the Nature Portfolio Reporting Summary linked to this article.

## Supplementary information

Supplementary Information

Description of Additional Supplementary Files

Supplementary Data 1

Reporting Summary

Transparent Peer Review file

## Source data

Source data

## Data Availability

Chromatin-RNA-seq (GSE310108 [https://www.ncbi.nlm.nih.gov/geo/query/acc.cgi?acc=GSE310108]), 4sU-seq (GSE309421 [https://www.ncbi.nlm.nih.gov/geo/query/acc.cgi?acc=GSE309421]) and native (GSE312320 [https://www.ncbi.nlm.nih.gov/geo/query/acc.cgi?acc=GSE312320]) and crosslinked (GSE309423 [https://www.ncbi.nlm.nih.gov/geo/query/acc.cgi?acc=GSE309423]) ChIP-seq datasets generated in this study are available from NCBI Gene Expression Omnibus (GEO) under SuperSeries GSE309424. Source data are provided in this paper.

## Code availability

This paper does not report any original code.
