# Supplementary material for: SETDB1 and HUSH modulate Xist RNA levels during establishment of X chromosome inactivation
Source: Nat Commun. 2026 Apr 9;17:5029. doi: 10.1038/s41467-026-71569-8 (PMC13243529; doi:10.1038/s41467-026-71569-8)
Supplement: Supplementary file 1 — Supplementary Information [file 41467_2026_71569_MOESM1_ESM.pdf]

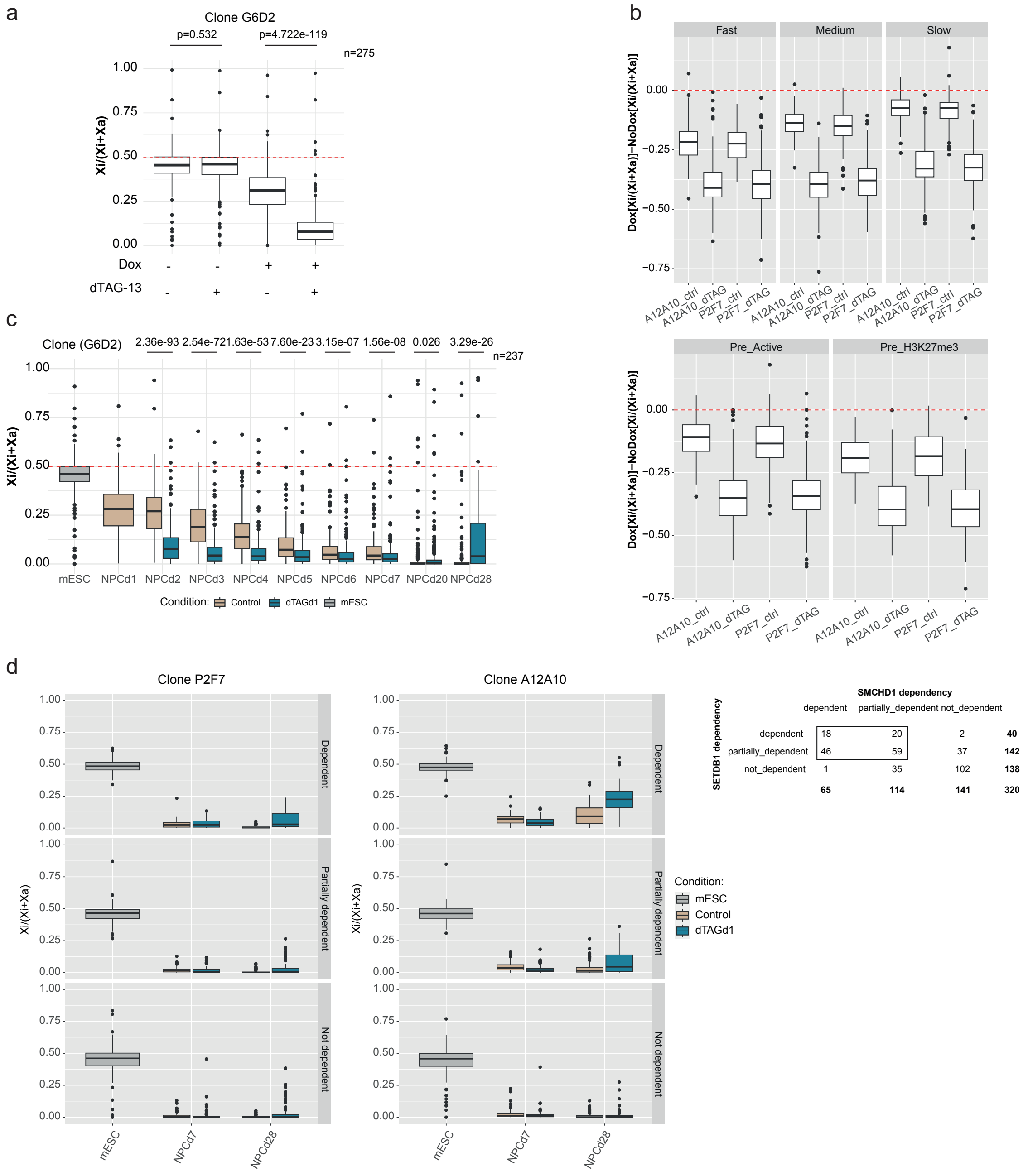

Supplementary Fig. 1: Different gene groups are affected by SETDB1 depletion in initiation and maintenance of X chromosome silencing.

(a) Boxplot showing the allelic ratio (ranging from 0 to 1) of X-linked genes (n=275) from ChrRNA-seq analysis of additional independent clone with FKBP12^{F36V}-tagged SETDB1 (G6D2) using mESCs in different conditions indicated at the bottom. G6D2 clone has a truncated X chromosome which has retained only the 0-138M region of the Xi. The red dashed line indicates allelic ratio of 0.5. P values were calculated using a two-sided paired t-test. In boxplots, centre lines indicate the median, box limits indicate the first and third quartiles and

whiskers indicate 1.5× the interquartile range (IQR). Source data are provided as a Source Data file.

(b) Boxplot showing the allelic ratio difference between samples expressing Xist (+dox and +dTAG+dox) and matched controls (mESC and +dTAG, respectively) for genes grouped according to their silencing kinetics (top) and their initial promoter chromatin state (bottom) as previously defined in wild-type cells. The red dashed line indicates no difference in allelic ratio. In boxplots, centre lines indicate the median, box limits indicate the first and third quartiles and whiskers indicate 1.5× the interquartile range (IQR). Source data are provided as a Source Data file.

(c) Boxplot showing the allelic ratio (ranging from 0 to 1) of X-linked genes (n=237) from ChrRNA-seq analysis of an additional independent clone with FKBP12^{F36V}-tagged SETDB1 (G6D2) upon differentiation of mESCs into NPCs. Boxplots represent SETDB1 depleted cells (blue) and matched wild-type controls (beige). The red dashed line indicates allelic ratio of 0.5. P values were calculated using a two-sided paired t-test. In boxplots, centre lines indicate the median, box limits indicate the first and third quartiles and whiskers indicate 1.5× the interquartile range (IQR). Source data are provided as a Source Data file.

(d) Left: Boxplots showing allelic ratio (ranging from 0 to 1) of X-linked genes categorized based on their SMCHD1 dependency for silencing as previously defined (see Methods). Right: Table showing the number of overlapping genes in each SMCHD1 dependency group (previously defined) and SETDB1 dependency group (see Methods). The rectangle highlights the overlap between SMCHD1 and SETDB1 dependent and partially dependent groups. In boxplots, centre lines indicate the median, box limits indicate the first and third quartiles and whiskers indicate 1.5× the interquartile range (IQR). Source data are provided as a Source Data file.

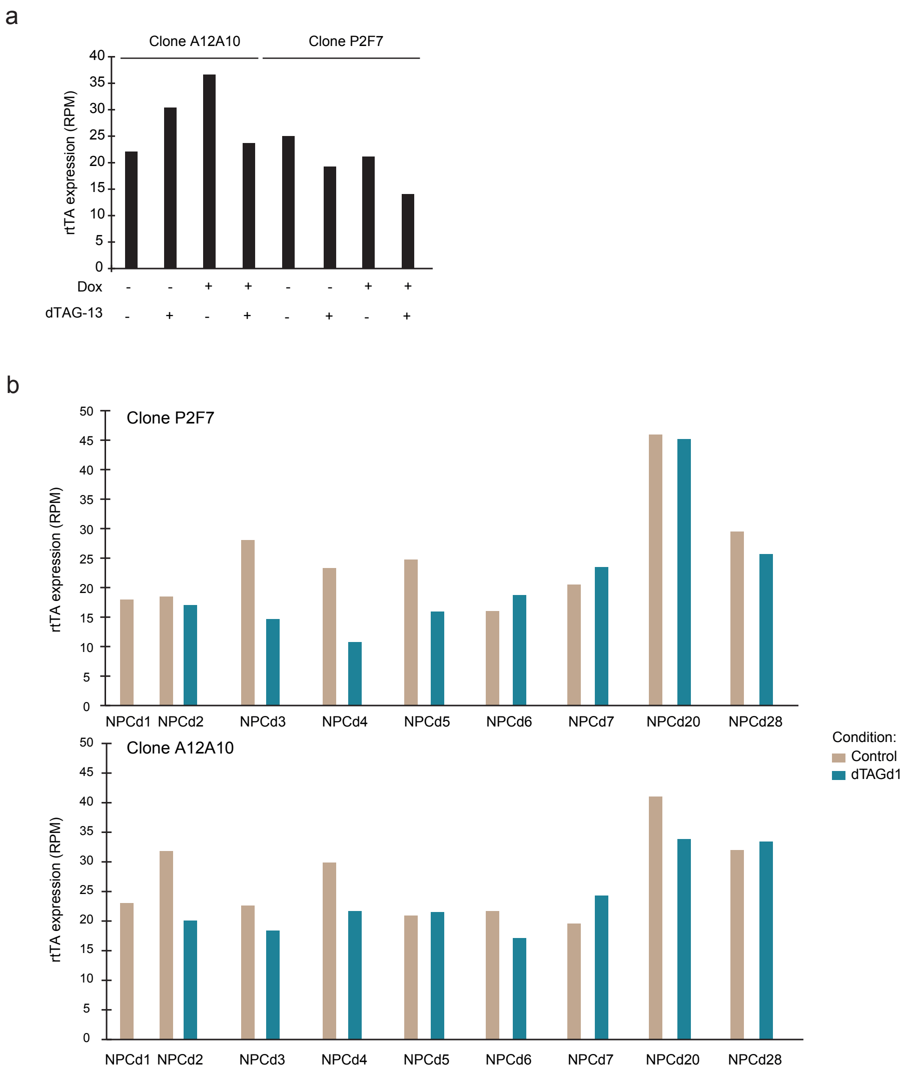

Supplementary Fig. 2: Expression levels of rtTA transgene in SETDB1 depletion experiments in mESC and NPC differentiation

(a) Bar plot showing rtTA transgene RNA levels (RPM, reads per million mapped reads) from ChrRNA-seq analysis of FKBP12^{F36V}-tagged SETDB1 mESCs (clones A12A10 and P2F7) in different conditions as indicated at the bottom, matching data in Fig. 1b and 2a. Source data are provided as a Source Data file.

(b) Bar plot showing rtTA transgene RNA levels (RPM, reads per million mapped reads) from ChrRNA-seq analysis of FKBP12^{F36V}-tagged SETDB1 mESC differentiated into NPC (clones A12A10 and P2F7) in different conditions as indicated at the bottom, matching data in Fig. 1c and 2b. Source data are provided as a Source Data file.

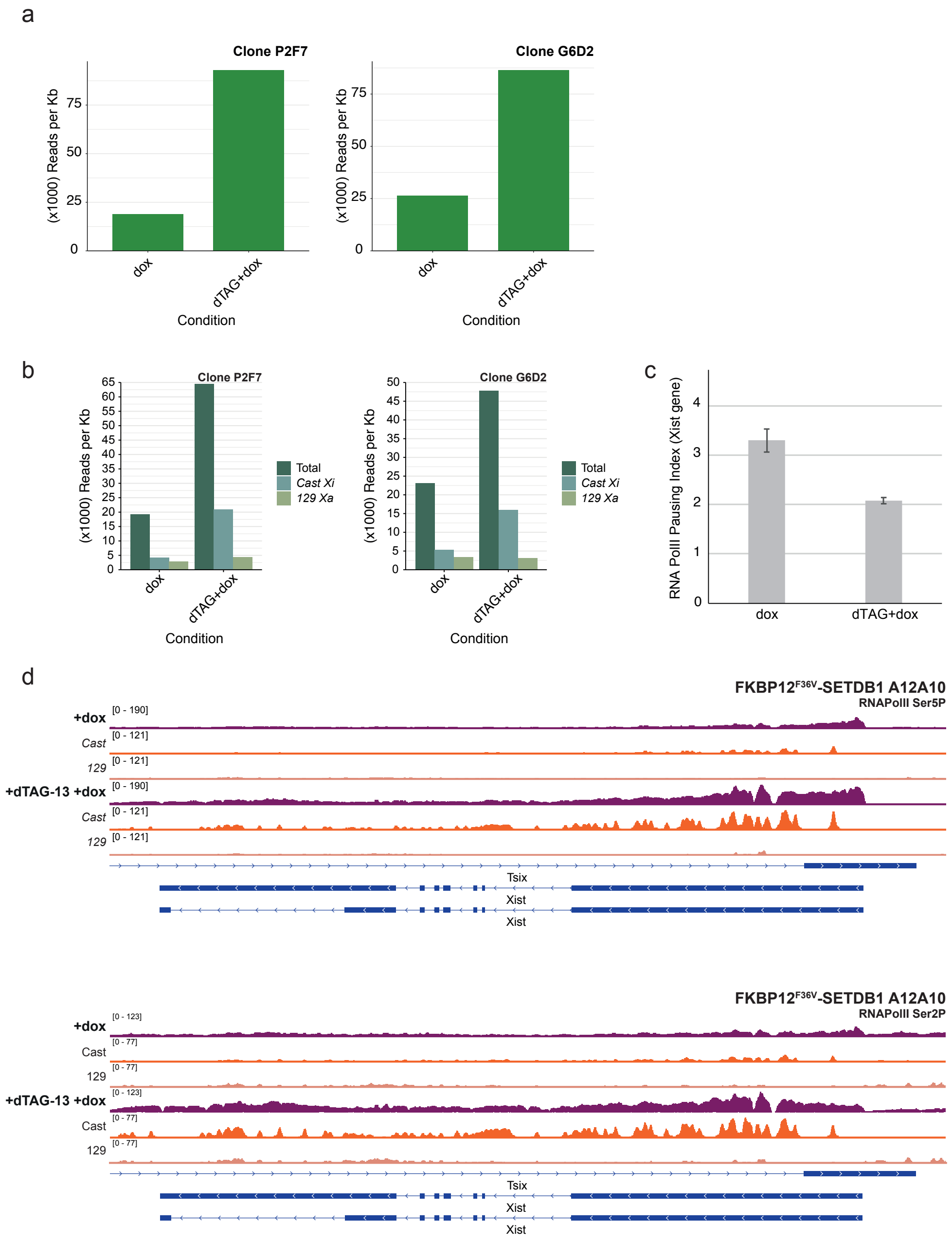

Supplementary Fig. 3: Effects of SETDB1 depletion on RNA Polymerase II engagement at the Xist locus.

(a) Bar plots showing quantification of the number of Xist reads from 4sU-seq in control (+dox) and SETDB1 depleted (+dTAG-13+dox) conditions in two independent FKBP12^{F36V}-tagged SETDB1 mESC clones (P2F7 and G6D2) as shown in Fig. 3b. Source data are provided as a Source Data file.

(b) Quantification of the number of reads from RNAPII cChIP-seq over the Xist locus in control (+dox) and SETDB1 depleted (+dTAG-13+dox) conditions in two independent FKBP12^{F36V}-tagged SETDB1 mESC clones (P2F7 and G6D2) as shown in Fig. 3e. Quantification of reads assigned to Cast and 129 alleles using SNPsplit are also shown. Source data are provided as a Source Data file.

(c) Bar plot showing the average RNA Polymerase II promoter pausing index calculated for the Xist gene in Xist expressing control (+dox) and upon SETDB1 depletion (dTAG+dox). Promoter region is considered from -50bp upstream to 300bp downstream of TSS. Error bars show standard deviation. Source data are provided as a Source Data file.

(d) Representative tracks showing RNAPII Serine 5 Phosphorylation (top) and RNAPII Serine 2 Phosphorylation (bottom) cChIP-seq reads over the Xist locus in Control (+dox) and SETDB1 depleted (+dTAG-13+dox) conditions in FKBP12^{F36V}-tagged SETDB1 mESCs (clone A12A10). Orange tracks represent reads assigned to Cast and 129 alleles using SNPsplit. Cast corresponds to the Xi in this cell line.

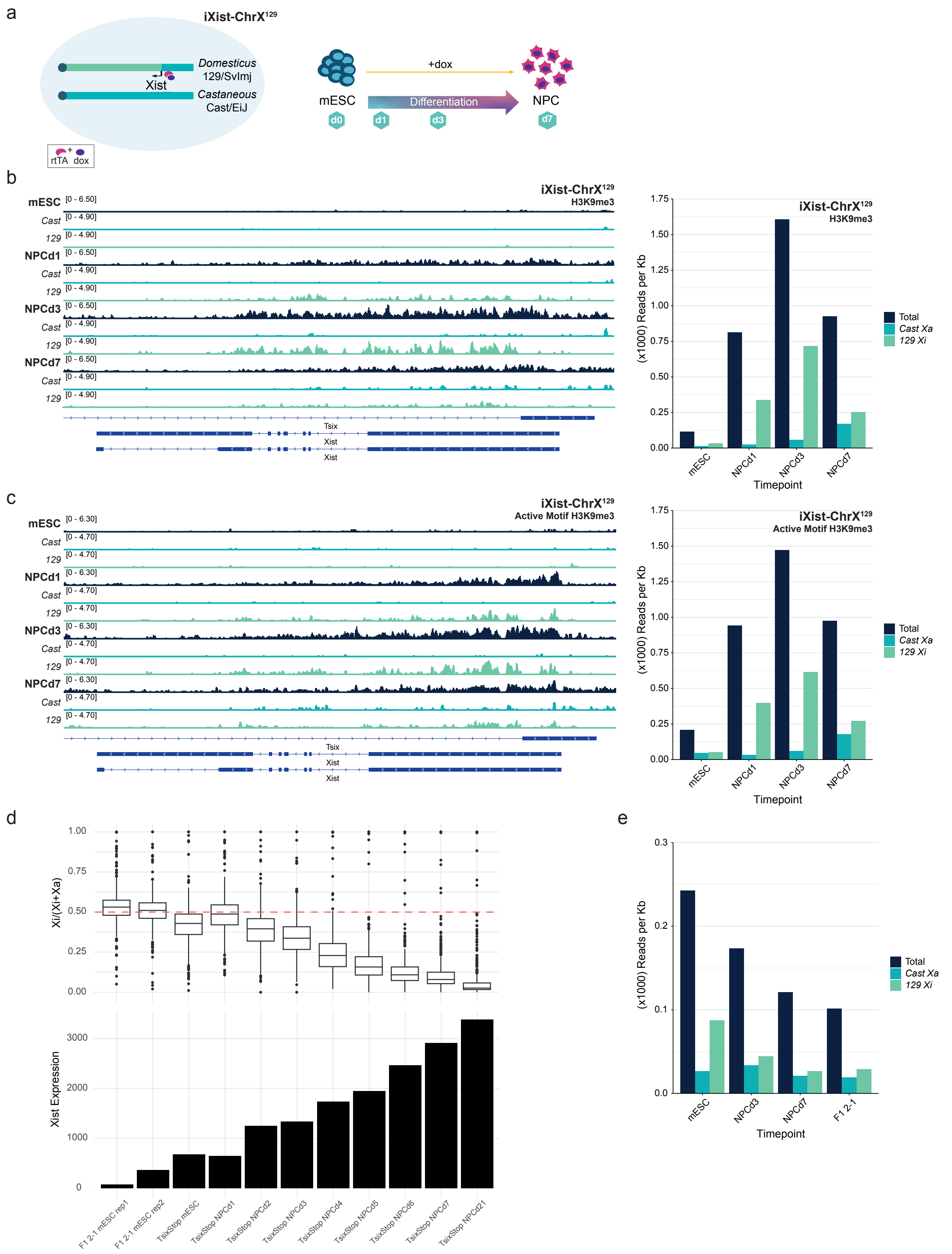

Supplementary Fig. 4: H3K9me3 accumulation on the transcribed Xist allele in iXist-chrX^{129} mESCs during NPC differentiation.

(a) Schematic illustrating experimental setup for Cast x 129 F1 mouse ES cell model, iXist-chrX^{129}, in which the 129 X chromosome has an inducible Xist allele.

(b) Representative snapshot showing H3K9me3 cChIP-seq reads over the Xist locus in NPC differentiation of iXist-chrX^{129} mESCs. Lighter shades represent reads assigned to each Cast and 129 alleles using SNPsplit. 129 corresponds to the Xi in this cell line. Quantification of the number of reads is shown in the bar plot on the right, including the quantification of reads assigned to Cast and 129 alleles. Source data are provided as a Source Data file.

(c) Representative snapshot showing H3K9me3 cChIP-seq reads over the Xist locus in NPC differentiation of iXist-chrX^{129} mESCs performed with an alternative anti-H3K9me3 antibody (Active Motif). Lighter shades represent reads assigned to Cast and 129 alleles using SNPsplit. 129 corresponds to the Xi in this cell line. Quantification of the number of reads is shown in the bar plot on the right, including the quantification of reads assigned to Cast and 129 alleles. Source data are provided as a Source Data file.

(d) Top: Boxplot showing the allelic ratio (ranging from 0 to 1) of X-linked genes from ChrRNA-seq analysis of F1 2-1 and TsixStop mESCs and differentiation of TsixStop line into NPCs. The red dashed line indicates allelic ratio of 0.5. Bottom: Bar plot showing Xist RNA levels (RPM, reads per million mapped reads) from ChrRNA-seq analysis of F1 2-1 and TsixStop mESCs and differentiation of TsixStop mESCs into NPCs. Xist is expressed from its native promoter in these cell lines. In boxplots, centre lines indicate the median, box limits indicate the first and third quartiles and whiskers indicate 1.5× the interquartile range (IQR). Source data are provided as a Source Data file.

(e) Bar plot showing quantification of an independent cChIP-seq replicate showing the number of H3K9me3 cChIP-seq reads over the Xist locus in F1 2-1 mESCs as well as TsixStop mESCs and NPCs, including the quantification of reads assigned to each allele (replicate of the experiment in Fig. 4c). Source data are provided as a Source Data file.

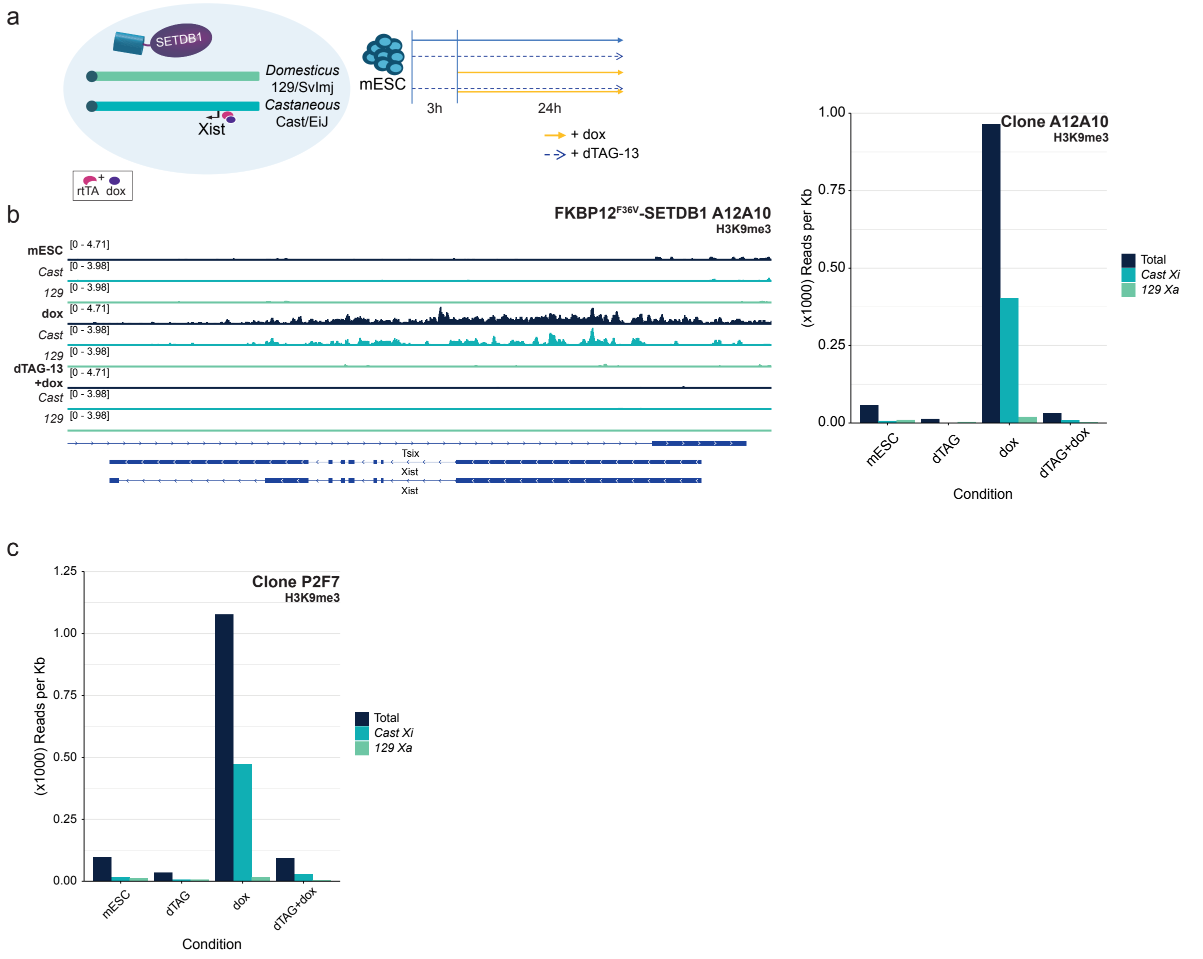

Supplementary Fig. 5: SETDB1-dependent H3K9me3 deposition on the transcribed Xist allele in undifferentiated mESCs.

(a) Schematic illustrating the experimental setup for SETDB1 depletion prior to Xist expression in mESCs.

(b) Representative snapshot showing H3K9me3 cChIP-seq reads over the Xist locus in a FKBP12^{F36V}-tagged SETDB1 mESC line (clone A12A10) without Xist expression (mESC), upon Xist induction (+dox) and upon Xist induction in the absence of SETDB1 (+dTAG+dox). Lighter shades represent reads assigned to Cast and 129 alleles using SNPsplit. Cast corresponds to Xi in this cell line. Quantification of the number of reads is shown in the bar plot on the right, including the quantification of reads assigned to Cast and 129 alleles. Source data are provided as a Source Data file.

(c) Bar plot showing the quantification of the number of reads for H3K9me3 cChIP-seq in an independent FKBP12^{F36V}-tagged SETDB1 mESC line (clone P2F7), including the quantification of reads assigned to Cast and 129 alleles as in Supplementary Fig. 5b. Source data are provided as a Source Data file.

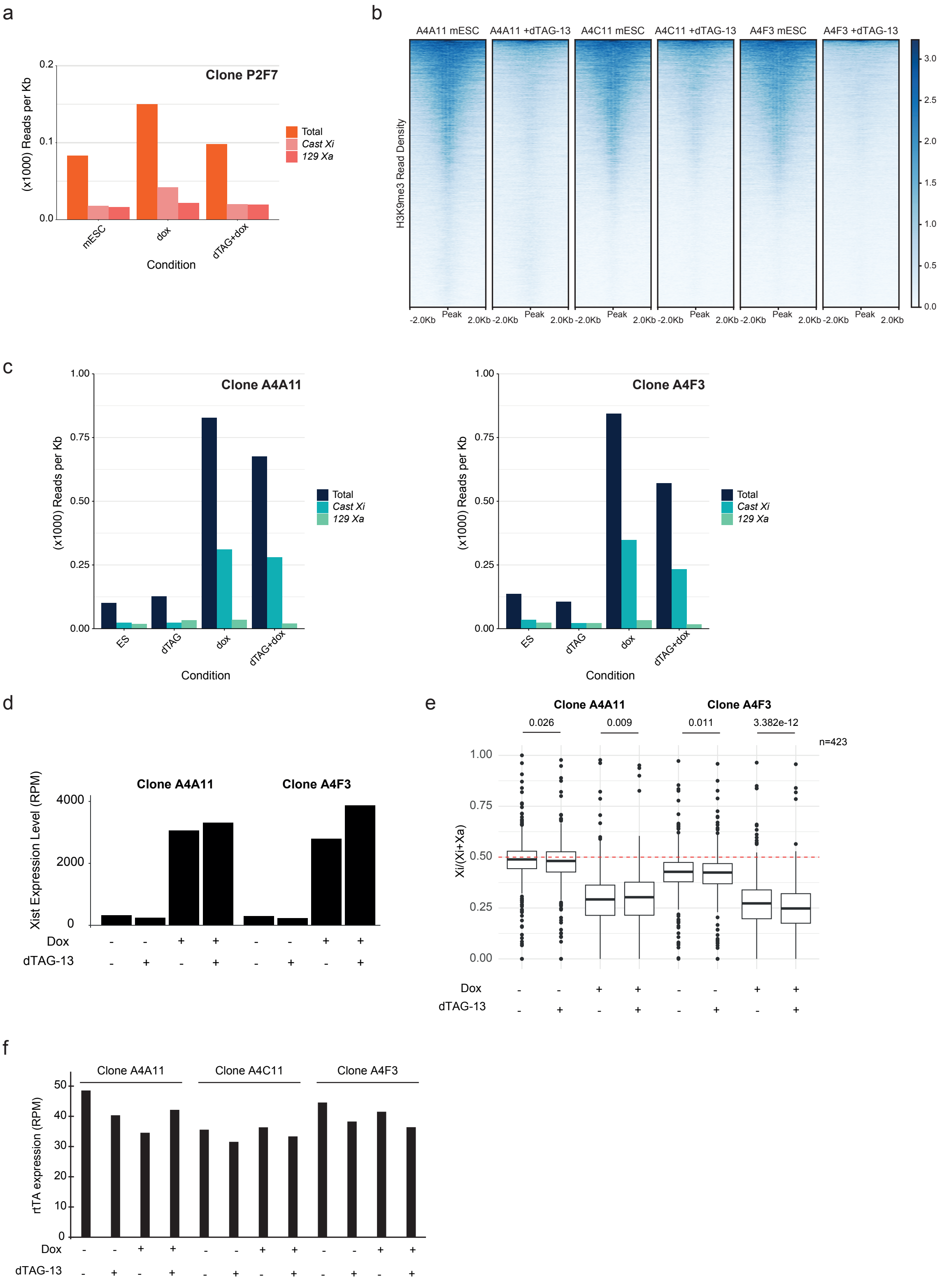

Supplementary Fig. 6: SETDB1-dependent accumulation of KAP1 on the transcribed Xist allele

(a) Boxplot showing quantification of the number of KAP1 ChIP-seq reads over the Xist locus in mESC, Xist expression control (+dox) and SETDB1 depleted (+dTAG-13+dox)

conditions in an independent FKBP12^{F36V}-tagged SETDB1 mESC line (clone P2F7), including the quantification of reads assigned to Cast and 129 alleles as in Fig. 6a. Source data are provided as a Source Data file.

(b) Heatmap showing changes in H3K9me3 read density across unique KAP1 peaks following KAP1 depletion in FKBP12^{F36V}-tagged KAP1 mESC lines. Source data are provided as a Source Data file.

(c) Bar plots showing quantification of the number of H3K9me3 cChIP-seq reads over the Xist locus in mESC, Xist expression control (+dox) and KAP1 depleted (+dTAG-13+dox) conditions in two independent clones of FKBP12^{F36V}-tagged KAP1 mESC lines (clones A4A11 and A4F3), including the quantification of reads assigned to Cast and 129 alleles as in Fig. 6c. Source data are provided as a Source Data file.

(d) Bar plot showing Xist RNA levels (RPM, reads per million mapped reads) from ChrRNA-seq analysis of two additional independent FKBP12^{F36V}-tagged KAP1 mESC lines (clones A4A11 and A4F3) in different conditions as indicated at the bottom, as in Fig. 6d. Source data are provided as a Source Data file.

(e) Boxplot showing the allelic ratio (ranging from 0 to 1) of X-linked genes (n=423) from ChrRNA-seq analysis of another two independent FKBP12^{F36V}-tagged KAP1 mESC lines (clone A4A11 and A4F3) in different conditions indicated at the bottom, as in Fig. 6e. The red dashed line indicates allelic ratio of 0.5. P values were calculated using a two-sided paired t-test. In boxplots, centre lines indicate the median, box limits indicate the first and third quartiles and whiskers indicate 1.5× the interquartile range (IQR). Source data are provided as a Source Data file.

(f) Bar plot showing rtTA transgene RNA levels (RPM, reads per million mapped reads) from ChrRNA-seq analysis of FKBP12^{F36V}-tagged KAP1 mESC (clones A4A11, A4C11 and A4F3) in different conditions as indicated at the bottom, matching data in Fig. 6d-e and Supplementary Fig 6d-e. Source data are provided as a Source Data file.

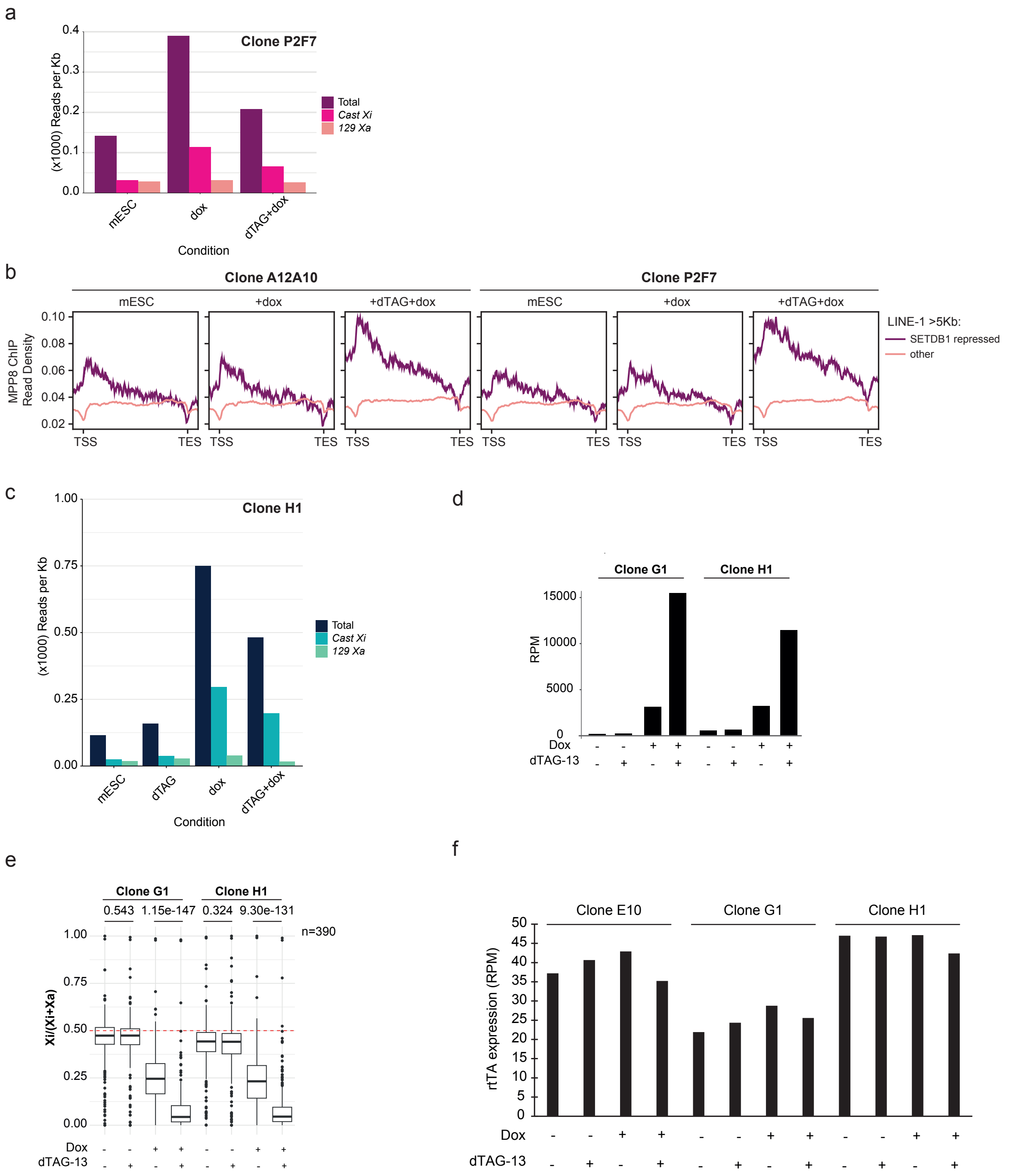

Supplementary Fig. 7: The HUSH complex plays a role in SETDB1-mediated H3K9me3 on the transcribed Xist allele.

(a) Bar plot showing quantification of MPP8 ChIP-seq reads over the Xist locus in mESC, Xist expression control (+dox) and SETDB1 depleted (+dTAG-13+dox) conditions in an additional independent FKBP12^{F36V}-tagged SETDB1 mESC (clone P2F7), including quantification of reads assigned to Cast and 129 alleles, as in Fig. 7a. Source data are provided as a Source Data file.

(b) Metaplot of MPP8 ChIP-seq signal over full-length (> 5Kb) LINE1 elements upregulated in SETDB1 depleted cells (purple line; n=198) and remaining LINE1 elements (pink line; n=12307) in FKBP12^{F36V}-tagged SETDB1 mESC (clones A12A10 and P2F7) in different conditions as indicated at the top.

(c) Bar plot showing quantification of H3K9me3 cChIP-seq reads over the Xist locus in mESC, Xist expression control (+dox) and MPP8 depleted (+dTAG-13+dox) conditions in an additional independent FKBP12^{F36V}-tagged MPP8 mESC line (clone H1), including the quantification of reads assigned to Cast and 129 alleles, as in Fig. 7c. Source data are provided as a Source Data file.

(d) Bar plot showing Xist RNA levels (RPM, reads per million mapped reads) from ChrRNA-seq analysis of two additional independent FKBP12^{F36V}-tagged MPP8 mESC lines (clones G1 and H1) in different conditions as indicated at the bottom, as in Fig. 7d. Source data are provided as a Source Data file.

(e) Boxplot showing the allelic ratio (ranging from 0 to 1) of X-linked genes from ChrRNA-seq analysis of another two independent FKBP12^{F36V}-tagged MPP8 mESC lines (clone G1 and H1) in different conditions indicated at the bottom, as in Fig. 7e. The red dashed line indicates allelic ratio of 0.5. P values were calculated using a two-sided paired t-test. In boxplots, centre lines indicate the median, box limits indicate the first and third quartiles and whiskers indicate 1.5× the interquartile range (IQR). Source data are provided as a Source Data file.

(f) Bar plot showing rtTA transgene RNA levels (RPM, reads per million mapped reads) from ChrRNA-seq analysis of FKBP12^{F36V}-tagged MPP8 mESC (clones E10, G1 and H1) in different conditions as indicated at the bottom, matching data in Fig. 7d-e and Supplementary Fig 7d-e. Source data are provided as a Source Data file.

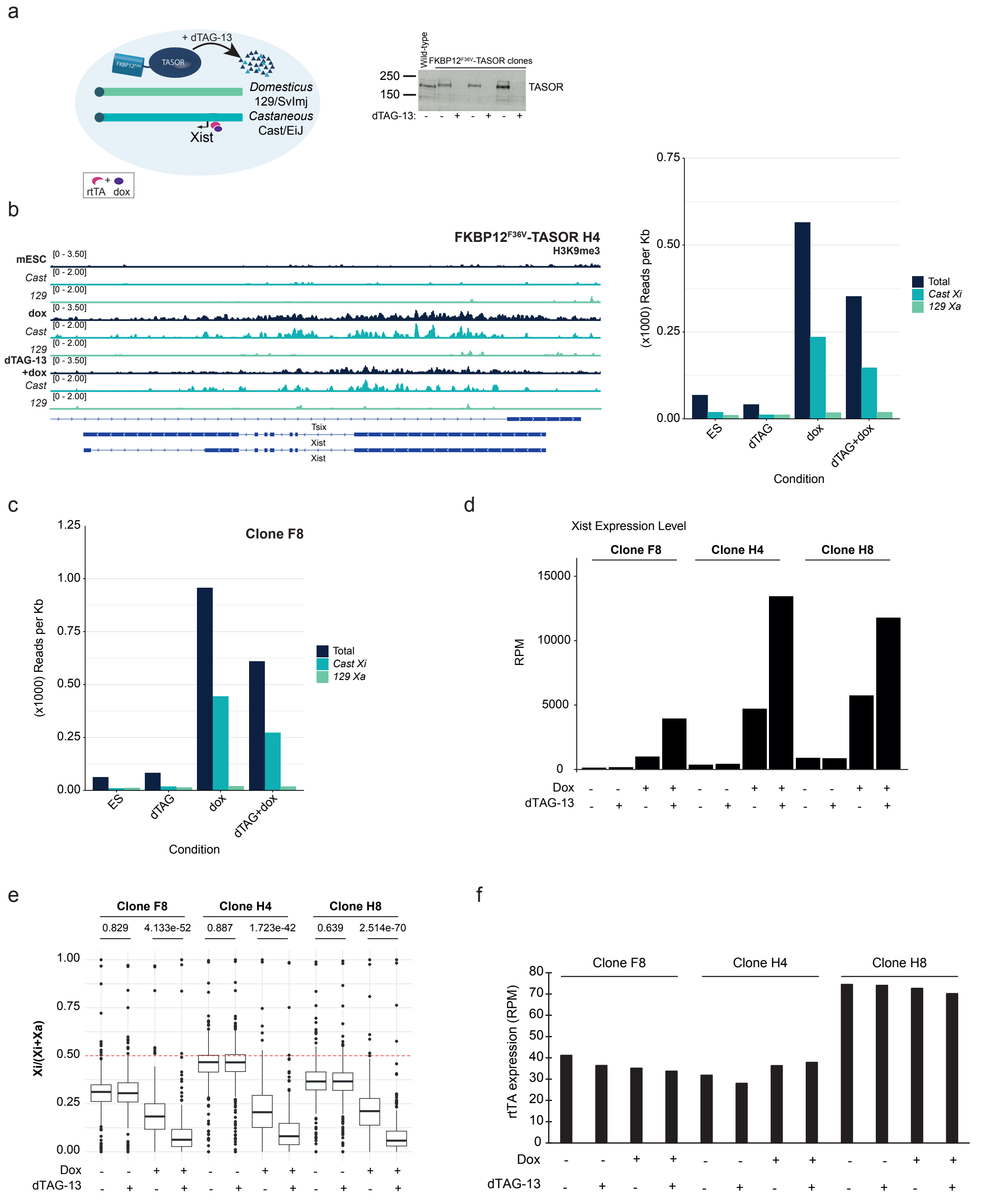

Supplementary Fig. 8: Depletion of TASOR confirming role for the HUSH complex in SETDB1-mediated H3K9me3 on the transcribed Xist allele

(a) Schematic illustrating Cast x 129 F1 mESC model engineered to allow depletion of endogenous TASOR using dTAG-13. Western blot shows protein depletion after 2h of dTAG-13 treatment. Source data are provided as a Source Data file.

(b) Representative snapshot showing H3K9me3 cChIP-seq reads over the Xist locus in mESC, Xist expression control (+dox) and TASOR depleted (+dTAG-13+dox) conditions in FKBP12^{F36V}-tagged TASOR mESCs (clone H4). Lighter shades represent reads assigned to each Cast and 129 alleles) using SNPsplit. Cast corresponds to the Xi in this cell line. Quantification of the number of reads is shown in the bar plot on the right, including the quantification of reads assigned to Cast and 129 alleles. Source data are provided as a Source Data file.

(c) Bar plot showing the quantification of H3K9me3 cChIP-seq reads over the Xist locus in mESC, Xist expression control (+dox) and TASOR depleted (+dTAG-13+dox) conditions in additional independent FKBP12^{F36V}-tagged TASOR mESC line (clone F8), including the quantification of reads assigned to Cast and 129 alleles, as in Supplementary Fig. 7b. Source data are provided as a Source Data file.

(d) Bar plot showing Xist RNA levels (RPM, reads per million mapped reads) from ChrRNA-seq analysis of three independent FKBP12^{F36V}-tagged TASOR mESC lines (clones F8, H4 and H8) in different conditions as indicated at the bottom. Source data are provided as a Source Data file.

(e) Boxplot showing the allelic ratio (ranging from 0 to 1) of X-linked genes from ChrRNA-seq analysis of three independent FKBP12^{F36V}-tagged TASOR mESC lines (clones F8, H4 and H8) in different conditions indicated at the bottom. The red dashed line indicates allelic ratio of 0.5. P values were calculated using a two-sided paired t-test. In boxplots, centre lines indicate the median, box limits indicate the first and third quartiles and whiskers indicate 1.5× the interquartile range (IQR). Source data are provided as a Source Data file.

(f) Bar plot showing rtTA transgene RNA levels (RPM, reads per million mapped reads) from ChrRNA-seq analysis of FKBP12^{F36V}-tagged TASOR mESC (clones F8, H4 and H8) in different conditions as indicated at the bottom, matching data in Supplementary Fig 8d-e. Source data are provided as a Source Data file.
