# Supplementary material for: SETDB1 and HUSH modulate Xist RNA levels during establishment of X chromosome inactivation
Source: Nat Commun. 2026 Apr 9;17:5029. doi: 10.1038/s41467-026-71569-8 (PMC13243529; doi:10.1038/s41467-026-71569-8)
Supplement: Supplementary file 2 — Description of Additional Supplementary Files [file 41467_2026_71569_MOESM2_ESM.pdf]

Description of Additional Supplementary Files

Supplementary Data 1: listing differentially expressed genes (DEGs) between Control_vs_dTAG and Dox_vs_dTAGDox from ChrRNA-seq experiments and Dox vs dTAGDoxU20min from 4sU-seq experiment. Statistical tests were performed using the default method implemented in the TEtranscripts program, and p-values were adjusted for multiple testing.
