# Supplementary material for: SETDB1 and HUSH modulate Xist RNA levels during establishment of X chromosome inactivation
Source: Nat Commun. 2026 Apr 9;17:5029. doi: 10.1038/s41467-026-71569-8 (PMC13243529; doi:10.1038/s41467-026-71569-8)
Supplement: Supplementary file 4 — Reporting Summary [file 41467_2026_71569_MOESM4_ESM.pdf]

nature portfolio

Corresponding author(s): Neil Brockdorff

Last updated by author(s): Oct 15, 2025

Reporting Summary

Nature Portfolio wishes to improve the reproducibility of the work that we publish. This form provides structure for consistency and transparency in reporting. For further information on Nature Portfolio policies, see our Editorial Policies and the Editorial Policy Checklist.

Statistics

For all statistical analyses, confirm that the following items are present in the figure legend, table legend, main text, or Methods section.

n/a	Confirmed	
☐	☒	The exact sample size (*n*) for each experimental group/condition, given as a discrete number and unit of measurement
☐	☒	A statement on whether measurements were taken from distinct samples or whether the same sample was measured repeatedly
☐	☒	The statistical test(s) used AND whether they are one- or two-sided<br>*Only common tests should be described solely by name; describe more complex techniques in the Methods section.*
☒	☐	A description of all covariates tested
☒	☐	A description of any assumptions or corrections, such as tests of normality and adjustment for multiple comparisons
☐	☒	A full description of the statistical parameters including central tendency (e.g. means) or other basic estimates (e.g. regression coefficient) AND variation (e.g. standard deviation) or associated estimates of uncertainty (e.g. confidence intervals)
☐	☒	For null hypothesis testing, the test statistic (e.g. *F*, *t*, *r*) with confidence intervals, effect sizes, degrees of freedom and *P* value noted<br>*Give P values as exact values whenever suitable.*
☒	☐	For Bayesian analysis, information on the choice of priors and Markov chain Monte Carlo settings
☒	☐	For hierarchical and complex designs, identification of the appropriate level for tests and full reporting of outcomes
☒	☐	Estimates of effect sizes (e.g. Cohen's *d*, Pearson's *r*), indicating how they were calculated

Our web collection on statistics for biologists contains articles on many of the points above.

Software and code

Policy information about availability of computer code

Data collection	Sequencing data for ChrRNA-seq, ChIP-Seq and 4sU-seq were generated using the Illumina NextSeq 500 platform. RNA-FISH images were acquired with the VisiTech iSIM super-resolution array scanning module built around an Olympus IX83 inverted microscope. Western blot images were developed onto Amersham Hyperfilm ECL (GE Healthcare) using a Konica SRX-101A Medical Film Processor.
Data analysis	Bowtie2 (2.3.5 & 2.4.5), SAMtools (1.16.1), STAR (v2.5.2b & 2.7.9a), IGV (2.17.1), Subread (1.5.2), Picard tools (2.25.0), deeptools (3.5.5), bedtools (v2.27.1), TEtranscripts (v2.2.1), R (4.1.0 & 4.2.1), and tidyverse (2.0.0).

For manuscripts utilizing custom algorithms or software that are central to the research but not yet described in published literature, software must be made available to editors and reviewers. We strongly encourage code deposition in a community repository (e.g. GitHub). See the Nature Portfolio guidelines for submitting code & software for further information.

Data

Policy information about availability of data

All manuscripts must include a data availability statement. This statement should provide the following information, where applicable:

- Accession codes, unique identifiers, or web links for publicly available datasets
- A description of any restrictions on data availability
- For clinical datasets or third party data, please ensure that the statement adheres to our policy

Chromatin-RNA-seq, 4sU-seq and native and crosslinked ChIP-seq datasets are available from NCBI Gene Expression Omnibus (GEO) SuperSeries GSE309424.
The mouse genome (mm10) sequence and gene annotation were downloaded from UCSC genome browser (https://hgdownload.soe.ucsc.edu/downloads.html).

The whole genome collections of SNP and short indel variants for mouse strains 129S1 and Cast/EiJ (mpg.v5) was downloaded from mouse genome project (https://www.sanger.ac.uk/data/mouse-genomes-project/). Gene categories including initial X-linked gene expression level were taken from GSE119602. Gene silencing kinetics data were taken from GSE185843. Promoter chromatin landscape of mm10 genome were retrieved from (https://github.com/guifengwei/ChromHMM_mESC_mm10).
Uncropped western blots and numerical source data are available in source data. Previously published sequencing dataset and imaging dataset used in this study have been specified in the manuscript.

Research involving human participants, their data, or biological material

Policy information about studies with human participants or human data. See also policy information about sex, gender (identity/presentation), and sexual orientation and race, ethnicity and racism.

Reporting on sex and gender	NA
Reporting on race, ethnicity, or other socially relevant groupings	NA
Population characteristics	NA
Recruitment	NA
Ethics oversight	NA

Note that full information on the approval of the study protocol must also be provided in the manuscript.

Field-specific reporting

Please select the one below that is the best fit for your research. If you are not sure, read the appropriate sections before making your selection.

☒ Life sciences ☐ Behavioural & social sciences ☐ Ecological, evolutionary & environmental sciences

For a reference copy of the document with all sections, see nature.com/documents/nr-reporting-summary-flat.pdf

Life sciences study design

All studies must disclose on these points even when the disclosure is negative.

Sample size	No statistical methods were used to predetermine sample size for sequencing analysis.<br>For RNA-seq, ChIP-seq and 4sU-seq either multiple independent clones or 2-3 independent repeats for a single clone were chosen, according to common practice in the field. This design ensures the results are reproducible.
Data exclusions	We confirm that no data were excluded from the analyses.
Replication	The number of biological replicates are indicated in the text, figure legend, or method section.
Randomization	For tissue culture–based experiments, all wells within each biological replicate were derived from the same batch of cells and randomly assigned to each experimental condition.
Blinding	Investigators were not blinded during experiments or outcome assessments. Blinding was not required, as all data acquisition and analyses were performed using automated software and algorithms without subjective scoring.

Reporting for specific materials, systems and methods

We require information from authors about some types of materials, experimental systems and methods used in many studies. Here, indicate whether each material, system or method listed is relevant to your study. If you are not sure if a list item applies to your research, read the appropriate section before selecting a response.

Materials & experimental systems

n/a	Involved in the study	
☐	☒	Antibodies
☐	☒	Eukaryotic cell lines
☒	☐	Palaeontology and archaeology
☒	☐	Animals and other organisms
☒	☐	Clinical data
☒	☐	Dual use research of concern
☒	☐	Plants

Methods

n/a	Involved in the study	
☐	☒	ChIP-seq
☒	☐	Flow cytometry
☒	☐	MRI-based neuroimaging

Antibodies

Antibodies used	Rabbit polyclonal anti-H3K9me3 (Abcam, Cat # ab8898; RRID:AB_306848); Mouse monoclonal anti-H3K9me3 (Active Motif, Cat #61013; RRID:AB_2687870); Rabbit polyclonal anti-SETDB1 (Proteintech, Cat# 11231-1-AP; RRID:AB_2186069); Rabbit polyclonal anti-MPP8 (Proteintech, Cat# 16796-1-AP, RRID:AB_2266644); Rabbit polyclonal anti-KAP1 (Abcam, Cat# ab10484, RRID:AB_297223); Mouse monoclonal anti-KAP1 (Abcam, Cat# ab22553, RRID:AB_447151); Rabbit polyclonal anti-FAM208A (Novus Biologicals, Cat# NBP1-90673, RRID:AB_11006471); Rabbit monoclonal anti-RBP1 NTD (D8L4Y) (Cell Signaling Technology, Cat# 14958, RRID:AB_2687876); Rabbit monoclonal anti-phospho-RBP1 CTD (Ser5) (D9N5I) (Cell Signaling Technology, Cat# 13523, RRID:AB_2798246); Rabbit monoclonal anti-phospho-Rpb1 CTD (Ser2) (E1Z3G) (Cell Signaling Technology, Cat# 13499, RRID:AB_2798238); Rabbit monoclonal anti-METTL3 (Abcam, Cat # ab195352, RRID:AB_2721254); Donkey Polyclonal Anti-Rabbit IgG, Whole Ab ECL Antibody, HRP-Conjugated (Cytiva Cat# NA934, RRID:AB_772206);
Validation	Antibodies were validated by the manufacturers using knockout, knockdown, or other standard verification methods, as indicated on the respective manufacturer websites.<br>In this study, antibodies against mouse SETDB1, KAP1, MPP8, and TASOR were further validated experimentally, as the FKBPF36V insertion resulted in an upward shift in the apparent molecular weight of the tagged proteins, and/or the fusion proteins were sensitive to dTAG-13–induced degradation.<br>pAb anti-H3K9me3 (https://www.abcam.com/en-us/products/primary-antibodies/histone-h3-tri-methyl-k9-antibody-chip-grade-ab8898);<br>mAb anti-H3K9me3 (https://www.activemotif.com/catalog/details/61013/histone-h3-trimethyl-lys9-antibody-clone-mab-clone-mabi-0319);<br>pAb anti-SETDB1 (https://www.ptglab.com/products/SETDB1-Antibody-11231-1-AP.htm);<br>pAb anti-MPP8 (https://www.ptglab.com/products/MPHOSPH8-Antibody-16796-1-AP.htm);<br>pAb anti-KAP1 (https://www.abcam.com/en-us/products/primary-antibodies/kap1-antibody-ab10484);<br>mAb anti-KAP1 (https://www.abcam.com/en-us/products/primary-antibodies/kap1-antibody-20c1-ab22553);<br>pAb anti-FAM208A (https://www.novusbio.com/products/fam208a-antibody_nbp1-90673);<br>mAb anti-RBP1 NTD (D8L4Y) (https://www.cellsignal.com/products/primary-antibodies/rpb1-ntd-d8l4y-rabbit-mab/14958);<br>mAb anti-phospho-RBP1 CTD (Ser5) (D9N5I) (https://www.cellsignal.com/products/primary-antibodies/phospho-rpb1-ctd-ser5-d9n5i-rabbit-mab/13523);<br>mAb anti-phospho-Rpb1 CTD (Ser2) (E1Z3G) (https://www.cellsignal.com/products/primary-antibodies/phospho-rpb1-ctd-ser2-e1z3g-rabbit-mab/13499);<br>mAb anti-METTL3 (https://www.abcam.com/en-us/products/primary-antibodies/mettl3-antibody-epr18810-ab195352);

Eukaryotic cell lines

Policy information about cell lines and Sex and Gender in Research

Cell line source(s)	All mouse embryonic stem cell (mESC) lines used in this study were female and derived from the F1 2–1 XX mESC line (129/Sv–Cast/Ei), a gift from J. Gribnau. Using this background, we generated doxycycline-inducible endogenous Xist cell lines targeted to either the 129S allele (iXist-ChrX129) or the Cast allele (iXist-ChrXCast).<br>FKBP12F36V-tagged mESC lines were created for SETDB1, MPP8, TASOR, and KAP1 on the iXist-ChrXCast background to enable rapid protein depletion upon addition of dTAG-13 to the growth medium. The FKBP12F36V tag was inserted at the N-terminus of SETDB1, MPP8, and TASOR, and at both the N- and C-termini of KAP1 (one tag per allele).<br>The TsixStop line was also kindly provided by J. Gribnau.<br>The presence of two X chromosomes in each line was confirmed by PCR.
Authentication	All engineered cell lines were validated both at the genomic level, using PCR to confirm genetic modifications, and at the protein level, using Western blot analysis to verify expression of the intended constructs.
Mycoplasma contamination	All cell lines were routinely tested for mycoplasma contamination using the MycoAlert™ Mycoplasma Detection Kit (Lonza, LT07-318) and a Luminometer LB9509 Junior (Berthold). All cell lines used in this study tested negative for mycoplasma contamination.
Commonly misidentified lines (See ICLAC register)	No commonly misidentified lines were used in this study.

Plants

Seed stocks	Plants were not used in this study.
Novel plant genotypes	Plants were not used in this study.
Authentication	Plants were not used in this study.

ChIP-seq

Data deposition

☒ Confirm that both raw and final processed data have been deposited in a public database such as GEO.

☐ Confirm that you have deposited or provided access to graph files (e.g. BED files) for the called peaks.

Data access links<br>*May remain private before publication.*	To review GEO accession GSE309424:<br>Go to https://www.ncbi.nlm.nih.gov/geo/query/acc.cgi?acc=GSE309424<br>Enter token efqzsieobjgftkl into the box
Files in database submission	bigwig, fastq
Genome browser session<br>(e.g. UCSC)	N/A.

Methodology

Replicates	At least two biological replicate clones were analysed per experiment.
Sequencing depth	Paired-end 2x75bp;<br>The depth for the sequenced libraries ranges from 20 million to 40 million reads.
Antibodies	Rabbit polyclonal anti-H3K9me3 (Abcam, Cat # ab8898; RRID:AB_306848); Mouse monoclonal anti-H3K9me3 (Active Motif, Cat #61013; RRID:AB_2687870); Rabbit polyclonal anti-MPP8 (Proteintech, Cat# 16796-1-AP, RRID:AB_2266644); Mouse monoclonal anti-KAP1 (Abcam, Cat# ab22553, RRID:AB_447151);
Peak calling parameters	Our main analysis is focused on Xist gene only, therefore there is no peak-calling in this work. We took the Xist entire gene body for this purpose. See the Methods section for details.
Data quality	For the Xist locus analysis, the entire Xist gene body was used. In addition, we have also examined the histone modification patterns at their reported target regions to ensure that our data quality was consistent with previous published findings.
Software	Bowtie2 (2.3.5), SAMtools (1.16.1), IGV (2.17.1), Picard tools (2.25.0), deeptools (3.5.5), bedtools (v2.27.1), UCSC Tools.
