# Supplementary material for: SETDB1 and HUSH modulate Xist RNA levels during establishment of X chromosome inactivation
Source: Nat Commun. 2026 Apr 9;17:5029. doi: 10.1038/s41467-026-71569-8 (PMC13243529; doi:10.1038/s41467-026-71569-8)
Supplement: Supplementary file 5 — Transparent Peer Review file [file 41467_2026_71569_MOESM5_ESM.pdf]

nature portfolio

Peer Review File

SETDB1 and HUSH modulate Xist RNA levels during establishment of X chromosome inactivation

Corresponding Author: Professor Neil Brockdorff

This file contains all reviewer reports in order by version, followed by all author rebuttals in order by version.

Version 0:

Reviewer comments:

Reviewer #1

(Remarks to the Author)
This is an interesting and important study in which Almeida and colleagues investigate the role of the histone methyltransferase SETDB1 and the HUSH complex in regulating Xist RNA levels during the establishment and maintenance of X chromosome inactivation (XCI). Xist RNA plays a central role in initiating XCI and ensures transcriptional silencing of one of the two X chromosomes in females to achieve dosage compensation. Previous work has suggested that SETDB1 contributes to the maintenance of XCI through deposition of the repressive histone mark H3K9me3, but the mechanistic details and its potential role during XCI initiation remain unclear.

Using degron-based rapid depletion in mouse embryonic stem cells (mESCs) and engineered dox-inducible Xist expression, the authors demonstrate that SETDB1 depletion accelerates X-linked gene silencing during XCI establishment. This accelerated silencing was unexpected and correlates with a marked increase in Xist RNA levels (~ 4-5 fold), detected through chromatin-associated RNA sequencing (ChrRNA-seq) and confirmed by RNA-FISH super-resolution imaging. The spatial distribution of Xist RNA was largely confined to the X chromosome but shows some ‘spill-over’ onto neighbouring autosomes.

Further analyses show that the elevated Xist RNA levels result from increased transcription rather than RNA stabilization. Measurement of nascent RNA synthesis (4sU-seq) shows a marked increase in Xist transcription rates following SETDB1 depletion. Calibrated chromatin immunoprecipitation sequencing (cChIP-seq) for RNA polymerase II (RNAPII) shows increased occupancy and decreased promoter-proximal pausing at the Xist locus, indicating enhanced transcriptional elongation in the absence of SETDB1.

The study shows that SETDB1 mediates transcription-dependent deposition of H3K9me3 across the expressed Xist allele, and is essential for repressing Xist transcription to maintain appropriate RNA levels. This H3K9me3 deposition is also found to be dependent on the HUSH repressor complex, specifically its core subunits MPP8 and TASOR, which recruit SETDB1 in a nascent RNA-dependent manner. In contrast, they find that KAP1, a known SETDB1 adaptor is dispensable for H3K9me3 deposition and Xist regulation. Depletion of HUSH components also results in reduced H3K9me3 at the Xist locus, elevated Xist RNA levels, and accelerated X-linked gene silencing. However, HUSH-depletion does not entirely phenocopy SETDB1 depletion – their results suggest that other players may also be involved in H3K9me3 deposition.

They also find that the modulation of Xist transcription by SETDB1/HUSH is temporally limited to the early stages of differentiation, suggesting that this feedback mechanism is critical for fine-tuning Xist RNA levels during initiation, but other regulatory pathways are involved in later maintenance phases.

Overall, the authors have identified a novel feedback control mechanism whereby SETDB1 and the HUSH complex suppress Xist RNA transcription via H3K9me3 deposition, thereby modulating Xist RNA abundance and ensuring precise regulation of X chromosome inactivation. This is a very impressive study and their results provide new insights into the epigenetic regulation of Xist and highlight the complexity of feedback loops that maintain XCI fidelity.

Major points
This is an important paper and I would strongly recommend publication in this journal. I have some suggestions to improve the clarity of the manuscript and queries which I hope the authors can comment on – though I’m not necessarily requesting

further experiments:

1 The manuscript contains extensive genomic data and multiple clones tested, which is a strength but also makes it a complex read. Some figures and supplementary data would benefit from clearer summaries and highlighting of key findings for improved accessibility for a broader audience. It would also be helpful if certain aspects of the manuscript could be given a more detailed explanation. At present it is assumed that the reader has a detailed knowledge of XCI – and the manuscript would reach a wider audience if written for an informed but more generalist reader. For example, the fact that they have two X-chromosomes (Castaneus and 129) is complex and how they distinguish reads between the two X-chromosomes is also complex. I didn't understand why reads from 'total' weren't the sum of reads from Castaneus and 129 i.e. In the histograms of H3K9me3 in Fig 4b/c, why isn't the total K9me3 the sum of the individual chromosomes – and why is it less in 4c than 4b? – maybe this relates to reads which can be assigned on the basis of SNPs – but some explanation would be helpful. There are other similar examples where improving reader accessibility would be helpful and appreciated.

2 Most of their functional assays are performed in induced or biased monoallelic systems (TetOn iXist lines and TsixStop), so prevention of biallelic upregulation and enforcement of choice are not tested. This is a concern as my understanding is that random XCI involves biallelic Xist initiation that transitions to monoallelic expression through negative feedback mechanisms – I assume this is bypassed by their artificial systems and I can see why this is difficult to overcome - can the authors comment on this?

3 Similarly, the effect of acute SETDB1 loss on silencing kinetics is not tested under native Xist promoter control or in vivo, potentially limiting generalisation of their system beyond the inducible embryonic stem cell system. While Figure 1b and Figure 1c show clear acceleration in the inducible system, Figure 4c and Extended Data Fig. 3d address chromatin marks and baseline behavior in native promoter models but not silencing kinetics after SETDB1 depletion. Although SETDB1's importance for X-inactivation has been demonstrated in MEFs and differentiating ES cells, and silencing kinetics have been characterized under endogenous Xist expression, I'm not aware of studies that have directly tested acute SETDB1 loss effects on silencing kinetics in native embryonic contexts. This is important as promoter architecture and developmental context can influence Xist regulation and XCI dynamics. Can the authors could comment on this?

4 The authors provide limited mechanistic detail on the proposed secondary SETDB1 recruitment pathway: While they show that HUSH is important for SETDB1 recruitment to the Xist locus, they also note an incomplete loss of H3K9me3 upon HUSH depletion and exclude TRIM28/KAP1 as a contributor. The putative secondary recruitment pathway(s) for SETDB1 remain unidentified – do they have any evidence for other recruiters – or can they comment on this?

Other points

The modulation of Xist transcription by SETDB1/HUSH appears restricted to early differentiation stages and is diminished later. While this is acknowledged and the authors suggest alternative mechanisms to maintain Xist at later stages, it would be helpful if they could expand on this?
Their findings suggest elevated Xist RNA leads to "spill-over" onto autosomes which may affect autosomal gene regulation. Do they have further information on this – have they mapped spill-over sites – what are the transcriptional changes on autosomes?
The elevated Xist RNA causes acceleration in X-linked gene silencing. Figures 1b–c and 2a–f establish a strong correlation, and Figure 7d–e shows the correlation recurs with HUSH depletion, they aren't able to reduce Xist back to baseline in the depleted background nor elevate Xist in a SETDB1-proficient background to test causality. They don't perform any rescue experiments so their claim that acceleration results from elevated XistRNA is inferential and has not demonstrated and should perhaps be described as such.

With regards the doxycycline inducible promoter driving Xist expression on the Mus castaneus (Cast) derived X chromosome - Is the endogenous Xist still present – this needs a better explanation to understand the system

However, the pausing index (the ratio of the read density of promoter proximal RNAPII to the read density of gene body RNAPII) is reduced following SETDB1depletion (Extended Data Fig. 2c), suggesting that an increased elongation rate also contributes to higher levels of Xist RNA -Can they provide further explanation for this statement

Do they understand why they see increased KAP1 on Xist - even though it does not appear to be SETDB1 dependent? – presumably they don't see any functional consequences?

L288 – Thus, we speculate that heterochromatinization of internal regulatory sequences is an important mechanism by which RNA-dependent recruitment of HUSH can function in genome defence - I didn't understand what they are suggesting here?

The main reason for HUSH recruitment to Xist is the presence of two remarkably long exons (Exon 1 (~9 kb) and Exon 7 (~3–4 kb) – particularly exon 1. They could emphasise further that exon 1 is almost certainly responsible for HUSH recruitment.

Reviewer #2

(Remarks to the Author)
Almeida and colleagues report the unexpected and important discovery of a role for the HUSH complex in repressing Xist expression at the early stages of X-chromosome inactivation. The study drew its major conclusions from highly quantitative genomic assays (calibrated ChIP and 4sU-seq) applied to wild-type and dTAG-induced knockout cells of SETDB1, KAP1 (TRIM28), MPP8, and TASOR, and examined Xist expression upon loss of SETDB1 in cells in which Xist is driven from a doxycline-inducible promoter as well as by its endogenous promoter, over a timecourse of differentiation.

The data were very clear and so were the conclusions of the study. The findings should be of interest to those studying lncRNAs as well as the targeting and functions of HUSH.

I would request that the authors report suppmental tables that contain the gene-by-gene outputs, potentially also including TEs, of their differential expression analyses, enabling readers to investigate the effects of dTAG-induced knockout without having to reanalyze the data themselves.

Beyond this minor request, I do note that the ratio of font-to-image size in several figures was rather small; the authors could consider adjusting these ratios to improve the readability of their manuscript.

Reviewer #3

(Remarks to the Author)
The manuscript entitled "SETDB1/HUSH modulates Xist RNA levels during establishment of X chromosome inactivation" by Almeida et al. investigates the regulation of the Xist gene at the initiation of X chromosome inactivation in mouse embryonic stem cells. The authors observe that Xist transcription initiation and elongation is appx. 4-fold increased after degron mediated depletion of the histone H3 lysine 9 methyltransferase SETDB1. From this a repressive effect of the HUSH complex on Xist transcription is elaborated showing that MPP8 but not KAP1 are involved. Importantly, establishment of H3 lysine 9 methylation at the Xist locus can be shown to be associated with Xist transcription. This is a notable and surprising result that would not have been anticipated by earlier models of Xist regulation. It contrast the previous view has been that Xist is repressed on Xa by a counting mechanism but activated on Xi. Therefore, recruitment of repressors of the Xist gene to Xi is an important discovery for understanding mammalian dosage compensation and the enigmatic mechanisms of Xist RNA localization to Xi. Overall the manuscript is well written and conclusions are drawn from clear data that are of high technical quality. Some limitations might come from experimental designs that necessarily rely on inducible Xist expression. However, endogenous Xist gene regulation is difficult to assess due to effects of HUSH depletion on differentiation and viability. Hence, the experimental opportunities might have been largely exhausted. The results will be of high interest to the research community in X chromosome inactivation and gene regulation in stem cells. A few points should be addressed for further strengthening the conclusions and clarifying some aspects of the manuscript.

Specific points

1. The study defines an RNA mediated mechanism of HUSH recruitment and separates this from KAP-1. Nonetheless, it would be interesting to further separate Xist regulatory and other essential roles of SETDB1. References to recent work on SMCHD1 and SETDB1 in maintaining gene silencing on Xi and also the authors own observation in text, line 96ff raise the question if there are hints to RNA sequences or RNA binding activities known that would be predicted to contribute to function of HUSH in Xist regulation in embryonic stem cells? As the authors previously generated repBC mutant Xist with potential SMCHD1 (and possibly HUSH) recruitment defects could provide an opportunity to isolate HUSH functions on Xist expression.

2. H3K9 methylation was found exclusively associated with the actively transcribed Xist locus (on Xi). Could the authors confirm that H3 lysine 9 methylation is absent on the inactive Xist locus (on the future Xa). A brief discussion and/or reconciliation with previous work implicating DNA methylation and Mbd2 eg Barr et al. DOI: 10.1128/MCB.02204-06, in Xist repression on Xa would be interesting. Xist repression by DNA methylation (on Xa) would then be independent on H3 lysine 9 methylation? Or is the differential K9 methylation on the active Xist locus (on Xi) specific for embryonic stem cells.

3. Line 210: There might still be residual KAP1, albeit below detection, remaining in the cells after depletion. Western analysis in Fig 6b shows a strong reduction but a faint band might be visible. I wonder if KAP1 could be quantified better to rule this out when there is no effect. Alternatively, a brief statement on potential residual protein could be included.

4. The effects of SETDB1 depletion on Xist expression are quantitatively clear but Figure 2C shows Xist clusters form without SETDB1 somewhat taking away impact. The authors suggest that Xist overexpression causes localization to autosomes. Showing the autosomal localization more directly would strengthen the conclusion of SETDB1/HUSH as an important regulator of X inactivation.

5. The text on page 267 discusses an prominent upregulation of LINE1 elements and other genes after SETDB1 depletion consistent with earlier work. Was the expression of the tetracycline regulated activator also affected by HUSH depletion? This should be considered for the interpretation as it would be immediately relevant to Xist expression.

Minor points

a) The manuscript proposes a mechanism of repressing Xist transcription. In this context another regulator, mouse Tsix, would be of interest. Could data on Tsix expression be included in the figures along with Xist to show its regulation in the

different cells and conditions. Is it reciprocally regulated? This could be important to clarify as the discussion informs of a mechanism of HUSH recruitment by intron-less transcripts and Tsix would be such a transcript of considerable length.

b) In the quantification of H3K9me3 in Fig 4 there is substantially difference between mESC and NPCs in panel b but hardly any in c. Is the low amount of K9 methylation in b from the different cell lines or a difference between Cast and 129 X chromosomes? A brief explanation could be added in the legend.

c) A long half-like of Xist on Xi measured after transcriptional arrest and observation of loss during mitosis led to a view that Xist RNA turns over in the cell when not chromosomally bound. Some kinetics measurements by FLIP/FRAP DOI: 10.1091/mbc.E11-02-0146 seem to also implicate turnover of chromosomally bound Xist in explaining the restricted steady state abundance. This view is different to the model the authors propose whereby transcription of Xist would cease once Xi is occupied. It would be interesting to hear to what extent the authors rule out ongoing displacement of Xi bound Xist (outside mitosis) and conversely is HUSH based repressive feedback on Xist transcription sufficient to block Xist transcription once Xi is occupied. Furthermore, Xist transcription would be predicted to be essential during S-phase when the X sequences replicate and new binding sites need to be occupied, and right after mitosis when Xist had been displaced and Xi is devoid. Would SETDB1 or K9 methylation mediated repression at these time points in the cell cycle?

d) The study implicates SETDB1 in a feedback mechanism regulating Xist expression for limiting the amount of Xist RNA on Xi, but it was not obvious for me how the amount of Xist RNA on Xi can be quantified/ integrated for feedback onto Xist transcription. From the discussion I understand that HUSH repression is suggested as a consequence of transcribing the two large Xist exon 1 and 8 which is supported by previous work on intron-less genomic repeat transcripts. However, this would not provide "feedback" from distant Xi binding sites of Xist. The spatial separation of Xist dots in the super-resolution images would suggest a "feedback" need likely be transmitted over some distance and make a direct contact to the Xist transcription site less convincing. It would be interesting to the reader to have the authors thoughts on a potential transmission mechanisms from these distant bound Xist and how "signals" might tell HUSH to repress Xist. Or if there is a different explanation which I might have missed.

Version 1:

Reviewer comments:

Reviewer #1

(Remarks to the Author)
I thank the authors for making the appropriate changes, and I would now wholeheartedly recommend publication of this important manuscript

Reviewer #2

(Remarks to the Author)
The authors have responded to my simple requests and also to requests from the other reviewers, which were for the most part revolving around increasing clarity of explanation. I can support publication at this stage.

Reviewer #3

(Remarks to the Author)
The revised version of the manuscript " SETDB1/HUSH modulates Xist RNA levels during establishment of X chromosome inactivation" by Almeida et al. that have further strengthened the authors study. In particular, the authors show that HUSH recruitment is specific for the expressed Xist locus on Xi and has no effect on DNA methylation on Xa where Xist is repressed. This clarification is important in my view as it allows to clearly discern the reader which mechanisms on Xa and Xi regulate Xist. New data on pages 6,11, and Extended Data Fig 2ab and 7f show that expression of the transactivator remained unaffected by Setdb1 degradation, which rules out a technical effect and strengthens the conclusions. The authors also have investigated the regulation of Tsix, which is independent and likely downstream of Xist. In addition, data is quoted of autosomal effects of "spill-over" of Xist, which to my mind is very strong evidence for the biological relevance of HUSH based repression. The authors will present the details as part of an independent manuscript, but anticipating substantiates the importance of the discovered mechanism of HUSH action on Xist. In conclusion, changes in the revised version have fully addressed the points I have raised on the original version through clarifications in the text and improved data presentation. Throughout the experimental data is of high technical quality supporting the conclusions drawn by the authors. This study discovers a, thus far unrecognized, mechanism of Xist regulation that will have a substantial impact for the understanding mammalian dosage compensation and chromatin regulation.

Minor points

a) Last paragraph of the discussion (line 323) "Here it is worth noting that the modulation of Xist transcription by SETDB1 doesn’t appear to function after early stages of differentiation, " appears to diverge from the conclusion of the results (line 114) "This indicated a marked 4-5 fold increase in Xist RNA levels in independent mESC lines (Fig. 2a), and similarly, significantly elevated levels of Xist RNA during the first 7 days of NPC differentiation (Fig. 2b)".

Open Access This Peer Review File is licensed under a Creative Commons Attribution 4.0 International License, which permits use, sharing, adaptation, distribution and reproduction in any medium or format, as long as you give appropriate credit to the original author(s) and the source, provide a link to the Creative Commons license, and indicate if changes were made.
In cases where reviewers are anonymous, credit should be given to 'Anonymous Referee' and the source.
The images or other third party material in this Peer Review File are included in the article’s Creative Commons license, unless indicated otherwise in a credit line to the material. If material is not included in the article’s Creative Commons license and your intended use is not permitted by statutory regulation or exceeds the permitted use, you will need to obtain permission directly from the copyright holder.
To view a copy of this license, visit https://creativecommons.org/licenses/by/4.0/

We thank the reviewers for their well-considered and constructive suggestions and respond to these as detailed below;

Reviewer #1 (Remarks to the Author):

This is an interesting and important study in which Almeida and colleagues investigate the role of the histone methyltransferase SETDB1 and the HUSH complex in regulating Xist RNA levels during the establishment and maintenance of X chromosome inactivation (XCI). Xist RNA plays a central role in initiating XCI and ensures transcriptional silencing of one of the two X chromosomes in females to achieve dosage compensation. Previous work has suggested that SETDB1 contributes to the maintenance of XCI through deposition of the repressive histone mark H3K9me3, but the mechanistic details and its potential role during XCI initiation remain unclear.

Using degron-based rapid depletion in mouse embryonic stem cells (mESCs) and engineered dox-inducible Xist expression, the authors demonstrate that SETDB1 depletion accelerates X-linked gene silencing during XCI establishment. This accelerated silencing was unexpected and correlates with a marked increase in Xist RNA levels (~ 4-5 fold), detected through chromatin-associated RNA sequencing (ChrRNA-seq) and confirmed by RNA-FISH super-resolution imaging. The spatial distribution of Xist RNA was largely confined to the X chromosome but shows some 'spill-over' onto neighbouring autosomes.

Further analyses show that the elevated Xist RNA levels result from increased transcription rather than RNA stabilization. Measurement of nascent RNA synthesis (4sU-seq) shows a marked increase in Xist transcription rates following SETDB1 depletion. Calibrated chromatin immunoprecipitation sequencing (cChIP-seq) for RNA polymerase II (RNAPII) shows increased occupancy and decreased promoter-proximal pausing at the Xist locus, indicating enhanced transcriptional elongation in the absence of SETDB1.

The study shows that SETDB1 mediates transcription-dependent deposition of H3K9me3 across the expressed Xist allele, and is essential for repressing Xist transcription to maintain appropriate RNA levels. This H3K9me3 deposition is also found to be dependent on the HUSH repressor complex, specifically its core subunits MPP8 and TASOR, which recruit SETDB1 in a nascent RNA-dependent manner. In contrast, they find that KAP1, a known SETDB1 adaptor is dispensable for H3K9me3 deposition and Xist regulation. Depletion of HUSH components also results in reduced H3K9me3 at the Xist locus, elevated Xist RNA levels, and accelerated X-linked gene silencing. However, HUSH-depletion does not entirely phenocopy SETDB1 depletion – their results suggest that other players may also be involved in H3K9me3 deposition.

They also find that the modulation of Xist transcription by SETDB1/HUSH is temporally limited to the early stages of differentiation, suggesting that this feedback mechanism is critical for fine-tuning Xist RNA levels during initiation, but other regulatory pathways are involved in later maintenance phases.

Overall, the authors have identified a novel feedback control mechanism whereby SETDB1 and the HUSH complex suppress Xist RNA transcription via H3K9me3 deposition, thereby modulating Xist RNA abundance and ensuring precise regulation of X chromosome inactivation. This is a very impressive study and their results provide new insights into the epigenetic regulation of Xist and highlight the complexity of feedback loops that maintain XCI fidelity.

Major points
This is an important paper and I would strongly recommend publication in this journal. I have some suggestions to improve the clarity of the manuscript and queries which I hope the authors can comment on – though I'm not necessarily requesting further experiments:

1 The manuscript contains extensive genomic data and multiple clones tested, which is a strength but also makes it a complex read. **Some figures and supplementary data would benefit from clearer summaries and highlighting of key findings for improved accessibility for a broader audience.** It would also be helpful if certain aspects of the manuscript could be given a more detailed explanation. At present it is assumed that the reader has a detailed knowledge of XCI – and the manuscript would reach a wider audience if written for an informed but more generalist reader. For example, the fact that they have two X-chromosomes (Castaneus and 129) is complex and how they distinguish reads between the two X-chromosomes is also complex. I didn't understand why reads from 'total' weren't the sum of reads from Castaneus and 129 i.e. In

the histograms of H3K9me3 in Fig 4b/c, why isn't the total K9me3 the sum of the individual chromosomes – and why is it less in 4c than 4b? – maybe this relates to reads which can be assigned on the basis of SNPs – but some explanation would be helpful. There are other similar examples where improving reader accessibility would be helpful and appreciated.

We thank the reviewer for highlighting the issue of clarity and we have addressed this with modifications to the figure legends and text that we hope achieve the aim of improving accessibility to readers who are less familiar with the assay systems we use to analyse X inactivation. To clarify the specific point about allelic reads not adding up to total reads, this is because a significant number of reads do not encompass a defined SNP between Cast and 129 strains and are therefore discarded when we apply our computational pipelines for allelic analysis. The reason values are less in 4c than in 4b is differences between inducible and native promoter expression. Lower H3K9me3 accumulation using the native promoter occurs as a result of expression being heterogeneous on a cell to cell basis, and hence lower on average compared to the inducible system.

2 Most of their functional assays are performed in induced or biased monoallelic systems (TetOn iXist lines and TsixStop), so prevention of biallelic upregulation and enforcement of choice are not tested. This is a concern as my understanding is that random XCI involves biallelic Xist initiation that transitions to monoallelic expression through negative feedback mechanisms – I assume this is bypassed by their artificial systems and I can see why this is difficult to overcome - can the authors comment on this?

We agree that the question of whether or not SETDB1/HUSH depletion might influence choice and/or mono-allelic Xist upregulation is an interesting one. It is the case that in human embryos there is initially biallelic Xist expression during initiation of X inactivation and that this also occurs for the native Xist promoter in a small proportion of mouse XX ES cells. It is less clear that biallelic Xist expression occurs in cells of mouse embryos. A proper investigation of this question is outside the scope of our current study, but we have added a sentence in the discussion to highlight this point (lines 267-8).

3 Similarly, the effect of acute SETDB1 loss on silencing kinetics is not tested under native Xist promoter control or in vivo, potentially limiting generalisation of their system beyond the inducible embryonic stem cell system. While Figure 1b and Figure 1c show clear acceleration in the inducible system, Figure 4c and Extended Data Fig. 3d address chromatin marks and baseline behavior in native promoter models but not silencing kinetics after SETDB1 depletion. Although SETDB1's importance for X-inactivation has been demonstrated in MEFs and differentiating ES cells, and silencing kinetics have been characterized under endogenous Xist expression, I'm not aware of studies that have directly tested acute SETDB1 loss effects on silencing kinetics in native embryonic contexts. This is important as promoter architecture and developmental context can influence Xist regulation and XCI dynamics. Can the authors could comment on this?

We tried to investigate the effects of SETDB1 depletion in the TsixStop XX ES cells with native Xist promoter by generating SETDB1 degron lines is this background but all of the clones we obtained were uninformative due to issues with X chromosome elimination and poor differentiation. We have added a sentence in the discussion to highlight that the dependence of allelic H3K9me3 on SETDB1 in the TsixStop model was not tested directly (line 271).

4 The authors provide limited mechanistic detail on the proposed secondary SETDB1 recruitment pathway: While they show that HUSH is important for SETDB1 recruitment to the Xist locus, they also note an incomplete loss of H3K9me3 upon HUSH depletion and exclude TRIM28/KAP1 as a contributor. The putative secondary recruitment pathway(s) for SETDB1 remain unidentified – do they have any evidence for other recruiters – or can they comment on this?

The two most widely studied interactors for SETDB1 in mammalian cells are the HUSH complex and KAP1 which as noted, were tested in the current manuscript. There is no evidence available suggesting that SETDB1 could be recruited directly by Xist RNA (see for example ref 12, Chu et al 2015). Two less well known SETDB1 interactors reported in the literature are RESF1 and ARL14EP. We tested if either of these two factors could be responsible for residual H3K9me3 on the expressed Xist locus in HUSH mutants by generating constitutive knockout of each factor in FKBP12^{F36V}-MPP8 and FKBP12^{F36V}-TASOR mESC. In both cases residual H3K9me3 remained (see

reviewer Figure 1 below). Accordingly, the identity of the secondary pathway remains an unresolved question at this time.

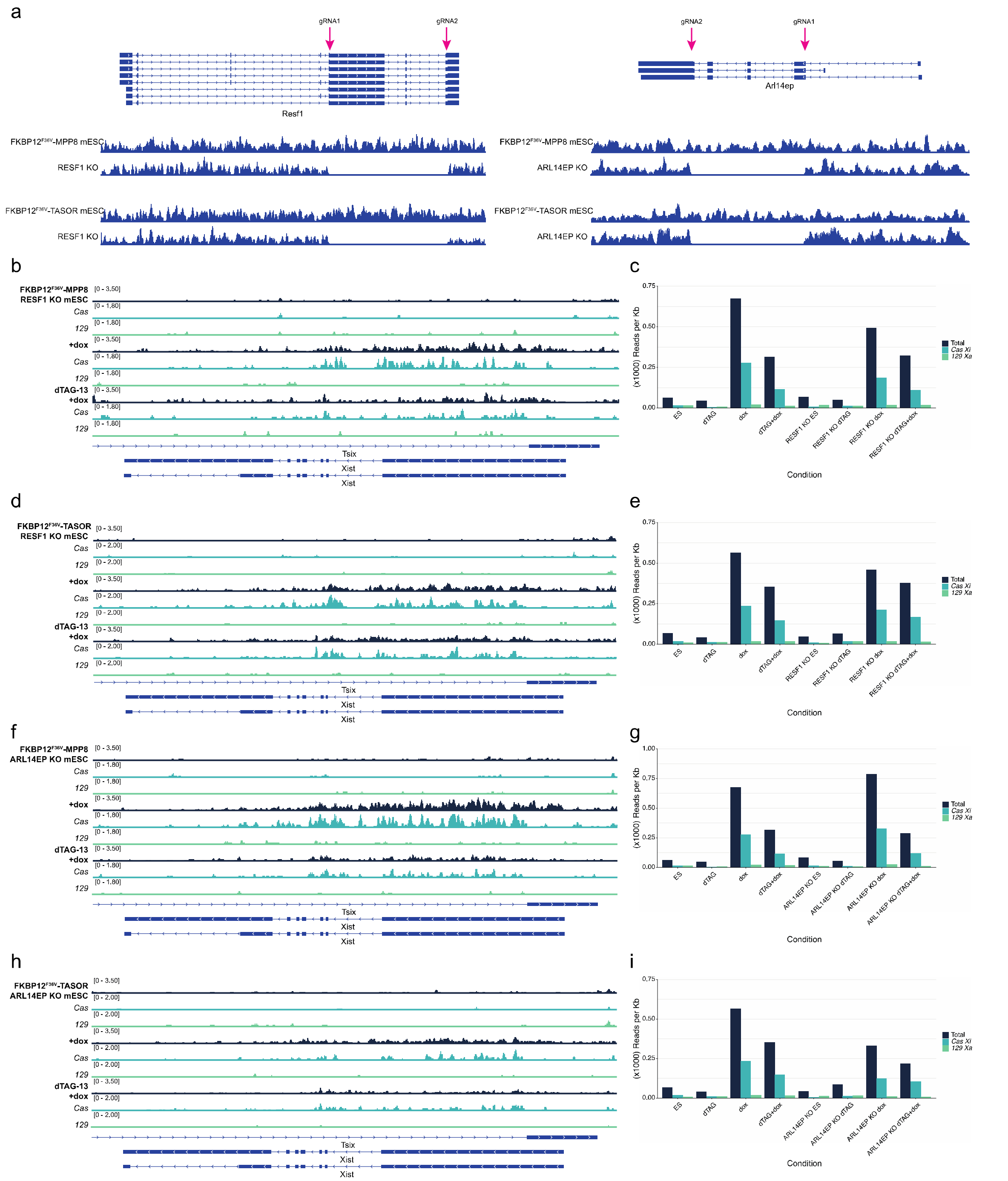

Reviewer Figure 1.
A. Confirmation of CRISPR/Cas9-mediated deletion of either RESF1 (left) or ARL14EP (right) in FKBP12^{F36V}-tagged MPP8 and TASOR cell lines using ChIP input reads.
B/C. Allelic H3K9me3 analysis in RESF1 null cells in presence or absence of MPP8
D/E. Allelic H3K9me3 analysis in RESF1 null cells in presence or absence of TASOR
F/G. Allelic H3K9me3 analysis in ARL14EP null cells in presence or absence of MPP8
H/I. Allelic H3K9me3 analysis in ARL14EP null cells in presence or absence of TASOR
All data was obtained after 1 day of Xist induction and is presented as described for manuscript Figures.

Other points

The modulation of Xist transcription by SETDB1/HUSH appears restricted to early differentiation stages and is diminished later. While this is acknowledged and the authors suggest alternative mechanisms to maintain Xist at later stages, it would be helpful if they could expand on this?

We do not have any information on alternative mechanisms that could function in later differentiation stages. Transcription-dependent mechanisms are plausible but we are not aware of specific pathways that could function in this scenario. It is probably relevant that chromosome silencing by Xist is restricted to an early window in development and that induced Xist expression in differentiated cells does not result in chromosome silencing.

Their findings suggest elevated Xist RNA leads to "spill-over" onto autosomes which may affect autosomal gene regulation. Do they have further information on this – have they mapped spill-over sites – what are the transcriptional changes on autosomes?

We do indeed infer "spill-over" of Xist onto autosomes and we have clear and direct evidence for this in an independent study that we are currently preparing for submission. Whether or not "spill-over" results in autosomal gene silencing is another question. We examined this possibility by comparing differentially expressed genes (DEGs) between Control_vs_dTAG and Dox_vs_dTAGDox, which allows us to identify Xist-dependent DEGs (we have added these called DEGs as Supplementary Table 1, line 132). As expected, Xist-dependent DEGs (106) are enriched on the X chromosome (27/106), whereas only a relatively small number (79/106) are found randomly across the 19 autosomes. These autosomal DEGs could reflect indirect downstream effects of X-linked gene regulation or, alternatively, limited Xist spill-over onto specific sites on autosomes. The only site where we observed clustered DEGs is on chromosome 4 but in this instance the genes are upregulated and therefore unlikely to be linked to targeted by Xist spillover. Our results indicate that Xist spillover does not result in stable gene-silencing of specific autosomal genes.

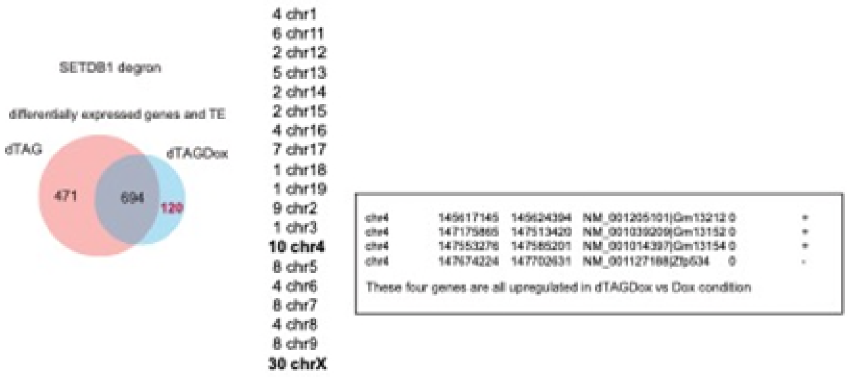

The elevated Xist RNA causes acceleration in X-linked gene silencing. Figures 1b–c and 2a–f establish a strong correlation, and Figure 7d–e shows the correlation recurs with HUSH depletion, they aren't able to reduce Xist back to baseline in the depleted background nor elevate Xist in a SETDB1-proficient background to test causality. They don't perform any rescue experiments so their claim that acceleration results from elevated XistRNA is inferential and has not demonstrated and should perhaps be described as such.

Indeed, we state in the title to the section describing elevated Xist levels that this is correlative evidence. To further underline this point we have modified the text to state that this is an inference (line 118). It is relevant to consider that in a recently published study we observed accelerated silencing correlating with increased levels of Xist RNA following depletion of factors in the m6A/NEXT pathway that promotes Xist RNA turnover (Wei et al, NSMB, 2025)

With regards the doxycycline inducible promoter driving Xist expression on the Mus castaneus

(Cast) derived X chromosome - Is the endogenous Xist still present – this needs a better explanation to understand the system

For this cell line we knocked-in the TetON promoter to drive the endogenous Xist gene on Cast allele, therefore the endogenous gene is intact, processed and expressed correctly. All of the cell lines we use are described in more detail in prior publications that we reference herein but we agree that it is important to make this clear so we have added additional explanation in the text (line 74-5).

However, the pausing index (the ratio of the read density of promoter proximal RNAPII to the read density of gene body RNAPII) is reduced following SETDB1depletion (Extended Data Fig. 2c), suggesting that an increased elongation rate also contributes to higher levels of Xist RNA -Can they provide further explanation for this statement

We agree this was unclear and have reworded this part of the paragraph (lines 146-152)

Do they understand why they see increased KAP1 on Xist - even though it does not appear to be SETDB1 dependent? – presumably they don't see any functional consequences?

The increase in KAP1 on Xist is SETDB1 dependent as shown in figure 6a suggesting the protein accumulates over the Xist locus as a 'passenger' with SETDB1 and not via a direct recruitment mechanism. However, we show that depletion of KAP1 has no significant impact on H3K9me3 accumulation over the Xist locus or it's expression and subsequent X chromosome silencing as shown in figures 6c-e.

L288 – Thus, we speculate that heterochromatinization of internal regulatory sequences is an important mechanism by which RNA-dependent recruitment of HUSH can function in genome defence - I didn't understand what they are suggesting here?

We are arguing that transcript dependent recruitment of HUSH is theoretically going to have more of an impact on promoters and cis-regulatory elements that are located within the transcription unit compared to those located upstream or downstream of the gene. This provides a rationale for selective effects on invasive DNA elements (transposons and LTR elements) which have regulatory elements within the transcribed unit. We have simplified the sentence relating to this argument (line 299-305)

The main reason for HUSH recruitment to Xist is the presence of two remarkably long exons (Exon 1 (~9 kb) and Exon 7 (~3–4 kb) – particularly exon 1. They could emphasise further that exon 1 is almost certainly responsible for HUSH recruitment.

Evidence for exon 1 being the preferential target for HUSH is the higher ChIP signal compared to exon 7, but there could be other factors at play, for example sequence content.

Reviewer #2 (Remarks to the Author):

Almeida and colleagues report the unexpected and important discovery of a role for the HUSH complex in repressing Xist expression at the early stages of X-chromosome inactivation. The study drew its major conclusions from highly quantitative genomic assays (calibrated ChIP and 4sU-seq) applied to wild-type and dTAG-induced knockout cells of SETDB1, KAP1 (TRIM28), MPP8, and TASOR, and examined Xist expression upon loss of SETDB1 in cells in which Xist is driven from a doxycline-inducible promoter as well as by its endogenous promoter, over a timecourse of differentiation.

The data were very clear and so were the conclusions of the study. The findings should be of interest to those studying lncRNAs as well as the targeting and functions of HUSH.

I would request that the authors report suppmental tables that contain the gene-by-gene outputs, potentially also including TEs, of their differential expression analyses, enabling readers to investigate the effects of dTAG-induced knockout without having to reanalyze the data themselves.

We thank the reviewer for this suggestion. We have now provided a supplemental table reporting gene-by-gene differential expression results, including transposable elements, called by using *TEtranscripts*. The table contains three sheets—Control_vs_dTAG_ChrRNA, Dox_vs_dTAGDox_ChrRNA, and Dox_vs_dTAGDox_4sU20min—which together allow readers to examine the effects of dTAG-induced knockout without the need to reanalyze the raw data.

Beyond this minor request, I do note that the ratio of font-to-image size in several figures was rather small; the authors could consider adjusting these ratios to improve the readability of their manuscript.

We have amended this point in all Figures.

Reviewer #3 (Remarks to the Author):

The manuscript entitled "SETDB1/HUSH modulates Xist RNA levels during establishment of X chromosome inactivation" by Almeida et al. investigates the regulation of the Xist gene at the initiation of X chromosome inactivation in mouse embryonic stem cells. The authors observe that Xist transcription initiation and elongation is appx. 4-fold increased after degron mediated depletion of the histone H3 lysine 9 methyltransferase SETDB1. From this a repressive effect of the HUSH complex on Xist transcription is elaborated showing that MPP8 but not KAP1 are involved. Importantly, establishment of H3 lysine 9 methylation at the Xist locus can be shown to be associated with Xist transcription. This is a notable and surprising result that would not have been anticipated by earlier models of Xist regulation. It contrast the previous view has been that Xist is repressed on Xa by a counting mechanism but activated on Xi. Therefore, recruitment of repressors of the Xist gene to Xi is an important discovery for understanding mammalian dosage compensation and the enigmatic mechanisms of Xist RNA localization to Xi. Overall the manuscript is well written and conclusions are drawn from clear data that are of high technical quality. Some limitations might come from experimental designs that necessarily rely on inducible Xist expression. However, endogenous Xist gene regulation is difficult to assess due to effects of HUSH depletion on differentiation and viability. Hence, the experimental opportunities might have been largely exhausted. The results will be of high interest to the research community in X chromosome inactivation and gene regulation in stem cells. A few points should be addressed for further strengthening the conclusions and clarifying some aspects of the manuscript.

Specific points

1. The study defines an RNA mediated mechanism of HUSH recruitment and separates this from KAP-1. Nonetheless, it would be interesting to further separate Xist regulatory and other essential roles of SETDB1. References to recent work on SMCHD1 and SETDB1 in maintaining gene silencing on Xi and also the authors own observation in text, line 96ff raise the question if there are hints to RNA sequences or RNA binding activities known that would be predicted to contribute to function of HUSH in Xist regulation in embryonic stem cells? As the authors previously generated repBC mutant Xist with potential SMCHD1 (and possibly HUSH) recruitment defects could provide an opportunity to isolate HUSH functions on Xist expression.

We find that Xist-dependent changes in H3K9me3 are limited to the expressed Xist locus and are not discernible over Xi or other chromosomes. Thus, the additional role of SETDB1 in maintenance of X inactivation (line 97…) remains unexplained. We are investigating this finding in ongoing work but stress that we believe these effects are independent of Xist gene autoregulation by SETDB1/HUSH. Work on RNA-dependent recruitment of HUSH from other labs points to a role for the periphilin subunit, although current evidence points to general sequence content features within non-intronic genes or large exon sequences. In the future it will be interesting to investigate which characteristics of Xist sequence are important and we have several engineered XX ES cell lines with deletions of different regions that could be repurposed to this end. In the case of the B/C repeat region, interpretations may be complicated by concurrent loss of Polycomb and SMCHD1 recruitment.

2. H3K9 methylation was found exclusively associated with the actively transcribed Xist locus (on Xi). Could the authors confirm that H3 lysine 9 methylation is absent on the inactive Xist locus (on the future Xa). A brief discussion and/or reconciliation with previous work implicating DNA methylation and Mbd2 eg Barr et al. DOI: 10.1128/MCB.02204-06, in Xist repression on Xa would be interesting. Xist repression by DNA methylation (on Xa) would then be independent on H3

lysine 9 methylation? Or is the differential K9 methylation on the active Xist locus (on Xi) specific for embryonic stem cells.

The reviewer refers to prior studies demonstrating an important role for the DNA methylation machinery in maintaining Xist repression on the active X chromosome. In addition to the referenced paper analysing the role of Mbd2, other studies observed similar outcomes in DNMT knockout cells. Reads for H3K9me3 over the Xist locus on inactive and active X chromosomes are quantified in figure 4. The levels on Xa are residual and largely unchanged throughout the timecourse and we would therefore infer there is no link to the DNA methylation pathway for repressing Xist on Xa. Indeed, as shown in Reviewer Figure 2 below, our available MBD-seq data indicates that DNA methylation over the Xist Xa allele is unaffected by SETDB1 depletion.

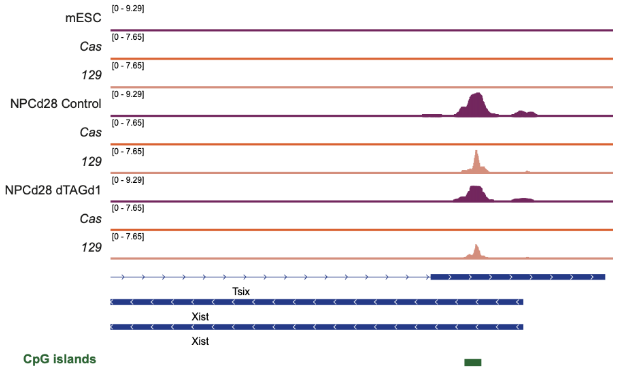

Reviewer Figure 2.
Screenshot from MBD-seq experiment in differentiated XX mESCs (inducible Xist on Cas X chromosome) in presence (control) or absence of SETDB1 depletion (dTAG d1). DNA methylation peak localising to exon I CpG island (corresponding to major Xist enhancer) occurs on active X chromosome allele in both conditions.

3. Line 210: There might still be residual KAP1, albeit below detection, remaining in the cells after depletion. Western analysis in Fig 6b shows a strong reduction but a faint band might be visible. I wonder if KAP1 could be quantified better to rule this out when there is no effect. Alternatively, a brief statement on potential residual protein could be included.

We have performed a genome-wide analysis of H3K9me3 following KAP1 depletion to verify that there is a clear phenotype. We observe very clear reductions at many of the KAP1 binding sites called using our ChIP-seq data. This data is included in the revised manuscript, Extended Data Figure 6b. Accordingly, if KAP1 were important for transcript-dependent H3K9me3 over the transcribed Xist locus we would have expected a clear difference in H3K9me3 levels.

4. The effects of SETDB1 depletion on Xist expression are quantitatively clear but Figure 2C shows Xist clusters form without SETDB1 somewhat taking away impact. The authors suggest that Xist overexpression causes localization to autosomes. Showing the autosomal localization more directly would strengthen the conclusion of SETDB1/HUSH as an important regulator of X inactivation.

As commented to reviewer 1 we have clear and direct evidence for Xist 'spill-over' onto autosomes in SETDB1 depleted cells. This experiment is part of an independent study that we are currently preparing for submission.

5. The text on page 267 discusses an prominent upregulation of LINE1 elements and other genes after SETDB1 depletion consistent with earlier work. Was the expression of the tetracycline regulated activator also affected by HUSH depletion? This should be considered for the interpretation as it would be immediately relevant to Xist expression.

We thank the reviewer for raising this important question. We have examined the expression of the tetracycline-regulated transactivator (rtTA) in both the ChrRNA-seq and 4sU-seq datasets and observed no significant changes following HUSH or SETDB1 depletion. This data has now been added to relevant Figures throughout. We conclude that observed effects on Xist RNA levels are not driven by altered rtTA levels in any of the mutants.

Minor points

a) The manuscript proposes a mechanism of repressing Xist transcription. In this context another regulator, mouse Tsix, would be of interest. Could data on Tsix expression be included in the figures along with Xist to show its regulation in the different cells and conditions. Is it reciprocally regulated? This could be important to clarify as the discussion informs of a mechanism of HUSH recruitment by intron-less transcripts and Tsix would be such a transcript of considerable length.

We don't see H3K9me3 over either allele in undifferentiated parental XX ES cells, despite Tsix being biallelically expressed. Also, unlike Xist, Tsix is comprised of small or typically sized exons and as such is not a likely candidate for recruitment of HUSH. Nevertheless, we have quantified Tsix expression across different genetic backgrounds and treatment conditions (summarized in reviewer Figure 3 below) and this indicates a progressive decrease in Tsix levels upon Xist induction, supporting an interpretation that Tsix repression is a downstream consequence of increased Xist activity rather than a reciprocally regulated or primary HUSH target.

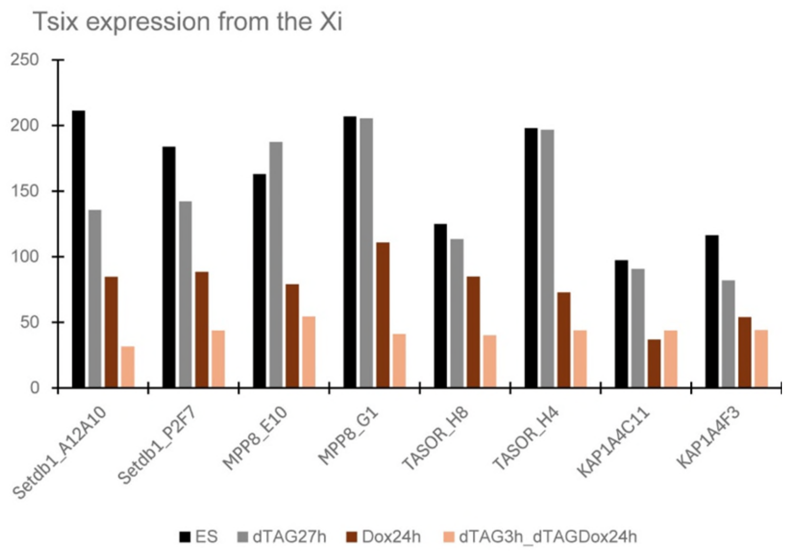

Reviewer Figure 3. Levels of Tsix RNA expression (rpm) from the Xi determined from ChrRNA-seq analysis performed in the different undifferentiated ES cell lines used in this study.

b) In the quantification of H3K9me3 in Fig 4 there is substantially difference between mESC and NPCs in panel b but hardly any in c. Is the low amount of K9 methylation in b from the different cell lines or a difference between Cast and 129 X chromosomes? A brief explanation could be added in the legend.

There are indeed differences in H3K9me3 levels in these models which we attribute largely to TsixStop having leaky Xist expression in undifferentiated ES cells and the higher level of Xist (and hence presumably SETDB1/H3K9me3) using the inducible model. We don't think that there are as significant differences in levels of expression of Xist in CAST and 129 as the reciprocal line with inducible Xist on the 129 X chromosome shows very similar effect sizes (Extended data Figure 4b,c).

c) A long half-like of Xist on Xi measured after transcriptional arrest and observation of loss during mitosis led to a view that Xist RNA turns over in the cell when not chromosomally bound. Some kinetics measurements by FLIP/FRAP DOI: 10.1091/mbc.E11-02-0146 seem to also implicate turnover of chromosomally bound Xist in explaining the restricted steady state abundance. This view is different to the model the authors propose whereby transcription of Xist would cease once Xi is occupied. It would be interesting to hear to what extent the authors rule out ongoing displacement of Xi bound Xist (outside mitosis) and conversely is HUSH based repressive feedback on Xist transcription sufficient to block Xist transcription once Xi is occupied. Furthermore, Xist transcription would be predicted to be essential during S-phase when the X sequences replicate

and new binding sites need to be occupied, and right after mitosis when Xist had been displaced and Xi is devoid. Would SETDB1 or K9 methylation mediated repression at these time points in the cell cycle?

Whilst it is clear that chromosomally bound Xist turns over slowly, there are no direct measurements on populations associated with the X chromosome vs elsewhere in the nucleus in interphase. In unpublished work using live-cell imaging we have found that 'spill-over' Xist remains chromatin associated and we see no evidence for increased turnover. We would argue that mitosis is an exceptional situation. Xist RNA fully dissociates from chromosomes and many Xist binding proteins are likely dissociated, potentially leaving transcripts vulnerable to nucleases. Thus, whilst we cant rule out models based on rapid turnover of Xist when it dissociates with the X chromosome, our current thinking is in other directions. In relation to cell cycle effects, we agree that S-phase and early G1 are key timepoints during which high levels of Xist transcription would be required and that conversely non-cycling cells would be expected to have a low rate of Xist transcription. Investigating if SETDB1/HUSH recruitment by Xist is relevant in these scenarios is non-trivial but should be interesting in the context of future work investigating the function of feedback control.

d) The study implicates SETDB1 in a feedback mechanism regulating Xist expression for limiting the amount of Xist RNA on Xi, but it was not obvious for me how the amount of Xist RNA on Xi can be quantified/ integrated for feedback onto Xist transcription. From the discussion I understand that HUSH repression is suggested as a consequence of transcribing the two large Xist exon 1 and 8 which is supported by previous work on intron-less genomic repeat transcripts. However, this would not provide "feedback" from distant Xi binding sites of Xist. The spatial separation of Xist dots in the super-resolution images would suggest a "feedback" need likely be transmitted over some distance and make a direct contact to the Xist transcription site less convincing. It would be interesting to the reader to have the authors thoughts on a potential transmission mechanisms from these distant bound Xist and how "signals" might tell HUSH to repress Xist. Or if there is a different explanation which I might have missed.

Our data indicates that H3K9me3 build up faithfully describes the Xist transcription unit, 5'-3', which implies that nascent RNAs mediate this effect. One idea we have is that occupancy of preferred binding sites on the chromosome results in over-accumulation of Xist transcripts at the transcription site, resulting in stalled nascent transcripts that then facilitate accumulation of SETDB1/HUSH and H3K9me3 deposition. Given the highly speculative nature of this (or alternative ideas) we have not included in the discussion of this paper.

We thank the reviewers for their further considerations and have made amendments to address minor comment from reviewer 3 as detailed below.

REVIEWERS' COMMENTS

Reviewer #1 (Remarks to the Author):

I thank the authors for making the appropriate changes, and I would now wholeheartedly recommend publication of this important manuscript

Reviewer #2 (Remarks to the Author):

The authors have responded to my simple requests and also to requests from the other reviewers, which were for the most part revolving around increasing clarity of explanation. I can support publication at this stage.

Reviewer #3 (Remarks to the Author):

The revised version of the manuscript " SETDB1/HUSH modulates Xist RNA levels during establishment of X chromosome inactivation" by Almeida et al. that have further strengthened the authors study. In particular, the authors show that HUSH recruitment is specific for the expressed Xist locus on Xi and has no effect on DNA methylation on Xa where Xist is repressed. This clarification is important in my view as it allows to clearly discern the reader which mechanisms on Xa and Xi regulate Xist. New data on pages 6,11, and Extended Data Fig 2ab and 7f show that expression of the transactivator remained unaffected by Setdb1 degradation, which rules out a technical effect and strengthens the conclusions. The authors also have investigated the regulation of Tsix, which is independent and likely downstream of Xist. In addition, data is quoted of autosomal effects of "spill-over" of Xist, which to my mind is very strong evidence for the biological relevance of HUSH based repression. The authors will present the details as part of an independent manuscript, but anticipating substantiates the importance of the discovered mechanism of HUSH action on Xist. In conclusion, changes in the revised version have fully addressed the points I have raised on the original version through clarifications in the text and improved data presentation. Throughout the experimental data is of high technical quality supporting the conclusions drawn by the authors. This study discovers a, thus far unrecognized, mechanism of Xist regulation that will have a substantial impact for the understanding mammalian dosage compensation and chromatin regulation.

Minor points

a) Last paragraph of the discussion (line 323) "Here it is worth noting that the modulation of Xist transcription by SETDB1 doesn’t appear to function after early stages of differentiation,

" appears to diverge from the conclusion of the results (line 114) "This indicated a marked 4-5 fold increase in Xist RNA levels in independent mESC lines (Fig. 2a), and similarly, significantly elevated levels of Xist RNA during the first 7 days of NPC differentiation (Fig. 2b)".

We corrected this issue, rewording the sentence in the discussion to specify that we are referring to timepoints after 7 days of NPC differentiation.
